# Outward Movement of Targeting Ligands from a Built‐In Reserve Pool in Nuclease‐Resistant 3D Hierarchical DNA Nanocluster for in Vivo High‐Precision Cancer Therapy

**DOI:** 10.1002/advs.202203698

**Published:** 2022-10-17

**Authors:** Weijun Wang, Yansha Gao, Yaxin Chen, Wenqing Wang, Qian Li, Zhiyi Huang, Jingjing Zhang, Qi Xiang, Zai‐Sheng Wu

**Affiliations:** ^1^ Cancer Metastasis Alert and Prevention Center Fujian Provincial Key Laboratory of Cancer Metastasis Chemoprevention and Chemotherapy State Key Laboratory of Photocatalysis on Energy and Environment College of Chemistry Fuzhou University Fuzhou 350108 China; ^2^ Key Laboratory of Laboratory Medicine Ministry of Education of China Zhejiang Provincial Key Laboratory of Medicine Genetics School of Laboratory Medicine and Life Sciences Institute of Functional Nucleic Acids and Personalized Cancer Theranostics Wenzhou Medical University Wenzhou 325035 China

**Keywords:** 3D hierarchical DNA nanocluster, built‐in reserve pool of aptamers, high precision cancer therapy, outward movement

## Abstract

Nanostructures made entirely of DNAs display great potential as chemotherapeutic drug carriers but so far cannot achieve sufficient clinic therapy outcomes due to off‐target toxicity. In this contribution, an aptamer‐embedded hierarchical DNA nanocluster (Apt‐eNC) is constructed as an intelligent carrier for cancer‐targeted drug delivery. Specifically, Apt‐eNC is designed to have a built‐in reserve pool in the interior cavity from which aptamers may move outward to function as needed. When surface aptamers are degraded, ones in reserve pool can move outward to offer the compensation, thereby magically preserving tumor‐targeting performance in vivo. Even if withstanding extensive aptamer depletion, Apt‐eNC displays a 115‐fold enhanced cell targeting compared with traditional counterparts and at least 60‐fold improved tumor accumulation. Moreover, one Apt‐eNC accommodates 5670 chemotherapeutic agents. As such, when systemically administrated into HeLa tumor‐bearing BALB/c nude mouse model, drug‐loaded Apt‐eNC significantly inhibits tumor growth without systemic toxicity, holding great promise for high precision therapy.

## Graphical abstract

An aptamer‐embedded hierarchical DNA nanocluster (Apt‐eNC) has a built‐in reserve pool in the interior cavity from which aptamers may move outward to function as needed. Even if withstanding aptamer depletion, Apt‐eNC displays a 115‐fold enhanced cell targeting and 60‐fold improved tumor accumulation. Drug‐loaded Apt‐eNC can significantly inhibit tumor growth without systemic toxicity, indicating a potential tool for precision cancer therapy.

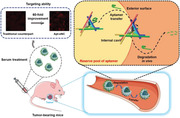

## 1 Introduction

As one of the first‐line anticancer drugs, chemotherapeutic agents have been widely used for the treatment of various types of cancers.^[^ ^1^, ^2^, ^3^ ^]^ For example, as a representative chemotherapeutic agent against a broad spectrum of malignancies approved for medical use by US FDA in the 1970s, doxorubicin (Dox) is commonly employed for the treatment of solid and nonsolid malignancies, such as breast, ovarian, uterine cancers acute leukemia and lymphomas,^[^ ^4^, ^5^ ^]^ and yields a very high cure rate (over 90%).^[^ ^6^ ^]^ However, standard chemotherapeutic agents lack active tumor‐targeting capability and usually cause the nonspecific distribution throughout the body via systemic circulation, unavoidably leading to the severe damage to healthy tissues and organs.^[^ ^7^ ^]^ To alleviate the systemic toxicity, traditional anticancer drugs require the reducing of drug doses to a level lower than the value bringing about adverse effect in normal cells, directly resulting in an insufficient dosage to tumor regions ^[^ ^8^ ^]^ and thereby compromising the inherent therapeutic efficacy.^[^ ^9^, ^10^, ^11^, ^12^ ^]^ Hence, there is an urgent need for the development of a specific tumor‐targeted drug delivery system for cancer chemotherapy. At present, various nanomaterials, such as lipids, polymers and inorganic nanomaterials, have been explored as drug delivery vehicles.^[^ ^13^, ^14^ ^]^ Unfortunately, most nanoparticle and polymeric‐nanomaterial‐based therapeutic strategies suffer from multiple drawbacks,^[^ ^15^ ^]^ including the insufficient cargo capacity, inability to engineer the spatially addressable surfaces for multifunctional activities,^[^ ^16^, ^17^ ^]^ laborious preparation procedure and safety concerns due to possible aggregation of exogenous materials, redistribution to vital organs and generation of harmful metabolites.^[^ ^18^ ^]^

As a naturally occurring biomacromolecule, DNA exhibits high water solubility, good biocompatibility and versatile functionality, enabling the cell targeting and high capacity loading of cargoes. Thus, intense efforts have been made to explore the application of artificial nanostructures made entirely of DNAs for drug delivery.^[^ ^15^, ^19^, ^20^, ^21^, ^22^, ^23^, ^24^ ^]^ In fact, DNAs are well recognized to be ideal building blocks for the self‐assembly of diverse nano‐objects in one, two and three dimensions (1D, 2D, and 3D) that are suited to implement the intended tasks,^[^ ^25^, ^26^, ^27^ ^]^ thereby allowing the scientists to develop the user‐defined DNA framework structures for biological and medical applications.^[^ ^28^, ^29^ ^]^ Moreover, some studies demonstrate that rigid and dense DNA nanostructures (e.g., DNA tetrahedral and spherical nanostructure) show the enhanced resistance to the degradation by nucleases and hold a prolonged blood circulation time, bringing the accumulation in diseased sites within the bounds of possibility.^[^ ^30^, ^31^, ^32^ ^]^

If imparting active targeting capability via functional hybridization with targeting ligands, such as aptamers and antibody‐DNA conjugates, these constructs hold particularly great promise as drug delivery vehicles for targeted cancer therapy.^[^ ^15^, ^33^ ^]^ Indeed, various kinds of targeting ligands, including antibodies, peptides, small molecules, and aptamers, have been used to develop the ligand‐conjugated drugs for specific targeting and treatment of cancers.^[^ ^34^ ^]^ However, the one‐to‐one coupling modality limits the therapeutic effect of drugs. Nucleic acid aptamers are short, single‐stranded oligonucleotides with unique intramolecular conformations and specific recognition abilities to a broad range of cognate targets.^[^ ^35^, ^36^, ^37^ ^]^ Compared with widely used antibodies, aptamers exhibit several intrinsic advantages, such as low production cost, inherent thermal stability, exquisite programmability, ease of functional modification, efficient tissue penetration and comparable or even higher binding affinity. Moreover, nucleic acid aptamers can be easily integrated into DNA nanostructures without chemical modification. Thus, quite a few endeavors have been made in search of useful aptamer‐integrated DNA nanostructures for targeted transport of chemotherapeutic drugs to suppress the toxic side effects.^[^ ^16^, ^38^, ^39^, ^40^, ^41^ ^]^ To protect the aptamers from nuclease degradation during blood circulation, the chemical modifications with various protective moieties are often employed. But these strategies could lead to the additional toxicity, severe immunogenicity and/or part loss of binding affinity.^[^ ^42^, ^43^ ^]^ Even if some nanovehicles travel a long and arduous journey and reach the intended destination in the body, the subsequent chemotherapy is frequently insufficiently effective owing to low local concentration of chemotherapeutics originating from the limited drug payload capacity.^[^ ^44^ ^]^ To improve the therapeutic efficacy, cancer treatment usually requires the repeated dosing and even long‐term use of chemotherapeutics, causing the multidrug resistance phenomenon. Because of the toxicity associated with non‐nucleic acid constituents, low drug loading capacity and/or nuclease susceptibility of targeting ligands, despite tremendous efforts, the transition of existing DNA assembly based drug delivery systems to clinical applications has been hampered. Insufficient therapy efficacy and safety concerns are the two principal limitations that lead to the failure of DNA nanostructure carriers in the stage of clinical trials. Therefore, it would be desirable to develop a versatile drug delivery carrier candidate with high biocompatibility, high cargo loading capability and desirable degradation resistant structure for preventing unintended drug leakage and enhancing in vivo tumor accumulation.

Herein, using hierarchical 3D assembly technique, we demonstrate an aptamer‐embedded DNA nanocluster (Apt‐eNC) made entirely of DNA components as a highly intelligent drug carrier for high precision cancer therapy. Based on the spatial organization of 283 functional DNA tetrahedrons (Tetra) as the structural units, besides surface‐confined aptamers with high binding affinity, Apt‐eNC is designed to have a built‐in reserve pool of aptamers capable of offering the instant compensation as needed. Because sgc8 is capable of specifically binding to a membrane protein tyrosine kinase‐7 (PTK7) often overexpressed cancerous cells ^[^ ^37^, ^45^ ^]^ and doxorubicin (Dox) is commonly used anticancer agents for the treatment of a wide variety of hematological malignancies and solid tumors,^[^ ^15^, ^43^, ^46^ ^]^ sgc8 and Dox are used as the targeting aptamer model and anticancer drug model, respectively. The aptamer is installed at the facing‐upward vertex of Tetra unit, while Dox is densely intercalated into double‐stranded (ds) DNA duplex. The as‐assembled nanostructure is called Dox‐sgc8‐eNC where aptamer‐incorporated Dox‐encapsulated DNA Tetras are crosslinked in a sequentially ordered manner. The nicks, including ones in the bottom edges of Tetra and in the crosslinking arms (protruding from triangular bottom face) between structural units, are sealed by DNA ligase, substantially rigidifying the structure of Tetra units and DNA NC backbone, and forming a built‐in reserve poor of aptamers. The resulting structural rigidification not only underlies the enhanced resistance of sgc8‐eNC carrier to enzymatic attack but also allows the sufficient space for the molecular shuttle between Tetra units. Thus, besides unwanted drug leakage is circumvented, the aptamers in internal reserve pool can transfer to the exterior surface to provide the compensation for the loss of aptamers degraded by endonucleases in the surrounding environment. Thereby, Dox‐sgc8‐eNC can magically preserve the structural integrity and high‐density aptamer layer on its surface even if circulating in the blood for a long time, leading to two orders of magnitude enhanced ability to maintain specific cell targeting and displaying dozens of times improved accumulation in target tumor tissues. Moreover, each tetrahedral structural unit can serve as a drug delivery container and sgc8‐eNC has an extraordinarily high cargo capacity (5670 Dox per vehicle). As a result, systemically administered Dox‐sgc8‐eNC can be specifically recognize and efficiently deliver anticancer therapeutics to tumor sites in the satisfactory dosage range and significantly inhibit tumor growth without apparent systemic toxicity, demonstrating a promising platform for imaging and treating the cancerous tissues or organs inaccessible for traditional delivery tools.

## 2 Results and Discussion

### 2.1 Mechanism for Persistent Tumor‐Recognition Ability of sgc8‐eNC and High‐Precision Cancer‐Targeted Drug Delivery

**Scheme 1:**
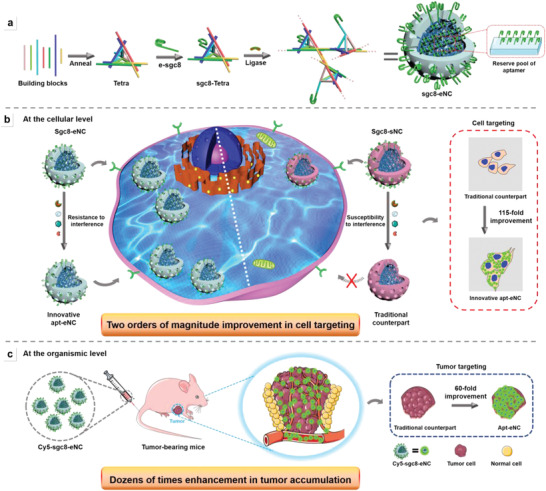
Schematic illustration of hierarchical self‐assembly of 3D sgc8‐embedded DNA nanocluster (sgc8‐eNC) for targeted drug delivery. a) Stepwise assembly of sgc8‐eNC. b) Molecular mechanism for sgc8‐eNC to maintain its cell‐targeting ability in complex biological environment. The left part shows the schematic representation of confocal laser scanning microscope (CLSM) imaging of target cells treated with sgc8‐eNC that is pretreated to extensively deplete the aptamers, while the right part shows the scheme of CLSM imaging of the same cells treated with sgc8‐sNC counterpart under identical conditions. c) In vivo tumor accumulation of Dox‐sgc8‐eNC.

Sgc8‐embedded DNA nanocluster (sgc8‐eNC) is composed of 3D cross‐linked DNA tetrahedron whose facing‐upward vertex is decorated with a standing‐up sgc8 overhang by hybridization. Scheme S1 (Supporting Information) represents the schematic illustration of DNA Tetra unit, accompanied by the base sequence of DNA components. Four sticky ends are designed at the three vertexes of triangular bottom face, while the binding site of aptamer (Apt‐binding site) is arranged at vertex iv. The sticky end‐ia can hybridize with sticky end‐ii, while the sticky end‐ib is complementary to the sticky end‐iii. The sticky end‐ia and sticky end‐ib are designed at the same vertex but point toward two different directions, forming a scissors‐shaped structure with the help of LS. Except e‐Tc, all the DNA components are phosphorylated at their 5′ end. The assembly of sgc8‐eNC is described in **Scheme** **1**a. The DNA Tetra is firstly assembled from seven building blocks. Then, sgc8 is hybridized to the Apt‐binding site at vertex iv. Afterward, the sealing of nicks by DNA ligase is conducted to further promote the advanced assembly of aptamer‐hybridized DNA Tetras and rigidify DNA assemblies, forming the rigid spherical sgc8‐eNC that is schemed as a 3D cartoon with an imaginary shell (see the enlarged one in Scheme S2a of the Supporting Information) to facilitate the understanding of structure and functions. Although the triangular bottom faces of neighboring Tetra units are not in the same plane due to the ration of DNA helix, the corresponding nick‐sealed edges (including connection arms) in neighboring Tetra are strung together, forming a 3D rigid cross‐linked DNA backbone. Apparently, DNA Tetra units are sequentially installed to the highly ordered 3D interwoven DNA backbone. Because of a much larger size than the renal clearance threshold (20 nm) ^[^ ^47^ ^]^ and a 3D DNA nanostructure with high mechanical rigidity that is considered to protect their basic components against enzymatic degradation,^[^ ^48^, ^49^ ^]^ the sgc8‐eNC holds the substantially enhanced resistance to nuclease degradation and can persistently circulate in the bloodstream, offering more opportunity to encounter the cancerous tissues.

Moreover, since the installation of aptamers proceeds before the ligation‐based sealing of nicks (Tetra is free), they can sufficiently hybridize to DNA Tetra units. As a result, there are abundant aptamers in the internal cavity and on the surface of as‐assembled clusters, forming the surface‐scatted aptamers and built‐in reserve pool of aptamers and thereby underlying unique advantages in specific target recognition. Specifically, due to the rigid structure of 3D backbone and Tetras units, there is enough space within sgc8‐eNC object for molecular shuttle between basic units. Thus, when the surface‐scatted aptamers are degraded in a complex biological milieu, the ones in the internal reserve pool can move outward to offer the compensation for the degradation‐induced ligand loss (Scheme S2b, Supporting Information), thereby maintaining the high tumor cell‐targeting capability (the left half of Scheme 1b). In contrast, if aptamer is hybridized only onto NC surface (obtaining the direct counterpart, sgc8‐sNC, where sgc8s are scattered only on the surface), no compensation occurs even if substantial degradation happens, thus losing the recognition ability and disabling the specific cellular internalization into target cancer cells (the right half of Scheme 1b). Once sgc8‐eNC is systemically administrated into tumor‐beating mice, it can persistently remain its target recognition ability/structural integrity and leave the inherent tumor accumulation properties unaffected even if circulating in the bloodstream, substantially increasing the opportunities to overcome various physiological barriers, such as enzymatic degradation, rapid elimination by renal excretion and off‐target uptake by healthy cells, and eventually reach the intended therapeutic sites (Scheme 1c). The assembly of sgc8‐sNC is illustrated in Scheme S3 (Supporting Information), during which DNA NC assembly and ligation‐mediated nick sealing are performed before the installation of targeting ligands, thus hampering the hybridization of sgc8 with the internal Apt‐binding sites due to electrostatic repulsion/steric hindrance. Compared with traditional type of sgc8‐sNC, sgc8‐eNC displays two orders of magnitude enhanced ability to preserve the specific internalization into target tumor cells at the cellular level and dozens of times enhance in tumor accumulation at the organismic level (the related data will be discussed in Figure 2 and Figure S26, Supporting Information).

It is interesting to note that, because each structural unit DNA Tetra can serve as one drug container, sgc8‐eNC holds a high capacity for loading of therapeutics agents (e.g., Dox). Once loading anticancer therapeutics, sgc8‐eNC can precisely transport the sufficient number of anticancer drugs into the appropriate tumor sites and efficiently inhibit tumor growth, accomplishing the high precision targeted cancer therapy. Meanwhile, the residual drug‐loaded sgc8‐eNCs predominantly reside in excretory organs, liver, and kidney, and is easily excreted from the body, avoiding the systemic toxicity. In addition, an array of fluorophores available for the modification of building blocks allows the imaging of in vivo tissues or organs to explore the biodistribution of NC structures and even intracellular localization. Upon drug off‐loading, the restoration of Dox fluorescence also enables the real‐time imaging to study the intracellular behaviors and pharmacokinetics (distribution, metabolism, excretion, toxicity, etc.).

### 2.2 Aptamer‐Mediated Specific Cancer Cell Recognition

**Figure 1:**
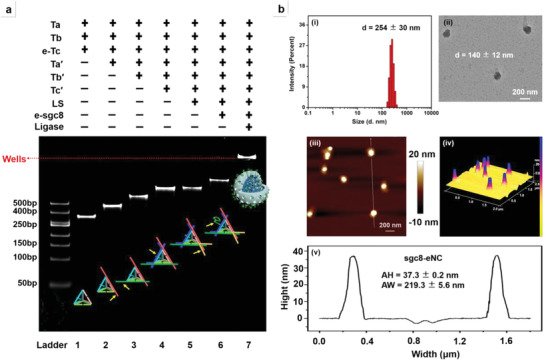
Characterization of aptamer‐embedded nanocluster (sgc8‐eNC). a) Native PAGE (nPAGE, 6%) analysis of sgc8‐eNC and relevant DNA intermediates to display the stepwise‐assembly process. The red arrow is the position of gel/well interface. b) Hydrodynamic diameter of sgc8‐eNC measured by DLS i), corresponding TEM image ii) and AFM analysis iii–v). Specifically, there are AFM image iii) of sgc8‐eNC, corresponding 3D tapping‐mode topographic image iv) and cross‐section profile v) along the white line shown in part iii. AH and AW denote the average height and average width (*n* = 20), respectively. Scale bar = 200 nm.

Construction of Sgc8‐eNC is accomplished by designing a specific binding site for sgc8 at vertex‐iv of DNA tetrahedron (Tetra) unit followed by ligation‐based sealing of nicks. In essence, sgc8‐eNC is an aptamer‐conjugated DNA nanocluster (NC) with a reserve pool of aptamers in the cavity. Thus, the fundamental framework, bare basic DNA NC without aptamers, was first assembled. Then, sgc8‐eNC and the counterpart sgc8‐sNC were separately constructed and characterized in a comparative manner. The experimental details are seen in the Section A of the Supporting Information. The experimental data from nPAGE analysis, AFM imaging, TEM imaging and DLS measurement are shown in **Figure** **1** and Supporting Figures S1–S19 (Supporting Information), while the corresponding discussion is presented in Section B.1 of the Supporting information.

To test whether sgc8‐eNC can specifically recognize PTK7‐overexpressed cancerous cells, the confocal laser scanning microscope (CLSM) imaging and flow cytometry assay were carried out to observe CEM, HeLa and Ramos cells subjected separately to fluorescently modified random DNA library (Lib), NC, Lib‐eNC (the same as sgc8‐eNC but a random base sequence is used instead of sgc8), free sgc8, sgc8‐sNC, and sgc8‐eNC under identical conditions. As shown in Figure S20a (Supporting Information), no obvious fluorescence signal is detected from Lib, NC, Lib‐eNC, and cell only, indicating no cellular internalization regardless of cells and DNA assemblies. In contrast, an obvious fluorescence signal is observed from CEM and HeLa incubated with sgc8 but not from Ramos cells, showing the efficient binding of aptamer to positive cells.^[^ ^1^, ^15^ ^]^ Moreover, incubation with sgc8‐eNC and sgc8‐sNC induces the further increase in the fluoresce brightness of cells, indicating the higher binding affinity to CEM and HeLa cells. This should be attributed to the synergistic multivalent binding effect of surface‐confined aptamers.^[^ ^50^ ^]^ Flow cytometry analysis, a direct approach capable of quantitatively assessing the cellular uptake efficiency, was also used to evaluate the binding affinity of different DNA NCs. As shown in Figure S20b (Supporting Information), the intensity of fluorescence signal from CEM and HeLa cells increases in the order of sgc8 < sgc8‐sNC < sgc8‐eNC, but Ramos cells do not show obvious fluorescence shift regardless of formulations, demonstrating that sgc8‐eNC possesses both the highest cellular uptake efficiency and desirable specificity. However, once CEM and HeLa cells were pretreated with free sgc8, both CLSM images and flow cytometry data have no detectable fluorescence signal, implying that the cellular internalization of sgc8‐eNC is mediated by cell‐surface protein receptors and targeting aptamer can guide the binding of nanostructures to target sites. Moreover, even under identical conditions, no detectable change in fluorescence intensity is observed from L02 (human normal liver) cells (Figure S21, Supporting Information), suggesting that sgc8‐eNC has the highly specific recognition ability toward target cancerous cells against healthy tissues. The universality of aptamer‐embedded DNA nanocluster is confirmed as shown in Figure S22 (Supporting Information), where AS1411‐eNC and S6‐eNC were assembled and their specific target cancer cell recognition was separately validated.

### 2.3 Movement Out of Aptamers from Built‐In Reserve Pool

One of the most attractive features of sgc8‐eNC is the enhanced resistance to enzymatic degradation in a complex biological milieu. Even sgc8‐eNC is pretreated with fresh mouse serum solution, no obvious degradation is detected (Figure S23, Supporting Information). Moreover, its target recognition ability almost can be remained unchanged on the cellular level and on the organismic level (Figures S24–S26, Supporting Information). The corresponding discussion is represented in SB.2 of Section B (Supporting Information).

The unique structural basis underlies the ability of sgc8‐eNC to maintain its superior targeting performance in complex environments. Besides the structural rigidness protects DNA components from nuclease attack, the preliminary mechanism hypothesis is that aptamers can transfer from the internal reserve pool to the exterior surface to provide the compensation for the loss of aptamers subjected to accidental enzymatic degradation, alleviating the deleterious effects encountered by traditional counterpart nanostructures (e.g., sgc8‐sNC and sgc8‐Tetra) to a great extent. Moreover, aptamers could move from the aptamer‐rich area to aptamer‐sparse area, including the transfer between different DNA NCs. The molecular mechanism was firstly explored by evaluating the hydrophobic interactions between bare NC with cholesterol moieties (i.e., Chol‐NC) and Si‐DLBM particles (DOPC liposome bilayer membrane‐coated silicon dioxide microsphere) because cholesterol‐attached DNA nanoparticles are capable of anchoring onto the surface of Si‐DLBM.^[^ ^51^, ^52^ ^]^ The signaling scheme for the single‐color fluorescence imaging and experimental results are shown in Figure S27 (Supporting Information). Chol‐NC has many cholesterol (Chol) moieties on its surface since each Tetra unit has one Chol‐e‐Tc component, and the corresponding 3D cartoon view is shown in Figure S27a (Supporting Information). When Chol‐NC was mixed with Cy5‐sgc8‐eNC, the Cy5e‐sgc8 would transfer to Chol‐NC, making Chol‐NC fluoresce (Figure S27b, Supporting Information). To differentiate original objects from their own final forms, Cy5‐sgc8‐eNC‐II and Chol‐NC‐II, during the discussion, Cy5‐sgc8‐eNC and Chol‐NC are called Cy5‐sgc8‐eNC‐I and Chol‐NC‐I, respectively. The resulting mixture is called M‐NC‐1. Cy5‐sgc8‐eNC‐I (Figure S27c, Supporting Information) and Chol‐NC‐I (Figure S27d, Supporting Information) cannot make Si‐DLBM fluoresce, which is reasonable because the former cannot adsorb onto Si‐DLBM due to the absence of cholesterol groups, while the latter can interact with Si‐DLBM but has no fluorophores. However, after treatment with M‐NC‐1, Si‐DLBM does show a strong red fluorescence signal (Figure S27c, Supporting Information), demonstrating that Cy5e‐sgc8 can get off Cy5‐sgc8‐eNC and then transfer to Chol‐NC (generating Chol‐NC‐II). When Chol‐NC is modified with FAM (obtaining Chol‐FAM‐NC‐I), it can make Si‐DLBM show the green fluorescence signal (Figure S28d, Supporting Information). Along this line, the dual‐color fluorescence imaging was performed to validate the transfer of sgc8 aptamers between DNA NCs (Figure S28a, Supporting Information). As expected, no red fluorescence signal is detected from Si‐DLBM treated with Cy5‐sgc8‐eNC in Figure S28c (Supporting Information) since Cy5‐sgc8‐eNC cannot interact with Si‐DLBM. However, Figure S28b (Supporting Information) shows that the mixture of Chol‐FAM‐NC‐I and Cy5‐sgc8‐eNC‐I can make Si‐DLBM to show both green FAM fluorescence and red Cy5 fluorescence, implying that Chol‐FAM‐NC receives Cy5e‐sgc8 after incubation with Cy5‐sgc8‐eNC. Further CLSM imaging shows that the fluorescence signal of Si‐DLBM does originate from the hydrophobic interactions between Chol‐NC and Si‐DLBM particles rather than from the nonspecific adsorption of NCs onto bare SiO_2_ microsphere (Figure S29, Supporting Information). These experimental data verify the occurrence of aptamer transfer between different NCs. Moreover, the aptamer transfer between different NCs was also validated by cell imaging (Figures S30–S32, Supporting Information).

Besides, because the outward movement of internal aptamers from the reserve pool of sgc8‐eNS onto the exterior surface is considered to impair the favorable cell targeting properties, the transfer process was validated by evaluating the cellular internalization capability after removing the surface‐confined aptamers. For this, a complementary sequence of e‐sgc8 was designed (abbreviation as Ce‐sgc8) to peel off e‐sgc8 from sgc8‐eNC (Figure S33, Supporting Information). Moreover, the ultrafiltration centrifugation‐based separation, which can be used to certify the removal of surface‐confined probes from DNA assemblies,^[^ ^53^, ^54^ ^]^ corroborates the displacement of e‐sgc8 molecules by Ce‐sgc8 from sgc8‐eNC (Figure S34, Supporting Information). Along this line, excess Ce‐sgc8 was incubate with DNA nanoclusters for different time periods, and the residual fluorescence intensity of DNA objects was detected after ultrafiltration. As shown in Figure S35 (Supporting Information), the fluorescence of F‐sgc8‐sNC almost becomes exhausted within 0.5 h and remains basically unchanged when further incubation, indicating the DNA hybridization on the surface is a rapid process. In contrast, the fluorescence intensity of F‐sgc8‐eNC gradually decreases and retains more than 30% of original fluorescence even at 4 h. A gradual DNA hybridization‐based displacement process demonstrates that, for sgc8‐eNC, aptamers are indeed arranged both onto the surface and in the internal cavity and can move outward as needed. Moreover, Figure S36 (Supporting Information) shows the difference in the internalization capability between sgc8‐eNC and sgc8‐sNC treated with different concentrations of Ce‐sgc8. When 100 × 10^−9^ m Ce‐sgc8 was added to sgc8‐eNC solution, and Ce‐sgc8/e‐sgc8 products were removed by ultrafiltration centrifugation, the uptake rate of sgc8‐eNC by target cells was reduced (Figure S36a and the left of Figure S36c, Supporting Information). However, the uptake rate of sgc8‐sNC counterpart is much lower (Figure S36d and the left of Figure S36f, Supporting Information). Upon the addition of the higher concentration of Ce‐sgc8 (200 × 10^−9^ m that equals to the concentration of e‐sgc8 in sgc8‐eNC), the amount of internalized sgc8‐eNC is slightly lower than that at 100 × 10^−9^ m (Figure S36b and the right of Figure S36c, Supporting Information), implying only partial loss of internalization ability. Under identical conditions, almost no sgc8‐sNC can enter the cells (Figure S36e and the right of Figure S36f, Supporting Information), indicating the complete displacement of e‐sgc8 and disabling aptamer‐mediated cellular internalization. This is because Ce‐sgc8 has much less opportunity to reach the internal reserve pool of sgc8‐eNC to compete for e‐sgc8. Moreover, after the removal of excess Ce‐sgc8 strands, aptamers in the built‐in reserve pool can transfer out to the exterior surface and compensate for the aptamer loss, preserving the cellular uptake. However, these processes will not happen for sgc8‐sNC counterpart.

**Figure 2:**
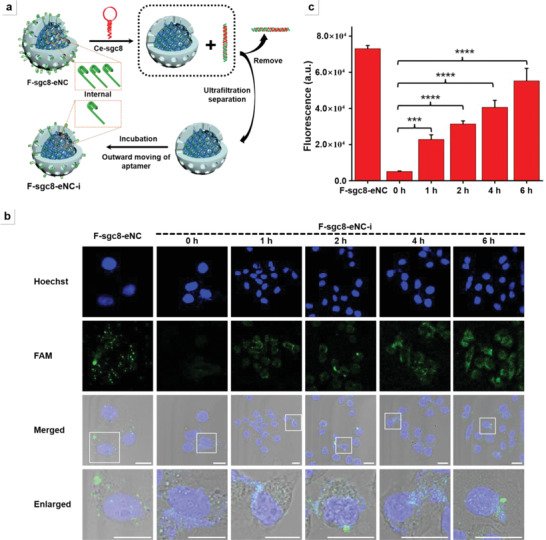
Outward movement of aptamers in the built‐in reserve pool from the interior of F‐sgc8‐eNC toward the spherical surface. a) Schematic diagram of the displacement of surface‐hybridized e‐sgc8s by the complementary strands (Ce‐sgc8) and subsequent outward movement of aptamers from internal reserve pool after ultrafiltration centrifugation and incubation for a given time interval. Tb′‐FAM was instead used during the assembly of F‐sgc8‐eNC, and the corresponding product is called F‐sgc8‐eNC‐i. b) CLSM images of HeLa cells treated with F‐sgc8‐eNC or F‐sgc8‐eNC‐i that was prepared with the following three steps: incubating F‐sgc8‐eNC with Ce‐sgc8 for 30 min, ultrafiltration centrifugation and allowing F‐sgc8‐eNC‐i to stand for 1, 2, 4, or 6 h. Scale bar = 20 µm. c) Quantitative fluorescence intensity recorded from the same cells as panel (c). The statistical analysis of multiple comparisons were evaluated by one‐way ANOVA. ***p < 0.0005, ****p < 0.0001, two‐tailed unpaired *t* test.

To convincingly demonstrate the outward movement of aptamers from the built‐in reserve pool toward the exterior surface, after peeling off the surface‐hybridized aptamers, we incubated the resulting F‐sgc8‐eNC (having Tb′‐FAM component and label‐free e‐sgc8) in working buffer for different time periods (0, 1, 2, 4, and 6 h) to allow aptamer transfer, then incubated with cells and evaluated the cellular internalization efficiency. **Figure** **2**a outlines the experimental procedure and aptamer compensation effect. Figure 2b shows that intact F‐sgc8‐eNC makes target cells emit a strong fluorescence signal. When sgc8‐eNC was treated with Ce‐sgc8 and immediately incubated with target cells, the fluorescence signal is indeed substantially compromised (0 h), proving the shedding of sgc8 aptamer from the surface of sgc8‐eNC and basically losing the cellular internalization ability. However, when preincubating sgc8‐eNC‐i for a different time periods, the fluorescence signal from the cells treated shows a trend of gradual increase, indicating that the uptake rate of sgc8‐eNC‐i was gradually restored. The quantitative assessment of fluorescence signal from different experimental groups is shown in Figure 2c. The fluorescence recovery efficiency is up to 75% at 6‐h incubation. Different from sgc8‐eNC‐i, the sgc8‐sNC‐i counterpart has no fluorescence signal recovery over the entire incubation time (Figure S37, Supporting Information), indicating the permanent loss of specific target recognition ability once its aptamers are displaced. According to the quantitative fluorescence measurement, the amount of sgc8‐eNC‐i internalized into target cells at 6‐h incubation is 115‐fold larger than sgc8‐sNC‐i under identical conditions. These experimental data demonstrate that aptamers in the built‐in reserve pool of sgc8‐eNC can indeed transfer outward when the surface‐hybridized aptamers are depleted, underlying the enhanced resistance to enzymatic digestion required by in vivo applications, and thereby making sgc8‐eNC a potential drug delivery vehicle for high precision cancer therapy.

### 2.4 Targeted Transport of Chemotherapeutic Agents and Selective Anticancer Therapy Ex Vivo

Sgc8‐eNC is internalized into target cells mainly via clathrin‐mediated endocytosis as confirmed in Figures S38 and S39 (Supporting Information), and the related discussion is represented in the SB.3 of Section B (Supporting Information). As aptamer‐embedded 3D DNA superstructures by hierarchical assembly, sgc8‐eNC has a high density of DNA components and thus allows high‐capacity loading of therapeutics.^[^ ^55^ ^]^ Figures S40 and S41 (Supporting Information) evidence that sgc8‐eNC has the 19‐ to 100‐fold enhanced drug payload capacity. No obvious difference in the surface morphology, overall shape and size is detected between sgc8‐eNC and Dox‐sgc8‐eNC (Figure S42, Supporting Information). Under different simulated physiological environments,^[^ ^15^, ^23^ ^]^ Dox‐sgc8‐eNC is stable enough to prevent drug leakage of Dox at physiological pH (Figure S43, Supporting Information) but allows an efficient drug release in acidic tumor microenvironment (Figure S44a, Supporting Information) and upon enzymatically triggered degradation (Figure S44b, Supporting Information). In addition, sgc8‐eNC is suitable for the encapsulation of other widely‐used anticancer drugs and also displays high drug payload capacity (Figure S45, Supporting Information).

Having demonstrated the cell‐specific internalization and high drug encapsulation efficiency of 3D DNA nanostructures, we next investigated the targeted transport of chemotherapeutic agents at the cellular level. Dox‐loaded sgc8‐eNC is expected to specifically enter target cells through receptor‐mediated endocytosis, followed by enzymatic degradation and Dox release. ^[^ ^56^, ^57^ ^]^ The released Doxs pass through the nuclear membrane and accumulate in cell nuclei, inhibiting DNA replication and thereby killing cells.^[^ ^58^, ^59^ ^]^ This process is outlined in Figure S46a (Supporting Information). In order to visualize the distribution of DNA nanostructures within the cells and drug release into the expected compartment, Cy5 fluorophore was attached to one of oligonucleotide components, Tb′, to synthesize two‐color fluorescence signaling nano‐object, Dox‐Cy5‐sgc8‐eNC, and HeLa cells were used as the model cells. As shown in Figure S46b (Supporting Information), cell images show that Cy5 green fluorescence signal and Dox red fluorescence signal appear at different time points and gradually increase. Specifically, within the first 2 h, weak Cy5 fluorescence signal is observed in the cytoplasm (surrounding cell nuclei), but Dox fluorescence is almost negligible. This is because Dox cannot emit fluorescence when inserting GC base pairs of double‐stranded DNA.^[^ ^60^ ^]^ With the extension of incubation time, the Cy5 green fluorescence spots still stay in the cytoplasm but fluorescence intensity decreases, while red Dox fluorescence signal increases and the fluorescence spots gradually moves toward the nuclei. To more clearly show the location of Dox in subcellular compartments, the blue fluorescence from nuclei and Dox red fluorescence were simultaneously monitored for the longer time and quantitatively evaluated the degree of colocalization by calculating Pearson's correlation coefficient (Rr). As shown in Figure S47 (Supporting Information), while Dox fluorescence intensity gradually increases, the colocalization degree does substantially increases. At 12 and 16 h incubation point, Rr values reach 0.90 and 0.95, respectively, indicating that the vast majority of Dox enter the cell nuclei.

**Figure 3:**
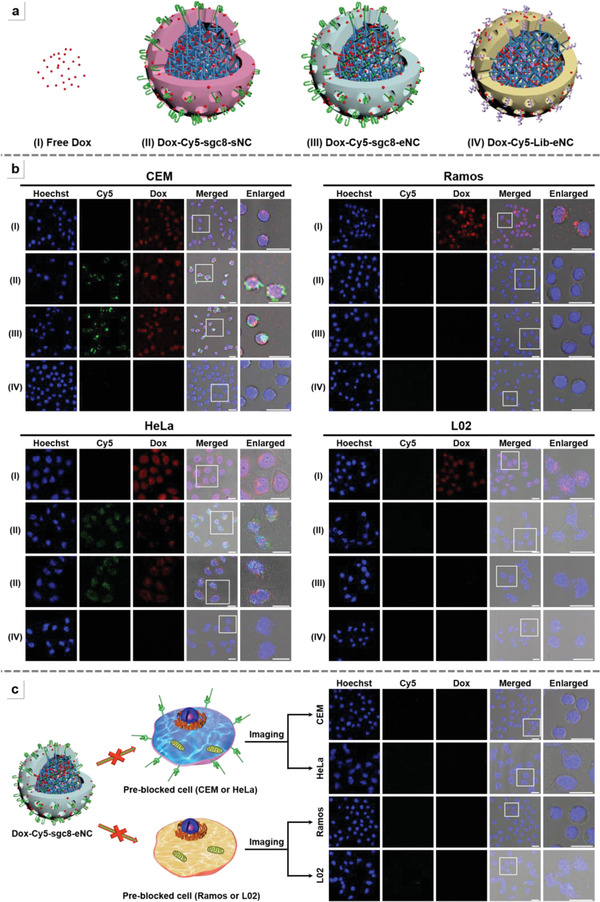
Specific cellular internalization of anticancer‐drug‐loaded sgc8‐eNC. a) The structural representation of different Dox‐formulated DNA nanomaterials, including sgc8‐eNC and its counterparts, during the assembly of which Tb“‐Cy5 was substituted for label‐free Tb″. I) free Dox; II) Dox‐Cy5‐sgc8‐sNC; III) Dox‐Cy5‐sgc8‐eNC; IV) Dox‐Cy5‐Lib‐eNC. b) The CLSM of images of Target CEM, HeLa cells, Ramos cells and normal L02 cells that were treated separately with the four Dox‐formulated nanostructures. The equivalent concentration of Dox is 2 × 10^−6^ m. c) Investigation of receptor‐dependent cellular uptake of Dox‐Cy5‐sgc8‐eNC by CLSM images. The four different cells (CEM, HeLa, Ramos, and L02) were treated with excess aptamers. Scale bar = 20 µm.

To evaluate the cell‐targeted transport of Dox, the cellular internalization efficiency of Dox‐Cy5‐sgc8‐eNC into two PTK7‐positive tumor cells (CEM and HeLa cells) and two PTK7‐negative cells (Ramos and normal L02 cells) were explored. As shown in **Figure** **3**b, when the cells were incubated with free Dox, the strong Dox fluorescence signal (red) is observed regardless of cell nature (row I), indicating that free Dox can indiscriminately diffuse across cell membrane and enter the interior of cells.^[^ ^61^, ^62^ ^]^ In contrast, even if treating the cells with Dox‐sgc8‐sNC and Dox‐sgc8‐eNC under identical conditions (row II and III), only two PTK7‐positive cells display Cy5 green fluorescence and Dox red fluorescence (panels of CEM and HeLa), while no obvious fluorescence signal is observed from the two negative cells (panels of Ramos and L02), suggesting a specific receptor‐mediated cellular internalization process. To further verify the aptamer‐specific drug transport process, an oligonucleotide with the random base sequence was used instead of e‐sgc8 for NC assembly (Dox‐Cy5‐Lib‐eNC) and cell imaging experiments were carried out according to the same procedure. The fluorescence images show that no obvious Cy5/Dox fluorescence signal is detected regardless of the expression level of surface receptor PTK7 (row IV). This is possibly because the electrostatic repulsion between negatively charged assembled DNA nanostructures and cell membranes substantially inhibits the cellular internalization process.^[^ ^63^, ^64^, ^65^ ^]^ The similar experimental phenomena are also reported by literature studies.^[^ ^15^, ^16^, ^66^, ^67^, ^68^, ^69^ ^]^ In order to validate the surface receptor‐mediated internalization of Dox‐loaded aptamer‐functionalized NC, the label‐free aptamer was used to pretreat the four cells before incubating with Dox‐sgc8‐eNC. As shown in Figure 3c, no obvious Cy5 green fluorescence and Dox red fluorescence are observed, demonstrating that membrane receptors are indispensable for cell internalization of sgc8‐eNC.

After confirming the ability of functionalized DNA nanocluster to exert cell‐targeted drug delivery and efficient drug release in tumor compartment, we used the Cell Counting Kit‐8 (CCK‐8) to investigate whether Dox‐sgc8‐eNC can effectively inhibit tumor cell proliferation and induce the unwanted damage to nontarget cells. First, the biocompatibility of sgc8‐eNC as a drug carrier was assessed by incubating Dox‐free ones with different cell lines. As described in Figure S48 (Supporting Information), even if the concentration of building blocks is more than 3100 × 10^−9^ m, the CCK‐8 assay reveals the cell viability of over 92% regardless of PTK7 expression, demonstrating that the internalization of sgc8‐eNC does not cause cell damage.^[^ ^70^ ^]^ Next, using free Dox as the control, the cytotoxicity induced by Dox‐sgc8‐eNC to different cell lines was evaluated by CCK‐8 assay where the intact cell viability without any treatment was defined as 100%. As shown in Figure S49 (Supporting Information), incubation with free Dox unambiguously causes serious cytotoxicity to CEM, HeLa, Ramos and L02 cells with little selectivity. In contrast, for Dox‐sgc8‐eNC, the cytotoxicity to target CEM and HeLa cells are dose‐dependent (Figure S49a,b, Supporting Information), while little cytotoxicity is detected to Ramos cells and L02 cells (Figure S49c,d, Supporting Information), indicating the cell specific antitumor efficacy that is determined by whether the cell surface receptor PTK7 is expressed or not. These measured data clearly demonstrate that sgc8‐eNC exhibits the desirable cell‐specific drug delivery capability, affording a promising drug nanocarrier for precise targeting‐based tumor therapy.

### 2.5 In Vivo Biodistribution of DNA Nanocluster

**Figure 4:**
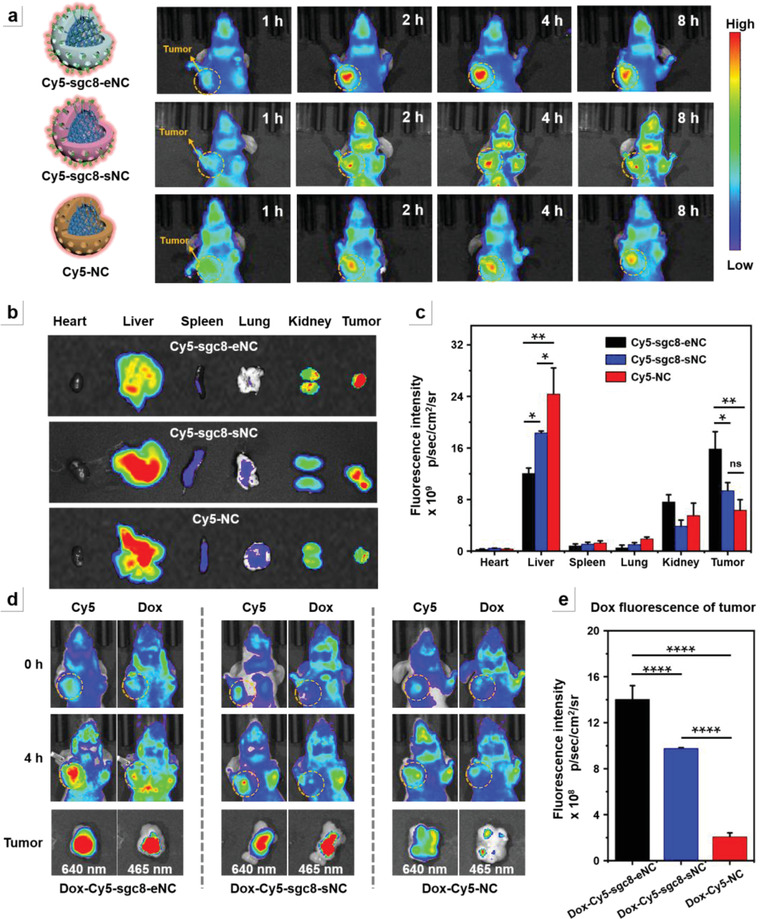
In vivo biodistribution of intravenously injected Dox‐loaded Cy5‐labeled DNA nanoclusters into HeLa tumor‐bearing mice. a) In vivo fluorescence images of mice at different time points post‐administration of nanostructures. Tumor regions are highlighted by yellow dashed circles. The three nanoparticles from top to bottom represents Cy5‐sgc8‐eNC, Cy5‐sgc8‐sNC, and Cy5‐NC, respectively. b) Distribution of different DNA nanoclusters in tumor sites and different organs detected by ex vivo imaging at 4 h post‐injection. c) Fluorescence intensity of Cy5‐labeled DNA nanoformulations located in tumor sites and different organs based on quantitative region‐of‐interest (ROI) analysis. d) In vivo fluorescence images of Dox‐loaded nanoclusters intravenously injected into tumor‐bearing animals in vivo. The bottom part represents the ex vivo images of tumor sites at 4 h administration. e) Dox fluorescence intensity of tumors surgically resected from the mice corresponding to panel d based on quantitative ROI analysis. The statistical analysis of multiple comparisons were evaluated by one‐way ANOVA. *p < 0.05, **p < 0.005, ***p < 0.0005, ****p < 0.0001, and ns represents no significant difference.

Since aptamer‐encapsulated NC nanocarriers exhibit high drug loading capacity and high tumor cell‐targeting properties, the in vivo biodistribution in tumor‐bearing mice is further studied via whole animal imaging. First, we selected healthy male BALB/c mice (15–20 g) for the construction of HeLa tumor‐bearing model mice by subcutaneous inoculation. The blood half‐life of DNA nanoclusters in healthy BALB/c nude mice was evaluated according to a literature method.^[^ ^49^, ^71^, ^72^, ^73^ ^]^ Figure S50 (Supporting Information) shows that the blood half‐life of sgc8‐eNC is improved compared with two counterparts (NC and sgc8‐sNC) and is much longer than e‐sgc8 and previously‐reported DNA tetrahedral objects.^[^ ^71^, ^72^ ^]^ Then, when the tumor volume reaches about 400 mm^3^, Cy5‐labeled DNA nanoclusters (Cy5‐sgc8‐eNC, Cy5‐sgc8‐sNC, and Cy5‐NC) were separately administrated into tumor‐bearing xenograft murine mice via tail vein injection. The Cy5 fluorescence signal was measured to evaluate the in vivo biodistribution of the three nanoformulations. As shown in **Figure** **4**a, it can be clearly observed that Cy5‐sgc8‐eNC nanostructures gradually accumulate at the tumor sites and reach the highest value at 4 h post‐administration. Even at 8 h, a considerable amount of Cy5‐sgc8‐eNC is still observed from the tumor sites. Although Cy5‐sgc8‐sNC displays a certain degree of tumor accumulation, the in vivo fluorescence images show a weak signal over the entire 8‐h duration. This is because the enzymatic degradation‐induced depletion of surface‐hybridized aptamers cannot get the compensation due to the lack of targeting ligands in the internal cavity and thus a large portion of nanostructures lose the ability to target cancerous tissues once encountering biological degradation during circulating in the bloodstream. For nontargeted Cy5‐NC, the fluorescence signal is also detected at tumor site possibly due to the well‐known enhanced permeability and retention (EPR) effect, but the signal is much weaker. This is because the passive targeting mechanism can only make a very limited fraction of administered nanocarriers reside in tumors.^[^ ^74^ ^]^ Next, in order to observe the distribution of nanomaterials in different tissues and tumor more accurately, after the mice were anesthetized and dislocated at 4 h post‐injection of DNA NCs, the main organs of mice (heart, liver, spleen, lungs and kidney) and tumor tissues were collected for the fluorescence imaging. As shown in Figure 4b, for Cy5‐NC and Cy5‐sgc8‐sNC, the highest fluorescence signal appears in the liver, indicating that the liver is where NC nanostructures are easiest to accumulate. This is reasonable since nanomaterials can be rapidly uptake by liver and encounter the degradation and elimination.^[^ ^75^ ^]^ In contrast to Cy5‐NC and Cy5‐sgc8‐sNC, Cy5‐sgc8‐eNC has a significantly enhanced fluorescence signal in tumor tissue, while the fluorescence intensity in liver is substantially reduced, suggesting the desirable accumulation of sgc8‐eNC formulations. The corresponding quantitative analyses of nanomaterials located in the several tissues are presented in Figure 4c. If the fluorescence signal from tumor and liver (abbreviated as F_tumor_ and F_liver_, respectively) is recorded to quantitatively evaluate the tumor targeting and F_tumor_/F_liver_ is used to evaluate the tumor accumulation, the accumulation efficiency of Cy5‐sgc8‐eNC in tumor site is 2.6 times and 5.1 times higher than Cy5‐sgc8‐sNC and Cy5‐NC, respectively.

In order to explore the potential of sgc8‐eNC as a drug delivery system, another fifteen mice bearing HeLa tumor were randomly divided into 3 groups (five per group) and then intravenously injected with different Dox‐loaded formulations, including Dox‐Cy5‐sgc8‐eNC, Dox‐Cy5‐sgc8‐sNC, and Dox‐Cy5‐NC. At 4 h post‐injection, the two‐color fluorescence (Cy5 and Dox) imaging was performed to evaluate the targeting delivery efficiency of Dox. As shown in the middle panel of Figure 4d, for Dox‐Cy5‐sgc8‐eNC, the strong fluorescence signal is observed at the tumor site, and the tumor fluorescence intensity of Dox is higher than Dox‐Cy5‐sgc8‐sNC and Dox‐Cy5‐NC. In order to intuitively compare the amount of Dox delivered into tumor sites by different carriers, the mice were dislocated for collecting tumor tissues and ex vivo fluorescence imaging. As shown in the lower panel of Figure 4d, Dox‐sgc8‐eNC group does exhibit the strongest fluorescence signal at the tumor site in terms of both Cy5 and Dox. Meanwhile, the Dox fluorescence intensity recorded from tumor tissue was also quantitatively analyzed (Figure 4e). It is found that the signal from Dox‐sgc8‐eNC group is 1.4 times and 6.8 times higher than Dox‐sgc8‐sNC and Dox‐NC groups, respectively. The experimental results demonstrate that sgc8‐eNC is endonuclease‐resistant enough to efficiently transport Dox drugs into tumor site and thus suitable for high‐efficiency tumor‐targeted drug delivery in vivo. Worthy of mention is that, taking into account a fact that Dox‐sgc8‐sNC and Dox‐NC can be degraded and not fluoresce (similar to Figure S26 in the Supporting Information), the data were measured at 4‐h post‐administration. Naturally, the tumor accumulation of Dox‐sgc8‐eNC can increase with the increment of incubation time after systematic administration, leading to the more sharp contrast in tumor accumulation to the counterparts.

### 2.6 Inhibition of Tumor Growth in Vivo by Dox‐sgc8‐eNC

**Figure 5:**
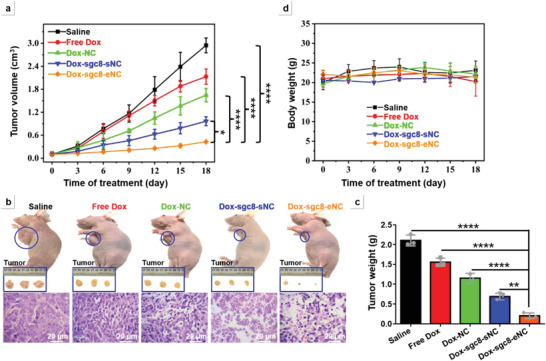
Inhibition of tumor growth of Dox‐loaded DNA Nanoclusters in HeLa tumor‐bearing BALB/c nude mouse model (male). a) Tumor volume curve of treatment groups systemically administrated separately with saline, free Dox, Dox‐NC, Dox‐sgc8‐sNC, and Dox‐sgc8‐eNC. b) Photos of tumor‐bearing mice subjected to different treatments and corresponding histological analysis of dissected tumor tissues by hematoxylin and eosin (H&E) staining. Scale bar = 20 µm. Inset: photos of dissected tumors. c) Tumor weights after 18 days of treatment harvested from various treatment groups. The measured data are expressed as the means ± SD. The statistical analysis of multiple comparisons were evaluated by one‐way ANOVA. **p < 0.005, ***p < 0.0005, ****p < 0.0001. d) Time‐dependent body weight changes of tumor‐bearing mice intravenously injected with various Dox‐loaded DNA nanoclusters.

The in vivo treatment efficacy of anticancer chemotherapeutics drugs (Dox) delivered by sgc8‐eNC was explored using a HeLa tumor‐bearing BALB/c nude mouse model. For this, sgc8‐eNC, Free Dox, Dox‐NC, and Dox‐sgc8‐sNC and Dox‐sgc8‐eNC were separately injected into the mice bearing tumors through tail vein every 3 days for 18 consecutive days, and Dox dose is equivalent to 2 mg kg^−1^. Another group of tumor‐bearing nude mice was injected with normal saline alone as control. Before each treatment, tumor volume and body weight of each mouse were measured and assessed as a function of time during the whole treatment process. **Figure** **5**a shows the dynamic change of tumor volume in nude mouse models. It can be found that the tumor growth rate without any drug treatment (Saline) is the fastest, reaching ≈3000 mm^3^ at the 18th day post‐systemic administration. The treatment with free Dox only induces a slight decrease in tumor growth rate, which is because Dox molecules without cell targeting ability (Figure S46, Supporting Information) could robustly enter almost the nontarget organs and tissues and lead to the extremely insufficient amount of anticancer drugs in tumor site. Due to the EPR effect of nanocarriers in cancerous tumors, Dox‐NC exhibits the enhanced ability to delay tumor growth. Nevertheless, during in vivo circulation, due to the lack of tumor‐targeting ligands, Dox‐NCs are uncontrollably and systemically distributed throughout the body and inevitably encounter the endonuclease‐mediated degradation and clearance, dampening the inherent therapeutic effects of chemotherapeutics to a considerable extent. When endowed with specific targeting ability by equipping with sgc8 aptamer, the corresponding formulation, Dox‐sgc8‐sNC, can actively transport anticancer cargoes in a specific fashion and lead to the increase in local concentration of chemotherapeutic agents in tumor tissues, further improving the antitumor efficacy. Strikingly, compared with Saline and the other two Dox formulations, Dox‐sgc8‐eNC possesses the strongest tumor suppressive effects. As such, no obvious tumor growth is detected at 18 d post‐treatment and the tumor volume is 8.7‐fold smaller than normal saline group and 2.6‐fold smaller than the representative DNA nanosphere vehicle (Dox‐sgc8‐sNC) with surface‐confined targeting ligands. This should be attributed to its inherent feature that the long‐lasting tumor targeting ability can be maintained during the circulation in the bloodstream of mice due to the outward movement of sgc8 molecules from the internal reserve pool. Thus, Dox‐ sgc8‐eNC has more opportunities to search the areas it passes through for tumor cell membrane receptors and efficiently enter tumor cells via receptor‐mediated endocytosis, leading to the high accumulation of chemotherapeutics in cancerous tissues.

Collectively, upon the treatment with Dox‐loaded formulations, the tumor growth rate is inhibited and decreases in the order of free Dox > Dox‐NC > Dox‐sgc8‐sNC > Dox‐sgc8‐eNC, which is consistent with the representative photographs of whole mice and dissected tumors (Figure 5b). The measured data on tumor weight are shown in Figure 5c. Moreover, the histological analysis of tumor tissue sections by hematoxylin and eosin (H&E) staining was also used to evaluate the tumor suppression efficacy upon the treatment with different Dox‐loaded formulations. It can be seen from the lower panel of Figure 5b that tumor slices of mice treated with Free Dox and Dox‐NC do not display substantial change in histological morphology compared with Saline group, indicating that the two groups of anticancer materials have no obvious tumor growth inhibitor effect. Although Dox‐sgc8‐sNC caused the remarkable alteration in histological morphology, but the morphological change induced by Dox‐sgc8‐eNC, such as cytoplasm shrinkage and nucleus condensation, is more obvious, indicating the typical apoptotic cell death.^[^ ^49^, ^76^, ^77^ ^]^ It is worth noting that the body weight of Dox‐sgc8‐eNC‐treated mice did not significantly decrease during the treatment period (Figure 5d), and the survival rate of mice was 100%, demonstrating that Dox‐sgc8‐eNC has no apparent systemic toxicity and is safe and effective. The biological safety of Dox‐sgc8‐eNC was also confirmed by examining the pathological changes of main organs by H&E staining (Figure S51, Supporting Information). In addition, because the majority of sgc8‐eNCs specifically recognize and accumulate in tumor tissues and the rest are uptake mainly by the liver and kidney (Figures 4b,c), the liver toxicity and kidney toxicity were examined as presented in Figure S52 (Supporting Information). One can notice that the treatment with Dox‐sgc8‐eNC does cause the detectable damage to liver and kidney (Figure S52a, Supporting Information). Moreover, there is no significant difference in the serum level of alanine transferase (ALT), aspartate aminotransferase (AST), creatinine (CRE) and blood urea nitrogen (BUN) between Saline and Dox‐sgc8‐eNC, implying that Dox‐sgc8‐eNC does not lead to the liver toxicity and kidney toxicity.^[^ ^78^, ^79^ ^]^

Moreover, Dox‐sgc8‐eNC possesses an equivalent therapeutic efficacy in female HeLa tumor‐bearing BALB/c nude mouse model (Figure S53, Supporting Information). Possibly, the tumor growth/suppression behavior is independent of mouse estrogen for subcutaneous xenograft model of HeLa cells. ^[^ ^80^ ^]^

## 3 Conclusion

In summary, by a stepwise hierarchical self‐assembly technique, a versatile DNA nanostructure, sgc8‐eNC, is demonstrated as a powerful drug delivery carrier with apparent advantages, such as the persistent active targeting functionality, extremely high cargo capacity, substantially enhanced stability in blood circulation, good biocompatibility and fine assembly controllability. The attractive functions of sgc8‐eNC suitable for targeted cancer therapy are based on its two unique structural designs. i) Doubly rigidified structures. First, the basic unit is DNA Tetra that possesses unique structural features, such as 3D rigid structure, many binding sites for intercalative chemotherapeutic agents and enhanced resistance to enzymatic attack. Secondly, the enzymatic ligation of crosslinking interaction between the four connection arms on the bottom face of Tetra unit promotes the formation of a 3D interwoven rigidified DNA backbone. The two factors ensure the rigid structure of sgc8‐eNC. ii) Enriching sgc8‐eNC with cell‐targeting ligands. The sgc8 is an aptamer capable of binding to a particular transmembrane protein PTK7 overexpressed in many tumor cells (e.g., CEM and HeLa cells) and installed at the vertex of DNA Tetra unit. Thus, besides the intrinsic affinity of original aptamer is maintained, the aptamers can be arranged both on the surface and in the internal cavity (forming an internal reserve pool). Even if the surface‐hybridized aptamers are degraded, the ones in the reserved pool can move outward to supplement the consumed targeting.

On the basis of unique structural features, sgc8‐eNC exhibits several attractive advantages for targeted drug delivery. a) A long systemic circulation time. The sgc8‐eNC can retain its structural integrity for 8 h in a serum solution or in in vivo setting. b) Extremely high cargo loading capability. Sgc8‐eNC consisting of 283 Tetra units has enough binding sites to accommodate considerable amounts of therapeutic drugs. The ratio of Dox‐to‐carrier is up to 5670:1, and the loaded drugs can be protected from early leakage at physiological pH. c) Persistent tumor targeting in a complex environment. The Dox‐loaded sgc8‐eNC specifically recognizes PTK7‐positive tumor cells and exclusively internalized into tumor tissues via receptor‐guided endocytosis. Moreover, the tumor‐tissue targeting activity can be maintained during the blood circulation, eliminating the harmful off‐target systemic side effects toward healthy tissues or organs. d) Good biocompatibility with living organisms. Even at very high concentration of DNA building components (e.g., over 3100 × 10^−9^ m), sgc8‐eNC displays no apparent toxicity toward living cell lines at the cellular level, including target and nontarget cells. After densely loading chemotherapeutics, there is also no obvious cytotoxicity or adverse effects against healthy tissues and normal organs at the system level. e) Good universality due to the remarkable sequence programmability. By changing the base sequence of targeting domain, the aptamer‐encapsulated NC is easily designed to recognize other cell membrane receptors and expand its applicability to the targeted therapeutics of other cancers. f) Simple assembly process and high assembly yield. Although one hierarchically assembled object contains nearly three hundred structural units each of which has seven DNA building blocks, the assembly process is very simple where only several mixing steps are involved. Moreover, the assembly yield is almost 100% and no detectable residual building blocks are observed by gel electrophoresis. Significantly, the experimental data from the characterization of nuclease degradation resistance, payload capacity and molecular mechanism for in vivo tumor targeting, especially from in vivo targeted drug delivery to tumor tissues and therapeutic potency in HeLa tumor‐bearing xenograft mouse model, show that sgc8‐eNC is a useful tool for targeted tumor therapy via systemic drug delivery. More importantly, the design of doubly rigidified structures and built‐in reserve pool of transferable aptamers provide important insights into the deliberate design of sophisticated DNA nanostructure vehicles with nanoscale precision to realize the elegant combination of unique features, such as the mechanical rigidity‐enhanced nuclease degradation resistance and automatic adjustment of the spatial location of recognition elements to maintain the collective targeting activity, finally meeting the requirements of specialized applications in biomedical fields.

## 4 Experimental Section

### Stepwise Assembly of DNA Nanocluster

DNA sequences, including Ta, Tb, Tc, Ta′, Tb′, Tc′, and LS, were first phosphorylated using T4 PNK. The corresponding experimental steps are described as follows: a certain amount of 1 × TE buffer was added to dissolve the DNA powder, followed by adding an appropriate amount of ATP (final concentration, 1 × 10^−3^ m), T4 PNK (final concentration, 0.1 U µL^−1^) and 10 × T4 PNK buffer (700 × 10^−3^ m Tris–HCl, 100 × 10^−3^ m MgCl_2_, 50 × 10^−3^ m DTT, pH 7.6, the final concentration is 1 ×). The final concentration of DNA strand was 10 × 10^−6^ m, and the resulting solution was incubated at 37 °C for 4 h. After purification by polyacrylamide gel electrophoresis (PAGE), the concentration of phosphorylated DNA strand was adjusted to 10 × 10^−6^ m and stored at 4 °C for further use.

Assembly of DNA nanocluster (NC) was conducted in 25 µL of 1 × T4 DNA ligase buffer according to the following procedure: Step ①, Tetra assembly. A 2.5 µL aliquot of 10 × T4 DNA ligase buffer (400 × 10^−3^ m Tris–HCL, 100 × 10^−3^ m MgCl_2_, 100 × 10^−3^ m DTT, 5 × 10^−3^ m ATP, pH = 7.8) and equal amounts (0.5 µL, 10 × 10^−6^ m) of Ta, Tb, Tc, Ta′, Tb′, Tc′, and LS were added into 18.5 µL of H_2_O. After heating at 90 °C for 5 min, the mixture was placed in a dry bath incubator and gradually cooled down to room temperature to ensure the full assembly of DNA building blocks. The concentration of DNA tetrahedron unit was about 200 × 10^−9^ m. Step ②, Ligation‐based rigidification. Subsequently, T4 DNA ligase (0.5 µL, 400 U µL^−1^) was added and incubated at 16 °C overnight to form the DNA nanocluster (NC). Step ③, Thermal inactivation. The assembly solution was deactivated at 65 °C for 10 min to terminate the activity of ligase. Because the finally‐assembled NC consists of DNA tetrahedron units without the Apt‐binding site at vertex‐iv, it is called bare basic NC (b‐NC). The corresponding tetrahedral unit is called b‐Tetra.

For the sgc8‐eNC assemblies, DNA tetrahedra were firstly assembled according to the step ①, but e‐Tc was used instead of Tc. Then, e‐sgc8 (0.5 µL, 10 × 10^−6^ m) was added and incubated at 37 °C for 2 h to ensure the installation of e‐sgc8 onto the vertex iv of tetrahedron where the molar ratio of sgc8‐to‐tetrahedron is 1:1. The assembled product is called sgc8‐Tetra. After undergoing ligation‐based solidification (step ②) and thermal inactivation (step ③), sgc8‐eNC was obtained. One as‐assembled sgc8‐eNC has 283 sgc8‐Tetra units (seen in Scheme S4 of the Supporting Information), each structural unit has one set of building blocks at an equal molar ratio and the concentration of each building block in the assembly solution is 200 × 10^−9^ m. Thus, the sgc8‐eNC concentration is estimated to be 707 × 10^−12^ m. For sgc8‐eNC, both the surface and internal cavity are enriched with e‐sgc8 aptamers.

For aptamer‐sheathed nanocluster (sgc8‐sNC), the assembly was performed according to the described‐above procedure (steps ①–③) with slight modification. Namely, e‐sgc8 was added at the final stage. Specifically, the DNA tetrahedron with an Apt‐binding site at the vertex‐iv was assembled at step ①. After enzymatic ligation (step ②) and thermal inactivation (step ③), e‐sgc8 (0.5 µL, 5 × 10^−6^ m) with a fragment complementary to the Apt‐binding site of Tetra was added and incubated at 37 °C for 2 h, forming sgc8‐sheathed DNA nanostructure (sgc8‐sNC) only on whose surface aptamers are spiked.

### Gel Electrophoresis

To prepare native polyacrylamide gel (nPAGE, 6%), gel stock solution (29:1 acrylamide:bisacrylamide mixture, 30% (w/v), 1.2 mL), 5 × TBE buffer (pH = 7.9, 450 × 10^−3^ m Tris–HCl, 450 × 10^−3^ m boric Acid, 10 × 10^−3^ m EDTA) (1.2 mL), APS (10% w/V, 100 µL), TEMED (5 µL), and ultrapure water (3.6 mL) were mixed and poured into the supporting frame (6 mL), followed by immediately inserting the appropriate comb. The gel solution was allowed to polymerize at room temperature for 15 min. For gel characterization, a sample (8 µL) was mixed with 2 µL of 10 × SYBR Green I and 2 µL of 6 × Loading buffer and incubated at room temperature for 5 min. The sample was loaded into the well of preformed gel. The nPAGE was run at 80 V for 60 min in a 0.5 × TBE buffer on a gel electrophoresis instrument (Bio‐RAD, USA). Finally, the gel image was photographed on a ChemiDoc XRS Imaging system with the image acquisition and analysis software Image Lab (Bio‐RAD, USA).

### AFM Characterization of DNA Nanocluster (NC)

A 5 µL aliquot of DNA NC sample was diluted to 50 µL with 1 × T4 DNA ligase buffer, 10 µL of which was deposited onto freshly cleaved mica. After 20 min incubation at room temperature, the mica surface was washed 3 times with ultrapure water (100 µL) and dried with pure nitrogen. AFM Measurement was performed at a scan rate of 2 Hz under air atmosphere using tapping mode on a MultiMode 8 atomic force microscope (Bruker, Germany). The AFM image was analyzed by the Nanoscope analysis 1.7 software.

### Dynamic Light Scattering Measurement

The sample of interest was diluted 10 times with 1 × T4 DNA ligase buffer, and the final volume was 1 mL. Dynamic light scattering (DLS) measurement was performed using the Non‐Invasive‐Back‐Scatter (NIBS) mode on Nano ZS Zetasizer (Malvern Instruments Ltd., England) equipped with a 4.0 mW laser operating at a wavelength of 633 nm and a scattering angle of 173°. Each sample was measured in triplicate.

### Evaluation of Serum Stability

The serum stability of sgc8‐eNC was evaluated by comparing with sgc8‐sNC, sgc8‐NW, sgc8‐Tetra and intact sgc8. The Fe‐sgc8 modified with FAM fluorophore was used instead of e‐sgc8 for constructing different DNA nanostructures. First, fluorescently modified sgc8‐NW (F‐sgc8‐NW) was prepared as described in Figure S22 (Supporting Information), while F‐sgc8‐sNC, F‐sgc8‐eNC and F‐sgc8‐Tetra were constructed according to the procedure adopted in the section of “Stepwise assembly of DNA nanocluster.” Then, 10% fresh mouse serum solution of DNA nanostructure was prepared by mixing 25 µL of DNA nanostructure with 3 µL of fresh mouse serum and 2 µL of 1 × T4 buffer. The resulting solution was incubated at 37 °C for different time periods (0, 0.5, 1, 2, 4, 6, or 8 h). Subsequently, the reaction solution was kept at −20 °C to terminate the enzymatic degradation until use. To characterize the residual DNA strands, 6% nPAGE analysis was performed at 80 V in 0.5 × TBE buffer.

### Outward Movement of Aptamers in the Cavity of sgc8‐eNC Toward the Surface

The sgc8‐eNC was assembled as described in the section “Stepwise Assembly of DNA nanocluster,” but Tb'‐FAM was used instead of Tb'. Then, Ce‐sgc8 (200 × 10^−9^ m) was added to FAM‐sgc8‐eNC solution and kept at room temperature for 0.5 h, resulting in the displacement reaction solution containing sgc8‐eNC‐i. Afterward, an Amicon Ultra centrifuge tube (MWCO‐50K, Merck Millipore, USA) was used to remove the displaced e‐sgc8 and excess Ce‐sgc8 according to the following procedure: First, 100 µL of 1 × T4 DNA Ligase buffer was used to wet the ultrafiltration tube and removed by centrifugation at 4000 RPM for 5 min. Then, the reaction solution (100 µL) was added into the ultrafiltration tube and centrifuged at 10 000 RPM for 5 min. The sgc8‐eNC‐i was washed twice with 1 × T4 DNA Ligase buffer (50 µL for each washing). Afterward, added 50 µL of 1 × T4 DNA Ligase buffer, carefully rinsed the filter membrane with a pipette and inverted the ultrafiltration tube, followed by centrifugation (10 000 RPM, 5 min). This process was repeated once, and the resulting residue was dissolved in 1 × T4 DNA Ligase buffer, obtaining the purified sgc8‐eNC‐i (100 µL).

The sgc8‐eNC‐i was kept at room temperature for different time intervals (0, 1, 2, 4, or 6 h). Then, 25 µL of sgc8‐eNC‐i was mixed with 175 µL of culture medium and incubated with HeLa cells (5 × 10^4^ cells) in a humidified atmosphere of 5% CO_2_ at 37 °C for 2 h. After the culture medium was removed, the cells were washed twice with PBS and fixed with 4% paraformaldehyde for 15 min, followed by staining with Hoechst 33342 (10 µg mL^−1^) for 5 min. The resulting cells were washed twice by PBS again to remove Hoechst staining solution. Next, 2 µL of Antifade Mounting Medium was added dropwise onto the center of cover slip and placed upside down on a microscope slide. The fluorescence images were taken on a Leica SP8 laser scanning confocal microscope (CLSM, Leica, Germany). Hoechst 33342 was excited at a wavelength of 405 nm, while FAM was excited at 488 nm.

### Cell‐Targeted Transport of Dox‐sgc8‐eNC

Different types of cells were separately incubated with Dox‐sgc8‐eNC and its counterpart nanostructures (Dox‐sgc8‐sNC and Dox‐Lib‐eNC) for 4 h in a humidified atmosphere of 5% CO_2_ at 37 °C. Equivalent amount of Dox was employed in each experimental group. Then, the resulting cells were washed twice with PBS buffer and stained with 10 µg mL^−1^ Hoechst 33342 for 5 min. For adherent cells, 2 µL of Antifade Mounting Medium was added dropwise onto the center of cover slip and placed upside down on a microscope slide. For suspension cells, the cell sample (10 µL) was dropped into a confocal dish. The fluorescence imaging was performed.

To preblock the receptor binding sites on cancer cells, the cells (CEM, HeLa, Ramos and L02) were treated with excess sgc8 aptamer (5 µL, 10 × 10^−6^ m) by incubation in a humidified atmosphere of 5% CO_2_ at 37 °C for 1 h, followed by washing twice with PBS. The incubation with sgc8‐eNC was performed as described above.

### In Vivo Imaging and Biodistribution Analysis

When tumor volume reached 400 mm^3^, the mice were divided into three groups (at least 4 mice per group) randomly. Cy5‐sgc8‐eNC (500 × 10^−9^ m calculated by Cy5‐labeled Tb′; 100 µL) and two controls (Cy5‐labeled sgc8‐sNC and Cy5‐labeled NC) were separately administrated into the bodies of mice by tail‐vein injection. The whole animal imaging was performed at 1, 2, 4, and 8 h post‐administration on IVIS Lumina LT Series III Imaging Spectrum System (excitation at 640 nm filter) (PerkinElmer, USA). A specifically designed group of mice were sacrificed at 4 h post‐administration to collect the tumor and major organs (heart, liver, spleen, lungs, and kidney) for ex vivo fluorescence imaging. The fluorescence intensity was estimated by IVIS Lumina LT Series III Imaging Spectrum System software.

For the evaluation of tumor accumulation of Dox‐loaded Cy5‐labeled DNA nanoclusters, the fluorescence signals of Dox and Cy5 were analyzed by in vivo imaging. First, the Dox‐loaded Cy5‐sgc8‐eNC (500 × 10^−9^ m estimated from the concentration of Cy5‐labeled Tb′; 100 µL) and two control formulations (Dox‐Cy5‐sgc8‐sNC and Dox‐Cy5‐NC at an equivalent concentration of Dox, 10 × 10^−6^ m) were separately administrated into the bodies of mice by tail‐vein injection. The whole animal imaging was performed at 0 and 4 h post‐administration (excitation at 465 nm for Dox and 640 nm for Cy5). The mice were sacrificed at 4 h post‐administration, and the tumors were collected for ex vivo fluorescence imaging. The fluorescence intensity was estimated by IVIS Lumina LT Series III Imaging Spectrum System software.

### In Vivo Tumor Suppression Study

When tumor volume reached 100 mm^3^, the mice were divided into five groups (at least 4 mice per group) randomly, followed by systematical administration‐based chemotherapy. Namely, Dox‐sgc8‐eNC and the controls (saline, free Dox, Dox‐NC and Dox‐sgc8‐sNC) were separately administrated into the bodies of mice by tail vein injection every three days, and the equivalent concentration of Dox is 2 mg kg^−1^. Tumor size and body weight were measured before the next administration of drug formulations, while tumor volume (*V*) was calculated based on the following equation: *V* = (length × width^2^)/2. All the mice were sacrificed on 18‐day post‐treatment and harvested the tumors and organs for histological examination and ex vivo photographing tumors.

### Statistical Analysis

Unless otherwise stated, all measurements were performed in triplicate and the data are presented as mean ± standard deviation (SD). The sample size (*n*) for each statistical analysis is shown in the figure caption. GraphPad Prism 8 was used for the statistical analysis in this research. For the size analyses, the data was preprocessed by the logarithmic function transformation via Origin software as needed. The statistical significance was determined using a two‐tailed unpaired *t* test. For multiple comparisons, the data were analyzed using a one‐way analysis of variance (ANOVA). Samples that were > 3 SD from the mean were excluded as outliers. For all tests, the asterisks indicate *p* values *p < 0.05, **p < 0.005, ***p < 0.0005, ****p < 0.0001, and ns represents no significant difference. *p* < 0.05 was considered to have the statistical significance.

## Conflict of Interest

The authors declare no conflict of interest.

## Supporting information

Supporting Information

## Data Availability

The data that support the findings of this study are available in the supplementary material of this article.
