# Supplementary material for: Outward Movement of Targeting Ligands from a Built‐In Reserve Pool in Nuclease‐Resistant 3D Hierarchical DNA Nanocluster for in Vivo High‐Precision Cancer Therapy
Source: Adv Sci (Weinh). 2022 Oct 17;9(33):2203698. doi: 10.1002/advs.202203698 (PMC9685459; doi:10.1002/advs.202203698)
Supplement: Supplementary file 1 — Supporting Information [file ADVS-9-2203698-s001.pdf]

Supporting Information

Outward movement of targeting ligands from a built-in reserve pool in nuclease-resistant 3D hierarchical DNA nanocluster for in vivo high-precision cancer therapy

Weijun Wang[a,1], *Yansha Gao*[a,1], *Yaxin Chen*[a], *Wenqing Wang*[a], *Qian Li*[a], *Zhiyi Huang*[a], *Jingjing Zhang*[a], *Qi Xiang*[a,b], *Zai-Sheng Wu*[a,]*

Table of Contents

Section A: Supplementary Experimental Procedures

SA.1 Materials
SA.2 Cell culture
SA.3 Optimization of aptamer concentration for the assembly of sgc8-sNC materials
SA.4 Specific recognition of target cancer cells
SA.5 Investigation of endocytosis pathway
SA.6 Colocalization study
SA.7 Drug Loading
SA.8 Cytotoxicity analysis of DNA assembled nanostructures
SA.9 *In vivo* mouse xenograft model
SA.10 Histopathological analysis by hematoxylin and eosin (H&E) staining
SA.11 Si-DLBM construction
SA.12 Dual-color fluorescence imaging of Si-DLBM

Section B: Supplementary Discussion

SB.1 Characterization of DNA nanocluster
SB.2 Unique serum stability of aptamer-embedded DNA nanoclusters
SB.3 Internalization pathway of DNA nanocluster

Section C: Supplementary Table

Table S1. Sequences of oligonucleotides designed in this work

Section D: Supplementary Results and Discussion

Scheme S1. Schematic drawing of DNA tetrahedron unit, accompanied by the base sequence of DNA components and functional domains
Scheme S2. Three-dimensional cartoon view to show the structure of sgc8-eNC and its molecular mechanism for the maintenance of desirable specific cancer-cell recognition even if encountering the enzymatic digestion
Scheme S3. Schematic illustration of the assembly of aptamer surface-scattered nanocluster (sgc8-sNC)
Scheme S4. Estimating the number of sgc8-Tetra unit in one sgc8-eNC particle
Figure S1. Characterization of basic NC consisting of DNA tetrahedra
Figure S2. Influence of the concentration of building blocks on the assembly of DNA NC
Figure S3. The dependence of melting behavior of different double-stranded (ds) fragments on the content of G/C base pairs (GC content)
Figure S4. The relationship between DNA tetrahedron morphology and GC content in the sticky ends
Figure S5. Structural morphology of ligated nanostructures assembled from DNA tetrahedra with the sticky ends of different GC contents
Figure S6. Assembly of DNA NC from tetrahedron unit with two mutant bases (red) in the sticky end-ii and/or sticky end-iii
Figure S7. AFM characterization of three DNA assemblies with two-base-mutated sticky ends
Figure S8. Assembly of mutant DNA NCs from tetrahedron unit with seven mutant bases (in red) in the sticky end-ii and/or sticky end-iii
Figure S9. AFM characterization of three DNA assemblies with seven-base-mutated sticky ends

Figure S10. Assembly of DNA NCs from tetrahedron unit without the sticky end-ii and/or sticky end-iii
Figure S11. AFM characterization of DNA NCs assembled from tetrahedron unit without the sticky end-ii and/or sticky end-iii
Figure S12. DLS size and thermodynamic stability of several mutant DNA NC variants
Figure S13. The dependence of DNA NC on the 3D structure of DNA tetrahedron unit
Figure S14. The assembly of DNA nanostructure consisting of common DNA triangle unit that is also called hollow DNA triangle without Y-shaped backbone in the center
Figure S15. AFM and DLS characterization of p-NC and h-NC
Figure S16. The effect of base number in sticky ends on the assembly of nanoclusters
Figure S17. Characterization of DNA tetrahedron unit
Figure S18. Characterization of DNA tetrahedron unit hybridized with sgc8 aptamer (sgc8-Tetra)
Figure S19. Characterization of aptamer-sheathed nanocluster, sgc8-sNC
Figure S20. Specific recognition of sgc8-eNC toward target cancer cells
Figure S21. No cross-binding of sgc8-eNC to normal cells
Figure S22. The universality of aptamer-conjugated DNA nanoclusters for specific recognition of cancerous cells
Figure S23. Serum stability of DNA NCs
Figure S24. Cellular internalization of aptamer-conjugated DNA nanomaterials pre-subjected to the serum degradation into target HeLa cells
Figure S25. Serum stability analysis of cell-targeting ability of aptamer-conjugated DNA NC evaluated by dual-color fluorescence imaging at the cellular level
Figure S26. Serum stability analysis of cell-targeting ability of aptamer-conjugated DNA NC by whole-animal *in vivo* imaging of tumor-bearing mice
Figure S27. Single-color fluorescence imaging to validate the transfer of aptamers between different DNA NC nanostructures
Figure S28. Dual-color fluorescence imaging to validate the transfer of aptamers between different DNA NC nanostructures
Figure S29. Validation of the hydrophobic interactions between Chol-NC and Si-DLBM particles with the help of dye-stained Si-DLBM
Figure S30. Validation of aptamer transfer between different DNA NCs at the cellular level by dual-color fluorescence imaging
Figure S31. Validation of aptamers transfer between different DNA NCs by label-free aptamer-mediated cellular internalization
Figure S32 Validation of aptamer transfer out from F-sgc8-eNC by measuring the decrease in the fluorescence intensity of internalized nanostructures *via* CLSM technique
Figure S33. Free energy change for the secondary structure of aptamer-incorporated DNA duplex
Figure S34. Peeling off the surface-hybridized aptamers from sgc8-eNC by competitive hybridization to its complementary probe
Figure S35. Time-dependent hybridization competition of Ce-sgc8 for e-sgc8 with DNA NC
Figure S36. The influence of incubation time and Ce-sgc8 concentration on the hybridization competition with DNA NC for e-sgc8
Figure S37. Possible mechanism for peeling off surface-hybridized aptamers from sgc8-sNC
Figure S38. The endocytosis pathway of sgc8-eNC and endosomal escape properties
Figure S39. General totoxicity of the inhibitors of endocytic pathways toward HeLa cells
Figure S40. The standard linear calibration curve of Doxorubicin (Dox) fluorescence versus its concentration
Figure S41. Drug payload capacity of different DNA assemblies
Figure S42. AFM characterization of Dox-sgc8-eNC

Figure S43. The stability of Dox-sgc8-eNC formulation
Figure S44. *In vitro* cumulative release of Dox from Dox-sgc8-eNC in harsh biological conditions
Figure S45. The universality of sgc8-eNC as an anticancer drug nanocarrier
Figure S46. Schematic diagram of targeted transport of anticancer drugs by Sgc8-eNC into the nuclei
Figure S47. Fluorescence colocalization analysis to evaluate drug accumulation in the nuclei
Figure S48. General toxicity of DNA NC nanostructures to different cells
Figure S49. Cytotoxicity profiles of free Dox and Dox-sgc8-eNC measured by CCK-8 to assess the targeted therapeutic effect
Figure S50. The *in vivo* half-life time of DNA NCs
Figure S51. Histological analysis of hematoxylin and eosin (H&E) stained slices of major organs harvested from tumor-bearing mice administrated with different Dox-loaded DNA formulations
Figure S52. The liver and kidney toxicity of tumor-bearing mice caused during Dox-sgc8-eNC-based treatment
Figure S53. Inhibition of tumor growth of Dox-sgc8-eNC in HeLa tumor-bearing BALB/c nude mouse model (female)

Section E: Supplementary References

Section A: Supplementary Procedures

SA.1 Materials

All DNA oligonucleotides designed in this study were synthesized and purified by Sangon Biotech. Co., Ltd (Shanghai, China), and their sequences are presented in Table S1. All DNA stock solutions were prepared with 1 × TE buffer (pH = 7.8, 10 mM Tris-HCl, 1 mM EDTA) from Sangon Biotechnology (Shanghai, China) and stored at 4°C for further use. Bovine serum albumin (BSA), Dulbecco's phosphate-buffered saline (D-PBS), Yeast RNA (tRNA) and LysoTracker Green were obtained from Beyotime Biotechnology (Jiangsu, China). Glucose and $MgCl_2·6H_2O$ were provided by Sinopharm Chemical Reagent Co., Ltd (Shanghai, China). Acryl/Bis 30% solution (29:1), tris hydrochloride (Tris-HCl), boric acid, ethylene diamine tetraacetic acid (EDTA), ammonium persulphate (APS), N,N,N',N'-tetramethylethylenediamine (TEMED), SYBR Green I and Hoechst 33342 were obtained from Dingguo Changsheng Biotech. Co., Ltd (Beijing, China). Adenosine triphosphate (ATP), T4 polynucleotide kinase (T4 PNK) and T4 DNA ligase were obtained from New England Biolabs Ltd (NEB, Beijing, China). Fetal bovine serum (FBS), Cell Counting Kit-8 (CCK-8), Dulbecco's modified Eagle's medium (DMEM, high glucose), RPMI-1640, penicillin/streptomycin solution, trypsin, doxorubicin HCl (Dox), Daunorubicin HCl (Dau) and Epirubicin HCl (Epi) were obtained from Dalian Meilun Biotech. Co., Ltd (Meilun, China). The DNA marker, 6 × loading buffer and 50 kD MerckMillipore Amicon® Ultra were obtained from Beijing Solarbio Science & Technology Co., Ltd (Beijing, China). Alexa Fluor 488-transferrin conjugate, Alexa Fluor 488-CTX-B conjugate and Alexa Fluor 488-dextran conjugates were provided by Fisher Scientific (Thermo Fisher Scientific Inc., U.S.A.). All other reagents were of analytical-regent grade. All the aqueous solutions used in this study were prepared with ultrapure water (resistance was 18.25 MΩ cm^{-1}) obtained from a Kerton lab MINI water purification system (UK).

SA.2 Cell culture

CCRF-CEM (human T-cell acute lymphocytic leukemia, abbreviated as CEM), Ramos (human B-cell Burkitt's lymphoma) and L02 (normal human hepatocytes) cells were purchased from Cell Resource Center of Shanghai Institute for Biological Sciences (Chinese Academy of Sciences, Shanghai, China). They were grown at 37°C in RPMI-1640 supplemented with 10% fetal bovine serum (FBS) and 1% penicillin-streptomycin in a humidified atmosphere of 5% CO_2. The cell density was determined using an ADAM-MC Auto Cell Counter (NanoEnTek Inc., Seoul, Korea) prior to each experiment. HeLa (human cervical cancer cells), A549 (human lung cancer cell) and MCF-7 (human breast cancer cell lines) were also obtained from the Cell Resource Center of Shanghai Institute for Biological Sciences (Chinese Academy of Sciences, Shanghai, China), which were cultured in the same conditions, but using the high glucose (4.5 g/L) version of Dulbecco's modified Eagle's medium (DMEM) supplemented with FBS and penicillin-streptomycin.

SA.3 Optimization of aptamer concentration for the assembly of sgc8-sNC materials

Since NC is constructed by 3D hierarchical assembly from small DNA building blocks and its structural unit is 3D rigid tetrahedron, the rigid structure, steric hindrance and negative charge-repulsion force could hamper the hybridization of external aptamers with the Apt-binding sites in the internal cavity. Thus, the amount of aptamer for the assembly of surface-scattered aptamer-conjugated NS (also called sgc8-sheathed nanocluster, sgc8-sNC) was optimized. First, various concentrations of Fe-sgc8 (0, 30, 60, 80, 100 and 120 nM) were separately incubated with the NC (200 nM) pre-constructed as shown in the section of "Stepwise assembly of DNA nanocluster" at 37°C for 2 h on TUS-200P constant temperature oscillation metal bath (Shanghai YIHENG Technical Co. Ltd., China), the free Fe-sgc8 (120 nM) as control. Then, a 10-μL aliquot of reaction sample was mixed with 2 μL of 6 × Loading buffer. The native polyacrylamide gel electrophoresis (nPAGE, 6%) analysis was carried out at 80 V for 60 min in a 0.5 × TBE buffer on an electrophoresis instrument. Finally, the gel imaging was performed in ChemDoc™ XRS instrument (Bio-RAD, USA).

SA.4 Specific recognition of target cancer cells

For cell imaging, different DNA assemblies or related probe samples were prepared in advance. Specifically, to prepare F-Lib sample, 0.5 μL of 10 μM F-Lib and 2.5 μL of 10 × T4 DNA ligase buffer were added into 22 μL of ultrapure water and mixed thoroughly. The final concentration of Lib is 200 nM. The same procedure was adopted to prepare F-sgc8 sample. The fluorescently-labeled sgc8-eNC and sgc8-sNC were separately assembled as described in the section of "Stepwise Assembly of DNA nanocluster", but Tb'-FAM was used instead of Tb'. For Lib-eNC, the assembly method is the same as sgc8-eNC, but e-Lib was used instead of e-sgc8. The final concentration of each assembled structure is 200 nM.

For suspension cells (CEM and Ramos), 5×10^4 cells were taken out and centrifuged (1200 RPM, 5min) to obtain cell pellets. After washing twice with washing buffer (D-PBS supplemented with glucose (final concentration, 4.5 g/L) and $MgCl_2$ (final concentration, 5 mM), the cell pellet was resuspended in 175 μL of binding buffer that was prepared by adding BSA (final concentration, 1 mg/mL) and Yeast RNA (final concentration, 0.1 mg/mL) into washing buffer. Then, 25 μL of DNA nanoclusters of interest was added and mixed thoroughly, followed by incubation at 37°C for 2 h in a humidified atmosphere of 5% CO_2. The resulting solution was centrifuged (1200 RPM, 5 min), and the pellet was washed twice with washing buffer (500 μL), followed by staining with Hoechst 33342 (200 μL) (diluting 10 mg/mL Hoechst stock solution with physiological saline solution to the final concentration of 10 μg/mL) for 15 min at room temperature. Subsequently, the cells were centrifuged (1200 RPM, 5 min) to remove residual Hoechst 33342 and resuspended in 20 μL of binding buffer. The cell sample was dropped into a confocal dish for confocal fluorescence microscopy analysis.

For adherent cells (HeLa and L02), 5×10^4 cells were cultured in a 24-well plate and cultured in complete medium (10% FBS and 1% penicillin-streptomycin) at 37°C in a humidified incubator containing 5% CO_2 overnight. After the cells were washed three times with PBS buffer (Meilun, Dalian), 25 μL of DNA NCs of interest and 175 μL of culture medium are added, followed by incubating in a humidified atmosphere of 5% CO_2 at 37 °C for 2 h. Next, the cell was washed twice with PBS and fix with 4% paraformaldehyde (Meilun, Dalian) for 15 min. After washing with PBS again, the cells were stained with Hoechst 33342 (10 μg/mL) for 5 min at room temperature. Finally, the cells were washed to remove the residual Hoechst 33342 and mounted on a microscope slide (Citotest, China) with Antifade Mounting Medium (Beyotime, Shanghai), followed by the fluorescence imaging. The confocal laser scanning microscope (CLSM) imaging was performed with a 20 × water objective on Leica SP8 laser scanning confocal microscope (CLSM, Leica, Germany). Hoechst 33342 was excited at a wavelength of 405 nm, while FAM was excited at 488 nm.

The cancer cell-targeting capability was also evaluated by flow cytometric analysis. The experimental procedures for preparing DNA nanoassemblies and subsequent incubation with the cells are the same CLSM imaging experiments, but 1×10^5 cells were used. Finally, CEM and Ramos cells were washed with washing buffer and re-suspended in 1 mL of PBS, while HeLa and L02 cells were digested with trypsin without EDTA (Meilun, Dalian) and resuspended in 1 mL of PBS. The fluorescence intensity of the four types of cell solutions (CEM, Ramos, HeLa and L02) was analyzed by counting at least 10000 cells on a BD FACSAria™ III flow cytometer (BD Biosciences, USA).

To pre-block the surface receptor binding sites, the cells were incubated with excess sgc8 aptamers (5 μL, 10 μM) in a humidified atmosphere of 5% CO_2 at 37°C for 1 h, followed by washing twice with PBS. The subsequent incubation with sgc8-eNC was performed according to the same procedure described as above.

SA.5 Investigation of endocytosis pathway

The fluorescence colocalization of Cy5-sgc8-eNC with the fluorescent biomarkers of different endocytosis pathways was evaluated by CLSM imaging of HeLa cells.[1, 2] First, 5×10^4 HeLa cells were cultured in a 24-well plastic-bottomed plate and grown to around 80% confluency. After

washing three times with PBS buffer, the cells were incubated with the mixture of 25 µL of Cy5-sgc8-eNC and 175 µL of culture medium in a humidified atmosphere of 5% CO_2 at 37°C for 2 h. Then, the culture medium was removed, and the cells were washed twice with PBS and incubated with the fluorescent biomarker of one endocytic pathway (200 µL) for 1 h at 37°C in a humidified atmosphere of 5% CO_2. The final concentration of fluorescence biomarkers is described as follows: 25 µg/mL for Alexa Fluor 488-transferrin conjugate, 1 µg/mL for Alexa Fluor 488-CTX-B conjugate and 2.5 mg/mL for Alexa Fluor 488-dextran conjugates. Afterward, the cells were washed twice with PBS and fixed with 4% paraformaldehyde for 15 min at room temperature, followed by staining with 10 µg/mL Hoechst 33342 for 5 min. Subsequently, 2 µL of Antifade Mounting Medium was added dropwise onto the center of cover slip and placed upside down on a microscope slide. The fluorescence images were taken on a Leica SP8 laser scanning confocal microscope. Hoechst 33342 was excited at the wavelength of 405 nm, while the excitations of Cy5 and Alexa Fluor 488 were set at 638 nm and at 488 nm, respectively.

Because the energy-dependent endocytosis is also an important endocytic pathway, the cellular uptake efficiency of sgc8-eNC at 4°C was evaluated. In brief, HeLa cells (1×10^5) were seeded in 12-well plates and incubated for 24 h. After washing the cells with PBS three times, 300 µL of FBS-free culture medium containing FAM-labelled sgc8-eNC was added and incubated at 4°C for 2 h. Afterward, the cells were collected by centrifugation after digestion with trypsin without EDTA, and the cellular uptake analysis was performed by flow cytometry.

To clarify the endocytosis pathway of sgc8-eNC, the influence of three different endocytosis inhibitors (amiloride, nystatin and sucrose) on the cellular uptake of sgc8-eNC was evaluated by flow cytometry. In brief, HeLa cells (1×10^5) were seeded in 12-well plates and incubated for 24 h. After the cells were washed three times with PBS, 300 µL of FBS-free culture medium solution containing one inhibitor (133 µg/mL amiloride, 15 µg/mL nystatin or 154 mg/mL sucrose) was added and incubated for 1 h. Then, the cells were washed three times with PBS and incubated with FAM-labelled sgc8-eNC at 37°C in a 5% CO_2 atmosphere for 2 h. Next, the cells were collected from the plate by centrifugation after digestion with trypsin in the absence of EDTA, and the uptake analysis was conducted by flow cytometry. The cells un-pretreated with any endocytosis inhibitor were used as control, and all experiments were carried out in triplicate.

SA.6 Colocalization study

The intracellular distribution of Cy5-labeled sgc8-eNC was evaluated by CLSM using HeLa cells as cell model. First, 5×10^4 cells were cultured in a 24-well plastic-bottomed plate and grown to around 80% confluency. After washing three times with PBS buffer, the cells were incubated with the mixture of 25 µL of Cy5-labeled sgc8-eNC and 175 µL of culture medium in a humidified atmosphere of 5% CO_2 at 37°C for 2 h. Subsequently, the cells were centrifuged, washed twice with PBS and incubated in 200 µL of culture medium for 0, 2 or 4 h. Then, the culture medium was removed, and the cells were washed twice with PBS and incubated with LysoTracker Green (50 nM) (a lysosome marker, Beyotime, China) for 30 min at 37°C in a humidified atmosphere of 5% CO_2. The resulting cells were washed twice with PBS buffer and fixed with 4% paraformaldehyde for 15 min at room temperature, followed by staining with 10 µg/mL Hoechst 33342 for 5 min. Next, 2 µL of Antifade Mounting Medium was added dropwise onto the center of cover slip and placed upside down on a microscope slide, followed by the fluorescence imaging. Hoechst 33342 was excited at the wavelength of 405 nm, while the excitations of Cy5 and LysoTracker Green were set at 638 nm and at 488 nm, respectively.

SA.7 Drug Loading

The fluorescence intensity of free Dox at different concentrations (0, 1, 2, 5, 10, 15, 20, 25 and 30 µM) in the absence of any DNA strand was measured to construct a standard linear calibration curve in advance, based on which the Dox-loading capability of sgc8-eNC was estimated.

To evaluate the Dox loading content, Dox (2 μM) was incubated with a given concentration (0, 70, 140, 200, 250, 300, 350 or 400 pM) of sgc8-eNC in a dark and dry bath incubator at 37°C for overnight. The resulting Dox-loaded sgc8-eNC solution was called Dox-sgc8-eNC (200 μL), and the corresponding fluorescence spectrum was measured on Hitachi F-7000 fluorescence spectrometer (Hitachi, Ltd., Japan). The instrument parameters were given as follows: EX WL, 495.0 nm; EM Start WL, 500.0 nm; EM END WL, 700.0 nm; Scan speed, 240 nm/min; EX Slit, 5.0 nm; EM Slit, 5.0 nm; PMT Voltage, 800 V; Response, 2.0 s. The amount of residual Dox (D_R) was estimated from the fluorescence intensity of the resulting solution. Along this line, Dox encapsulation efficiency (EE) was calculated according to the following equation: EE = $(D_T-D_R)/D_T \times 100\%$, where D_T indicates the total concentration of Dox (2 μM).

When evaluating the potential of sgc8-eNC as the drug nanovehicle to enhance cancer therapy outcome at the cellular level and on the organismic level in mice, sgc8-eNC (final concentration, 350 pM) was mixed with Dox (final concentration, 2 μM), and the resulting solution (200 μL) was incubated in a dark and dry bath incubator at 37°C for overnight, followed by centrifugation (10 min, 10000 RPM) at room temperature. The supernatant was removed, and the precipitates (Dox-sgc8-eNC) were washed three times and resuspended in 1 × T4 DNA ligase buffer (200 μL).

SA.8 Cytotoxicity analysis of DNA assembled nanostructures

To investigate the general cytotoxicity of sgc8-eNC vehicle itself, HeLa and L02 cells were plated in 96-well plastic-bottom plates and cultured in complete medium supplemented with 10% fetal bovine serum and 1% penicillin-streptomycin. The cells were cultured in a cell humidified incubator containing 5% CO_2 at 37°C and grown to 80% confluency. After the cells were washed twice with PBS and resuspended in 85 μL of cell medium, 25 μL of sgc8-eNC at a given concentration was added. The resulting mixture (100 μL) was incubated in a humidified atmosphere of 5% CO_2 at 37°C for 4 h. The final concentration of sgc8-eNC was 0, 280, 560, 840, 1120 or 1400 pM in each sample. Next, the cells were washed twice with PBS again and resuspended in 100 μL of fresh complete medium, followed by culturing for 20 h. Afterward, 10 μL of CCK-8 reagent was added into each cell well, and the cells continued to be incubated for 2 h. The absorbance intensity at 450 nm was recorded using NanoQuant Absorbance Microplate Reader (Tecan Infinite M200 PRO, Austria). The same experimental procedure was adopted for exploring the cytotoxicity to the suspension cells (CEM and Ramos).

The cytotoxicity of free Dox and Dox-sgc8-eNC was explored as described above, but sgc8-eNC was substituted with free Dox or Dox-sgc8-eNC. The final equivalent concentration of Dox was 0, 0.25, 0.5, 1.0, 15, 2.0, 2.5 or 3.0 μM. The cell viability was determined as follows:

$$(OD_{test}-OD_c) / (OD_{blank}- OD_c) \times 100\%$$

Where OD_{test} and OD_{blank} denote the optical density in the presence and absence of materials, respectively, while OD_c represents the optical density of culture medium.

SA.9 *In vivo* mouse xenograft model

BALB/c nude mice (male or female, 4-6 weeks old, about 20 g) were obtained from Fuzhou Wushi Animal Center and reared in a breeding cage with a sealed air filter at the temperature of 25-28°C and the relative humidity of 40-60%. All animal experiments were in accordance with the relevant regulations of the Institutional Animal Care and Use Committee (IACUC) of Fuzhou University. The HeLa cells (1×10^7) acquired by centrifugation and suspended in sterile PBS were subcutaneously injected into the right axillary location of nude mice. When tumor volume reached about 100 mm^3 and 400 mm^3, the mice were ready for examination of targeted therapy efficacy of Dox-loaded vehicles and analysis of *in vivo* biodistribution of vehicles themselves, respectively.

SA.10 Histopathological analysis by hematoxylin and eosin (H&E) staining

The tumors and organs (heart, liver, spleen, lung, and kidney) were collected and fixed in 4% paraformaldehyde for 48 h. Then, the tissues were embedded into paraffin, sectioned into slices, and

stained with hematoxylin and eosin (H&E). Histological analysis was performed by imaging on a Primo Star Upright Biological Microscope equipped with AxioCam ERc5s microscope camera (Carl Zeiss, Germany).

SA.11 Si-DLBM construction

DOPC liposome bilayer membrane-coated silicon dioxide microsphere (Si-DLBM) was prepared as described in the previous literature.[3] Briefly, 1,2-dioleoyl-sn-glycero-3-phosphocholine (DOPC, 5 mg, Aladdin, Shanghai, China) was dissolved in trichloromethane (5 mL, Sinopharm, Beijing, China) and incubated at 45°C in a vacuum drying oven overnight. Then, PBS (5 mL, Meilun, Dalian, China) was added and ultrasonically treated for 20 min, forming DOPC liposome bilayer membranes (DLBM). Meanwhile, silica microsphere solution (2.5 mg/mL, Saierqun, Tianjin, China) was ultrasonically treated for 5 min. Subsequently, an equal volume (2 mL) of two pre-treated solutions were mixed and incubated at room temperature for 1 h. The supernatant solution was removed by centrifugation at 10000 RPM for 10 min, and the microsphere precipitate was resuspended in 500 μL of ultrapure water. The washing step was repeated twice to remove the unwanted free compositions. Finally, the precipitate (Si-DLBM) was dispersed in 2 mL of 1 × T4 DNA Ligase buffer. The resulting Si-DLBM solution was sonicated at a frequency of 40 KHz for 5 min before each use.

SA.12 Dual-color fluorescence imaging of Si-DLBM

The Cy5-sgc8-eNC was assembled according to the procedure for sgc8-eNC, but Cy5e-sgc8 was instead employed. Similarly, the method for the assembly of Chol-FAM-NC is the same as NC, but the cholesterol-modified e-Tc (Chol-e-Tc) and Tb'-FAM were used instead of e-Tc and Tb', respectively. Then, equal volume of Cy5-sgc8-eNC and Chol-FAM-NC were mixed and incubated at room temperature for 2 h. After 5 μL of Si-DLBM was added, the resulting mixture was incubated at room temperature for 30 min, followed by centrifugation (10000RPM, 10 min). The supernatant was removed, and the precipitates were washed twice with 50 μL of PBS and resuspended in 20 μL of PBS. The CLSM imaging was performed with a 20 × water objective, while the excitation and emission were set as follows: FAM, λ_{ex} = 488 nm, λ_{em} = 518 nm; Cy5, λ_{ex} = 645 nm, λ_{em} = 670 nm.

Section B: Supplementary Discussion

SB.1 Characterization of DNA nanocluster.

Construction of Sgc8-eNC is accomplished by designing a specific binding site for sgc8 at vertex-iv of DNA tetrahedron (Tetra) unit followed by ligation-based sealing of nicks. In essence, sgc8-eNC is an aptamer-conjugated DNA nanocluster (NC) with a reserve pool of aptamers in the cavity. As the fundamental framework, the assembly yield and nanostructural morphology of bare basic DNA NC without aptamers, as well as its structural unit (bare Tetra), were firstly characterized by native polyacrylamide gel electrophoresis (nPAGE) analysis, atomic force microscopy (AFM) and dynamic light scattering (DLS). As shown in Figure S1, bare NC objects with a narrow size distribution can be efficiently assembled, and the assembly yield is almost 100% (no detectable byproducts). The dependence of structural features of NC on the concentration of building blocks was explored, and the optimal strand concentration is 200 nM as represented in Figure S2. As important functional domains for DNA self-assembly, the sticky-end association promotes hierarchical assembly. However, if the interaction between the sticky-ends is strong enough to overcome the strain energy penalty from the hybridization to neighboring structural components, the lateral growth will be hampered.[4, 5] For the successful assembly of DNA NC, the sticky end-based connection arms are designed to be responsible for the cross-linking between structural units and play a critical probe in the final formation of 3D backbone. Thus, the function of sticky ends was verified by mutating or deleting their nucleotides, and their sequences were optimized by changing the base nature to regulate their thermal stability. Firstly, we explored the influence of GC content in the sticky-ends on the assembly of NC because the GC content directly determines the sticky-end cohesion. The experimental results demonstrate that DNA Tetra with the sticky ends each containing three G/C bps is suited for use as the structural unit of DNA NC (Figures S3-S5). Increasing or decreasing the number of G/C bps of sticky ends inevitably causes the failure of NC assembly. In order to further study the dependence of NC assembly on the sticky-end cohesion, nine different DNA Tetra variants were designed by mutating or deleting some base pairs, including m/o-Tetra, o/m-Tetra, m/m-Tetra, m'/o-Tetra, o/m'-Tetra, m'/m'-Tetra, d/o-Tetra, o/d-Tetra and d/d-Tetra, where ‘m’ represents two mutant bases in a given sticky-end, ‘m'’ denotes seven mutant bases, "d" indicates the deleted sticky-end at a given connection arm and "o" represents the original sequence of sticky ends. The letter on the left of the slash indicates the change of Ta' sequence, while the one on the right demotes the change of Tb' sequence. More details are described in Figures S6, S8 and S10. The experimental data (Figures S6-S12) show that the mutation or deletion of bases in sticky end-ii and/or sticky end-iii can substantially affect the assembly of DNA NC, demonstrating the essential importance of appropriate interaction between sticky end-ii and sticky end-ia and between sticky end-iii and sticky end-ib. In addition, substituting 3D DNA Tetra with planar rigid triangle or common triangle cannot accomplish the NC assembly (Figures S13-S15). Moreover, shortening or extending the sticky ends also leads to the failure of the assembly of expected DNA nanoclusters (Figure S16). Overall, for the assembly of DNA NC, controlling number and nature of base pairs between complementary sticky ends is of great significance for the hierarchical assembly of NC from DNA Tetras, and the three dimensional shape of structural units is indispensable for the formation of well-defined nanostructures. The predictability and programmability of the interaction between DNA strands make it feasible to accomplish the assembly of hierarchical DNA NC in a precisely controllable manner by deliberately designing competent sticky ends and manipulating the spatial configuration of basic units.

Based on the above experimental results on the assembly of DNA NC, for the preparation of sgc8-eNC, equimolar quantities of seven building blocks (Ta, Tb, e-Tc, Ta', Tb', Tc' and LS) were added into 1 × T4 DNA ligase buffer and annealed (heated at 90 °C for 5 min and cooled gradually to room temperature) following the reported protocol.[6,7] Then, sgc8 and T4 DNA ligase were sequentially added to confer targeting capabilities through hybridization to Apt-binding site and rigidifying the 3D backbone by ligation-based sealing of nicks. The sgc8-eNC assembly process was

characterized by nPAGE analysis. As shown in Figure 1a, the assembled intermediates and products can be clearly observed at the corresponding positions determined by their own electrophoretic behaviors. Specifically, only one DNA band is seen in Lane 1, indicating the sufficient hybridization between Ta, Tb, and e-Tc. Afterward, an obvious mobility shift is detected upon addition of each component as shown in lanes 2-5, verifying the stepwise assembly of DNA Tetra. No coexisting several bands indicates that the predetermined arrangement of corresponding components, including LS responsible for the formation of scissors-shaped structure at vertex i, is well fulfilled. In Lane 6, upon e-sgc8 addition, DNA band shows the further increase in the gel retardation, revealing the formation of sgc8-incorporated DNA Tetra (sgc8-Tetra). Strikingly, a nonpenetrating DNA band appears in Lane 7 even without involving additional DNA strands, implying the successful assembly of sgc8-eNC with a hierarchical structure that has an extremely high molecular weight and spatial complexity due to the spatial organization of sgc8-Tetras onto ligation-rigidified 3D backbone. Moreover, a single, clean, and narrow band appears in each gel lane regardless of strand compositions, especially in Lane 7 (finally assembled products), indicating a narrow molecular weight distribution, almost 100% assembly efficiency and no byproducts generated during the assembly. Obviously, even if introducing the Apt-binding site into Tetra unit and installing sgc8, NC nanostructures are still monodispersed nanoobjects, validating the structural controllability of DNA NC at the molecular level.

To directly 'visualize' the structural features of DNA assembled products, sgc8-eNC and its structural unit were characterized by AFM and DLS. As shown in Figure S17b, DNA Tetra shows a geometrically well-defined configuration with average height (AH) of 2.6 nm that is roughly consistent with the literature reports.[8, 9] The AH smaller than theoretical height (4.7 nm on the basis of the assumption of 0.34 nm/base pair for the helical pitch) should be attributed to the structural collapse on the mica surface due to the dehydration required for AFM imaging in air and the tapping force.[10] Due to the AFM tip-broadening effect,[11] average width (AW) of 33.2 nm is slightly larger than theoretical value of 23.0 nm that is estimated from the 25-bp length in the edge of triangular bottom face and two connection arms each of which has 18 bases in length (including 11 bps in the ds-fragment and 7 bases in the ss-sticky end). The distance between two bases in a ss-DNA strand is considered to be 0.5 nm.[12] DLS analysis represented in Figure S17c displays that the population has a mean hydrodynamic diameter of 28 ± 1.0 nm, which is slightly smaller than the AW value calculated from AFM measurement. The theoretical diameter of the circumscribed sphere of bare Tetra unit is 25.0 nm that is in rough agreement with the DLS data. The hybridization with sgc8 causes the increase of NC size. As would be expected, Figure S18 shows that the size of sgc8-Tetras is slightly larger than bare DNA Tetra without aptamer (Figure S17). Specifically, although there is no change in the height (AH, 2.6 ± 0.1 nm) owing to easy collapse in the dehydrated state especially for the swinging ends, the AW value of sgc8-Tetras measured by AFM is 37.5 ± 0.6 nm and DLS hydrodynamic diameter is 35 ± 2 nm, demonstrating the hybridization of e-sgc8 to vertex-iv. No obvious aggregates reveal that there is no strong interaction between different DNA Tetras, which is reasonable because the ds-fragment of 7 bps even if formed between complementary sticky ends is not stable enough at room temperature. More excitingly, once the nicks were sealed by ligase, although there is no obvious change in structural morphology (spherical shape and quite uniform size distribution) (Figure 1b-iii and 1b-iv), the nanoparticle size significantly increases. The DLS diameter increases to 254 ± 30 nm (Figure 1b-i), while AFM analysis shows that the AW is 219.3 ± 5.6 nm and AH is 37.3 ± 0.2 nm (Figure 1b-v), proving that enzymatic ligation enables the formation of 3D cross-linked rigid DNA backbone onto which the Tetra units are arranged in a highly organized fashion. Namely, the expected sgc8-eNC nanostructure can be hierarchically constructed. TEM imaging was also performed to verify the formation of sgc8-eNC. As seen in Figure 1b-ii, sgc8-eNC exhibits a spherical shape with uniform size, and its average dimeter is about 140 nm that is smaller than the AFM value due to the need for the complete drying before TEM measurement.[13] Moreover, the number of sgc8-Tetra unit within each sgc8-eNC is 283 that was calculated from their own

volume ratio (see the discussion in Scheme S4), while the sgc8-eNC concentration is estimated to be 707 pM on the basis of the hypothesis that the assembly yield is considered to be 100% according to the nPAGE analysis in Figure 1a.

Besides several representative DNA nanoassemblies with different configurations were used as the controls in the current study, because sgc8-sNC is structurally closely related to sgc8-eNC and hold high drug-loading capability and high-density targeting ligands on its surface, it was used to sever as the direct control. Thus, the assembly of sgc8-sNC was also explored by nPAGE analysis, and the assembled objects were characterized by AFM imaging and DLS measurement. For this purpose, the DNA NC was first assembled and then subjected to Fe-sgc8 that is modified with FAM and can fluoresce upon light excitation. As shown in Figure S19a, Lane 1 is the DNA NC in the absence of Fe-sgc8 as the negative control, while Lane 7 is the Fe-sgc8 to show the band position of aptamer. No fluorescence signal in Lane 1 is detected because DNA NC cannot fluoresce in the absence of Fe-sgc8 (Lane 1). However, the brightness of 'DNA NC' band gradually increases with increasing the Fe-sgc8 concentration (lanes 2-5) and almost no Fe-sgc8 band is detected (very weak band in Lane 5), indicating the hybridization of Fe-sgc8 to DNA NC. Further increasing the Fe-sgc8 concentration to 120 nM, an obvious Fe-sgc8 band appears (Lane 6) that agrees very well with the band in Lane 7, suggesting the residual Fe-sgc8. Taking into account that the concentration of building blocks (e.g., e-Tc) is 200 nM in DNA NC solution, only half of Apt-binding sites are able to be employed for arranging aptamers. Namely, the Apt-binding sites in the internal cavity of NC are indeed inaccessible for e-sgc8. Thus, in other experiments, 100 nM e-sgc8 was used for construction of sgc8-sNC. Subsequently, the structural morphology of sgc8-sNC was characterized by AFM (Figure S19b). It can be seen that, compared with sgc8-eNC, sgc8-sNC is somewhat elliptical in shape and has a smaller size with an uneven distribution. The AH value is 33.1 ± 1.1 nm and the AW is 150.1 ± 7.0 nm. The DLS analysis shows that the hydrodynamic diameter is 160 ± 20 nm (Figure S19c), implying the sgc8-sNC has a more compact structure (the same concentration of building blocks is involved but the size is smaller). Possibly because of the steric hindrance determined by the structural features, e-sgc8 cannot sufficiently diffuse into the interior cavity to hybridize with Apt-binding sites. Even so, the sgc8-sNC still has many targeting ligands on its surface, can encapsulate many chemotherapeutic drugs Dox and is well suited to serve as the control for evaluation of powerful drug carriers.

From the small and compact structure of sgc8-sNC, one may know that sgc8-eNC has a tendency to contract and thereby the interior aptamers are squeezed. In this case, when the aptamers on the surface are displaced by hybridization to complementary strands or degraded by endonucleases and as such binding sites are vacated, the aptamers in the reserve pool of interior cavity will move outward to provide the compensation, remaining the cancer cell-targeting ability.

SB.2 Unique serum stability of aptamer-embedded DNA nanoclusters.

One of the most attractive features of sgc8–eNC is the enhanced resistance to enzymatic degradation in a complex biological milieu. Aptamer is well known to be easily degraded and often loses its inherent binding activity. So, it is very necessary to evaluate the serum stability of aptamer-conjugated nanostructures expected to have potential application in a biological setting, especially as a targeted drug delivery tool. To prolong the *in vivo* circulating half-lives required by the biological applications, aptamer probes often need to be subjected to chemical modification or combined with protective moieties to enhance the resistance to nuclease degradation.[14, 15] Therefore, due to the strong nuclease degradation in serum solution,[16] the serum stability of label-free sgc8–eNC was investigated by comparing with other DNA nanostructures before use in living organisms. As described in Figure S23a, a large portion of Fe-sgc8 is indeed degraded after 0.5-h incubation in 10% serum, and almost no residual strand is observed at 2-h point (panel v), indicating the high susceptibility of single-stranded oligonucleotide probes to nuclease-mediated degradation.[17] When Fe-sgc8 aptamer is installed at the vertex of DNA tetrahedron (F-sgc8-Tetra, where F denotes the FAM fluorophore), the

ability to resist nuclease degradation is obviously enhanced (panel iv) possibly due to the steric hindrance and structural rigidity against nuclease attack.[17, 18] Nevertheless, there is a very small amount of residual DNAs exposed to serum solution for 2 h. If Fe-sgc8 was hybridized to DNA nanowire made of tetrahedron units, the serum stability is further improved so that a certain amount of F-sgc8-NW is detected even if incubating in serum for 4 h (panel iii). Presumably this is because the synergistic anti-degradation effects of individual tetrahedral units efficiently prevent the nucleases from approaching the substrate surface to degrade the aptamer.[14] The moderate brightness of F-sgc8-sNC band appears at 8 h post-incubation in serum solution (panel ii), indicating the more efficient protection of targeting ligands against enzymatic degradation. More remarkably, the F-sgc8-eNC exhibits the substantially-enhanced serum stability so that no detectable decrease in the band brightness is observed throughout the period of examination (panel i). This should be attributed to the dense packaging of tetrahedron units that prevents the direct contact between nucleases and sgc8 molecules especially in the reserve pool of internal cavity, retarding the digestion process to a great extent. Figure S23b shows the quantitative evaluation of anti-degradation performance by quantifying the residual DNAs. The residual DNAs in different experimental groups were evaluated at 4-h incubation as follows: 97.3% for F-sgc8-eNC, 72.1% for F-sgc8-sNC, 32.1% for F-sgc8-NW, 2.1% for F-sgc8-Tetra and 0% for Fe-sgc8. As one can notice, even if F-sgc8-eNC was incubated in serum solution for 8 h, the residual amount of DNAs is more than 97%, indicating no obvious degradation. Namely, besides the desirable specific cancer cell recognition ability (Figure S20 and Figure S21), sgc8-eNC holds the remarkable serum stability, making it a potential drug delivery system *in vivo* for precision cancer therapy.

One remarkable advantage is that the sgc8-eNC exposed to rigorous serum treatment still can remain its target recognition ability almost unchanged. In order to offer the convincing evidence, the relationship between the cellular internalization efficiency of sgc8-eNC and serum pre-treatment time was evaluated by flow cytometry analysis. For this, the different DNA nanostructures (where e-sgc8 was still modified with FAM) were pre-treated with fresh mouse serum for a given time period and then incubated with HeLa cells. As shown in Figure S24a, for F-sgc8-eNC, the cell fluorescence almost does not change regardless of serum incubation time. For F-sgc8-sNC (Figure S24b), as the incubation time increases, the cellular internalization efficiency decreases. Nevertheless, there is still detectable fluorescence signal after 8-h serum incubation. For F-sgc8-NW (Figure S24c), an obvious decrease in cellular internalization efficiency is observed after 4-h serum incubation. When the serum incubation time reaches 8 h, the fluorescence signal basically equals to that of cell only. For F-sgc8-Tetra (Figure S24d) and Fe-sgc8 (Figure S24e), no obvious fluorescence signal is observed after 4-h or 8-h serum incubation, indicating the loss of internalization ability.

Moreover, the target recognition ability of fluorescently-modified sgc8-eNC pre-treated with serum solution was also explored by fluorescence imaging on the cellular level and on the organismic level. As shown in Figure S25, the dual-color fluorescence images shows that satisfactory Cy5 red fluorescence and FAM green fluorescence are observed for intact sgc8-eNC-FAM/Cy5 (panel 0-h) and a high degree of overlap (yellow in Merged) between the two fluorescence signals is achieved. Even if pre-treating sgc8-eNC-FAM/Cy5 with serum solution for a long time (panel 4-h and 8-h), no obvious decrease in the two fluorescence signal is detected, and the degree of colocalization also almost remains unchanged, indicating that the cellular internalization ability of sgc8-eNC-FAM/Cy5 is not obviously compromised and the structural integrity of sgc8-eNC-FAM/Cy5 is maintained even if being internalized into the cells. In contrast, for sgc8-sNC-FAM/Cy5 counterpart, if pre-treated under identical conditions, the obvious decrease in both red and green fluorescence signals is detected (panel 4-h). Moreover, upon 8-h serum pre-treatment, the fluorescence signal almost disappears, demonstrating the failure of cellular internalization due to the enzymatic degradation of aptamers on the surface.

For evaluation of target recognition ability of serum-pretreated sgc8-eNC *in vivo*, sgc8-eNC was modified with Cy5 and its accumulation in tumor sites was estimated by fluorescence imaging. As shown in Figure S26a, even if Cy5-sgc8-eNC was incubated in 10% serum solution for 4 h before administration into mice, the whole animal image shows a desirable fluorescence signal in tumor site. Moreover, the CLSM image of *ex vivo* tumor tissue slice displays that Cy5-sgc8-eNC does accumulate in tumor site, demonstrating that Cy5-sgc8-eNC survives from both serum pre-treatment and subsequent nuclease degradation in systemic circulation and finally enters into tumor tissues. In contrast, when administrating Cy5-sgc8-sNC into the mice under identical conditions, no obvious fluorescence signal is detected from tumor sites regardless of whether the whole-body fluorescence imaging or *ex vivo* tissue imaging (Figure S26b), implying almost no Cy5-sgc8-sNC within tumor tissues. If quantitatively evaluated by fluorescence signal, the amount of Cy5-sgc8-eNC accumulated in tumors is at least 60-fold higher than that of Cy5-sgc8-sNC. Presumably, although Cy5-sgc8-sNC is not substantially degraded at 4-h serum incubation (Figure S23) and still enters target cells *ex vivo* (Figures S24 and S25), after 4-h serum pre-incubation, it is particularly vulnerable to enzymatic degradation in an *in vivo* circulation system and cannot reach tumor sites. These experimental data prove that sgc8-eNC holds a significantly enhanced resistance to enzymatic degradation and suitable for real *in vivo* applications.

SB.3 Internalization pathway of DNA nanocluster.

As a molecular mechanism underlying the cellular uptake of nanoparticles, the endocytosis process is regulated by various mediators such as macropinocytosis, caveolae and clathrin.[19,20] Thus, the endocytic pathway of aptamer-embedded nanocluster was first explored by colocalization experiments with two-color confocal microscopy under the coexistence of Cy5-sgc8-eNC and different Alexa Fluor 488–labeled endocytic markers. Specifically, the transferrin was used as the marker of clathrin-mediated endocytosis, the cholera toxin subunit B (CTX-B) was employed as the marker of caveolae-mediated endocytosis, and the dextran served as the marker of micropinocytosis.[2] As shown in Figure S38a, Cy5 shows the considerable fluorescence colocalization (appearance of several clear yellow fluorescence spots) with Alexa Fluor 488–transferrin but displays less colocalization with Alexa Fluor 488–CTX-B and Alexa Fluor 488–dextran, indicating that sgc8-eNC can be taken up by target cells via clathrin-mediated endocytosis. This conclusion is also supported by quantitative colocalization analysis based on Pearson’s correlation coefficient (Rr), and Alexa Fluor 488–transferrin has much higher Rr value than the other two. To further explore the endocytic pathway, the relationship between the uptake efficiency of sgc8-eNC and incubation temperature or different inhibitors for specific internalization pathways was evaluated. As described in Figure S38b, the incubation at 4℃ extremely reduced the cellular uptake efficiency of sgc8-eNC, revealing that cellular internalization of sgc8-eNC follows an energy-dependent endocytic pathway since low temperature treatment can ‘block’ the energy-dependent endocytic pathway. [21-23] Meanwhile, the CLSM images show that, compared with the group of ‘Buffer’, that sucrose pre-treatment significantly inhibits the cell uptake of sgc8-eNC, while there is no obvious decrease upon the pre-treatment with amiloride or nystatin. It is well known that amiloride can inhibit the non-selective macropinocytosis,[24, 25] nystatin is significantly blocking the caveolae-dependent endocytosis,[26] and sucrose is a classical inhibitor for clathrin-mediated endocytosis.[18] Therefore, the clathrin-mediated endocytosis is confirmed to be responsible for the internalization of sgc8-eNC. The statistical results obtained by flow cytometry experiments (Figure S38c) are in good accordance with the CLSM imaging analysis. In addition, as shown in Figure S39, the inhibitors or incubation at 4℃ exhibit negligible cytotoxicity toward HeLa cells (cell viability of more than 90%), indicating that exposing the cells to these inhibitors or low temperature treatment won’t lead to the change in cellular behavior.

To study the intracellular dynamic distribution of sgc8-eNC, the fluorescence colocalization imaging was performed at different time points, where sgc8-eNC was attached with Cy5 to emit the fluorescence signal (red), while cell nuclei and lysosomes were stained with Hoechst and LysoTracker Green, respectively. As shown in Figure S38d, after incubation for 2 h, the obvious red

fluorescence of Cy5-sgc8-eNC within HeLa cells is detected and considerably overlaps with the green signal from LysoTracker Green, indicating that Cy5-sgc8-eNC can effectively take up by the cells and reside in the lysosome in the initial stage. With the extension of the incubation time (e.g., 6 h), the separation of red fluorescence spots from green signal occurs. The degree of fluorescence colocalization was also quantitatively assessed by Pearson's correlation coefficient (Rr) herein. As shown in panel 'Merged', the Rr value at 6 h is certainly lower than that at 2 h, implying the escape of some Cy5-sgc8-eNC formulations from lysosomes.

Section C: Supplementary Table

Table S1. Sequences of oligonucleotides designed in this work.

	Oligonucleotide	Sequence (from 5' to 3')
DNA tetrahedron	Strand a for tetrahedron assembly (Ta)	AGGAG ATACA CGATT ACAGC TTGCT ACACG ATTCA GACTT AGGAA TGTTC GATCA GGCAG GGTCC AAT
	Strand b for tetrahedron assembly (Tb)	GCAGT TGACA CGAAC ATTCC TAAGT CTGAA ATTTA TCCAT CGCCA TAGTA GACCT ATCAC CGTGG AAG
	Tetrahedron strand c (Tc)	CGTGT AGCAA GCTGT AATCG ACTCT ACCCT AAGTA AGCAA GACAC AACTA CTATG GCGAT GGATA AA
	Strand a' for tetrahedron assembly (Ta')	TAAGC CAGCC ATAAG TCAGT ATCTC CTATT GGACC CTGCC TGAAC ACTGA TCCG
	Strand b' for tetrahedron assembly (Tb')	GCGAG TATGG AGTCA ACTGC CTTCC ACGGT GATAG GATCG AATGC TGGAT ACGT
	FAM-modified Tb' (Tb'-FAM)	GCGAG TATGG AGTCA ACTGC CT**(FAM)**TCC ACGGT GATAG GATCG AATGC TGGAT ACGT
	Cy5-modified Tb' (Tb'-Cy5)	GCGAG TATGG AGTCA ACTGC CT**(Cy5)**TCC ACGGT GATAG GATCG AATGC TGGAT ACGT
	Strand c' for tetrahedron assembly (Tc')	CAGCA TTCGA AGTCC AGTTT CTGGA CATGT GTCTT GCTTA CTTAG GGTAG AGAAC CACGT TTCGT GGTAG ACTTA TGGC
	Linker strand (LS)	TGGCT TACGG ATCAG TGAAC CATAC TCGCA CGTAT C
	Extended Tc capable of capturing sgc8 (e-Tc)	ATGGA CCATC TCGAG ACATA TTTCG TGTAG CAAGC TGTAA TCGAC TCTAC CCTAA GTAAG CAAGA CACAA CTACT ATGGC GATGG ATAAA
Aptamer and its counterpart sequences	Extended sgc8 aptamer (e-sgc8)	TATGT CTCGA GATGG TCCAT TTTTT ATCTA ACTGC TGCGC CGCCG GGAAA ATACT GTACG GTTAG A
	Extended library DNA (e-Lib)	TATGT CTCGA GATGG TCCAT TTTTT GCGAG TATGT TGTCA ACTGC CTTCC ACTCT GATAT AAACC A
	FAM-modified extended sgc8 (Fe-sgc8)	**FAM**-T ATGTC TCGAG ATGGT CCA
	Cy5-modified extended sgc8 (Cy5e-sgc8)	**Cy5**-T ATGTC TCGAG ATGGT CCATT TTTTA TCTAA CTGCT GCGCC GCCGG GAAAA TACTG TACGG TTAGA
	FAM-modified sgc8 (F-sgc8)	**FAM**-T TTTTA TCTAA CTGCT GCGCC GCCGG GAAAA TACTG TACGG TTAGA
	FAM-modified library DNA (F-Lib)	**FAM**-T TTTTG CGAGT ATGTT GTCAA CTGCC TTCCA CTCTG ATATA AACCA
	Extended AS1411 aptamer (E-AS1411)	TATGT CTCGA GATGG TCCAT TTTTT **GGTGG TGGTG GTTGT GGTGG TGGTG G**
	Extended S6 aptamer (E-S6)	TATGT CTCGA GATGG TCCAT TTTTT **TGTGG CCAGT CACTC AATTG GGTGT AGGGG TGGGG ATTGT GGGTT G**

Testing aptamer movement	Cholesterol-modified e-Tc (Chol-e-Tc)	**Chol-** TTTAT GGACC ATCTC GAGAC ATATT TCGTG TAGCA AGCTG TAATC GACTC TACCC TAAGT AAGCA AGACA CAACT ACTAT GGCGA TGGAT AAA
	Strand complementary to e-sgc8 (Ce-sgc8)	TCTAA CCGTA CAGTA TTTTC CCGGC GGCGC AGCAG TTAGA TAAAA AATGG ACCAT CTCGA GACAT A
Tetrahedron of sticky end with deletions or mutations	Sticky end with two mutant bases in Ta' (m-Ta'-2)	TAAGA TAGCC ATAAG TCAGT ATCTC CTATT GGACC CTGCC TGAAC ACTGA TCCG
	Sticky end with two mutant bases in Tb' (m-Tb'-2)	GCGAG TATGG AGTCA ACTGC CTTCC ACGGT GATAG GATCG AATGC TGGAT ACAA
	Sticky end with seven mutant bases in Ta' (m-Ta'-7)	GCTAA TCGCC ATAAG TCAGT ATCTC CTATT GGACC CTGCC TGAAC ACTGA TCCG
	Sticky end with seven mutant bases in Tb' (m-Tb'-7)	GCGAG TATGG AGTCA ACTGC CTTCC ACGGT GATAG GATCG AATGC TGACG TGAA
	Sticky end-deleted Ta' (d-Ta')	GCCAT AAGTC AGTAT CTCCT ATTGG ACCCT GCCTG AACAC TGATC CG
	Sticky end-deleted Tb' (d-Tb')	GCGAG TATGG AGTCA ACTGC CTTCC ACGGT GATAG GATCG AATGC TG
Planar DNA triangle	Planar-triangle-strand a (p-Ta)	AGGAG ATACA ACAGC TTGCT ACACA CCAGA CTGGA ATGTC GATCA GGCAG GGTCC AAT
	Planar-triangle-strand b (p-Tb)	GCAGT TGACC CGACA TTCCA GTCTG ACCAT CGCCA TAGTA GACCT ATCAC CGTGG AAG
	Planar-triangle-strand c (p-Tc)	TGTGT AGCAA GCTGT ACTCT ACCCT AAGTA AGCAA GACAC AACTA CTATG GCGAT GGT
	Strand d for assembling hollow triangle (h-Td)	GCAGT TGACA TCAGG CAGGG TCCAA TAGGA GATAC ACTCT ACCCT AAGTA AGCAA GACAC AACCT ATCAC CGTGG AAG
Tetrahedron of sticky end with different G/C ratio	Sticky end with one GC in linker strand (LS-1)	TTACT TACGG ATCAG TGAAC CATAC TCGCA CTTAT A
	Sticky end with one GC in Ta' (Ta'-1)	TAAGT AAGCC ATAAG TCAGT ATCTC CTATT GGACC CTGCC TGAAC ACTGA TCCG
	Sticky end with one GC in Tb' (Tb'-1)	GCGAG TATGG AGTCA ACTGC CTTCC ACGGT GATAG GATCG AATGC TGTAT AAGT
	Sticky end with six GC in linker strand (LS-6)	TGGCC GCCGG ATCAG TGAAC CATAC TCGCG CGTCG G
	Sticky end with six GC in Ta' (Ta'-6)	GCGGC CAGCC ATAAG TCAGT ATCTC CTATT GGACC CTGCC TGAAC ACTGA TCCG
	Sticky end with six GC in Tb' (Tb'-6)	GCGAG TATGG AGTCA ACTGC CTTCC ACGGT GATAG GATCG AATGC TGCCG ACGC

Tetrahedron of sticky ends with different base numbers	Sticky end with four bases in linker strand (4-LS)	TGGCC GGATC AGTGA ACCAT ACTCG CACGC
	Sticky end with four bases in Ta' (4-Ta')	GCCAG CCATA AGTCA GTATC TCCTA TTGGA CCCTG CCTGA ACACT GATCC G
	Sticky end with four bases in Tb' (4-Tb')	GCGAG TATGG AGTCA ACTGC CTTCC ACGGT GATAG GATCG AATGC TGGCG T
	Sticky end with ten bases in linker strand (10-LS)	TGGCT TAAAT CGGAT CAGTG AACCA TACTC GCAAT ACGTA TC
	Sticky end with ten bases in Ta' (10-Ta')	ATTTA AGCCA GCCAT AAGTC AGTAT CTCCT ATTGG ACCCT GCCTG AACAC TGATC CG
	Sticky end with ten bases in Tb' (10-Tb')	GCGAG TATGG AGTCA ACTGC CTTCC ACGGT GATAG GATCG AATGC TGGAT ACGTA TT
	Sticky end with fifteen bases in linker strand (15-LS)	TGGCT TAAAT CATGC CGGAT CAGTG AACCA TACTC GCGTG ACAAT ACGTA TC
	Sticky end with fifteen bases in Ta' (15-Ta')	GCATG ATTTA AGCCA GCCAT AAGTC AGTAT CTCCT ATTGG ACCCT GCCTG AACAC TGATC CG
	Sticky end with fifteen bases in Tb' (15-Tb')	GCGAG TATGG AGTCA ACTGC CTTCC ACGGT GATAG GATCG AATGC TGGAT ACGTA TTCAC TG

Notice: The color-filled fragment represents the aptamer sequence (yellow for sgc8 aptamer, blue for AS1411 aptamer, and green for S6 aptamer). The underlined domain indicates the sticky end. Extended library DNA (e-Lib) indicates that aptamer domain is substituted with a random base sequence. The fragment with green background indicates the base change compared with the original strand. The fragment with gray background indicates the number of G/C base pairs formed in the assembled nanoclusters. Ta, Tb, Ta', Tb', Tc' and LS were phosphorylated at their 5' ends before the assembly of DNA nanoclusters.

Section D: Supplementary Results and Discussion

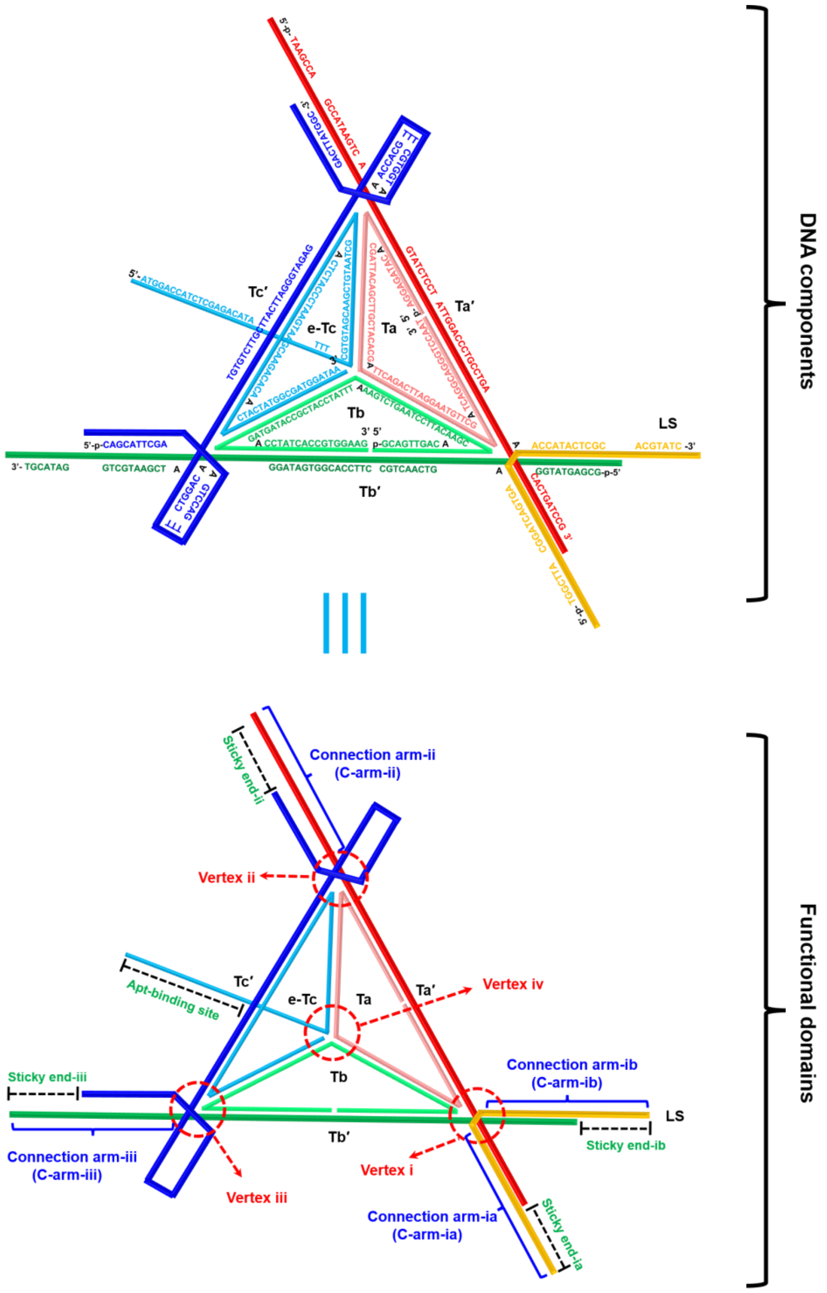

Scheme S1. Schematic drawing of DNA tetrahedron unit, accompanied by the base sequence of DNA components and functional domains. The functional domains include five ss-fragments: four sticky ends that are arranged to the triangular bottom face, while the binding site of aptamer at vertex iv. Except e-Tc, the DNA components were phosphorylated as indicated by the letter 'P'. Of note, the functional domains of DNA tetrahedron variants (e.g., Figures S4, S6, S8, S10, S13 and S14), including the names of vertexes and sticky ends, are named by referring to this tetrahedron.

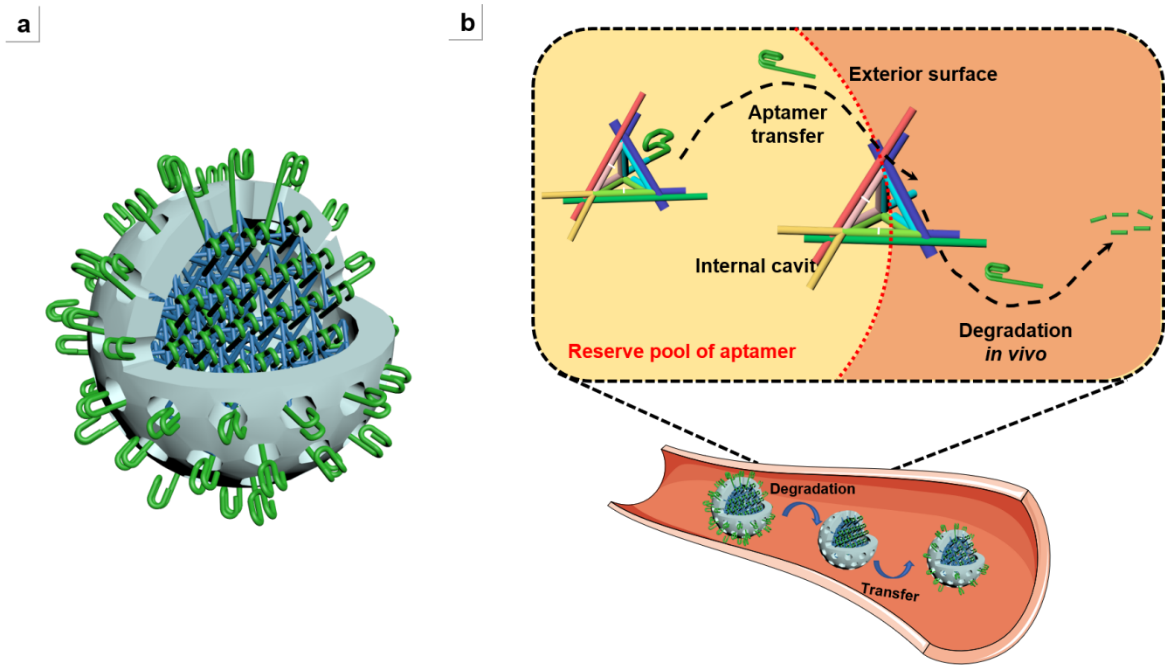

Scheme S2. (a) Three-dimensional cartoon view to show the structure of sgc8-eNC. There are abundant aptamers in the internal cavity (reserve pool) of sgc8-eNC and on its surface since aptamer is hybridized to DNA tetrahedron unit before the ligation-based sealing of nicks. (b) Molecular mechanism for the maintenance of desirable specific cancer-cell recognition even if encountering the enzymatic digestion. The aptamers in the internal cavity serve as the reserve pool and can move outward to function if the surface-scattered aptamers are degraded after exposing sgc8-eNC to a detrimental physiological environment.

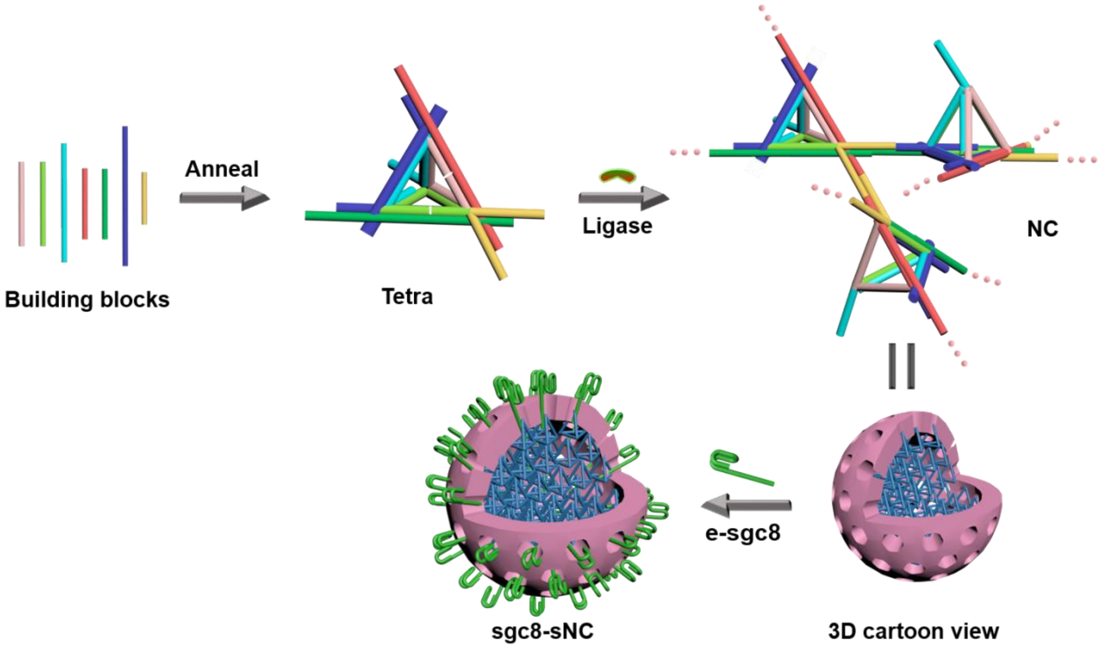

Scheme S3. Schematic illustration of the assembly of aptamer surface-scattered nanocluster (sgc8-sNC). The aptamers only reside on the surface of nanoparticle because the assembly of DNA NC is accomplished before addition of aptamers as such aptamers cannot move inward due to electrostatic repulsion/steric hindrance and thereby are unable to hybridize to the binding sites in the internal cavity.

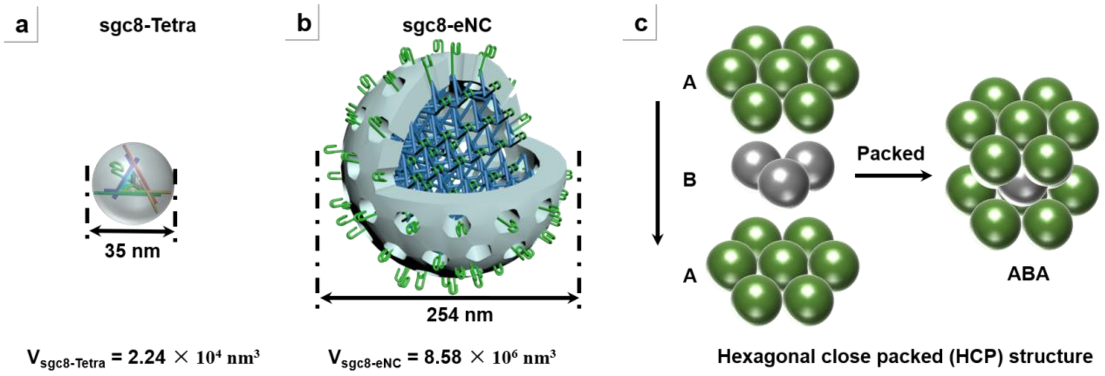

Scheme S4. Estimating the number of sgc8-Tetra unit in one sgc8-eNC particle. The diameter and corresponding volume of sgc8-Tetra (a) and sgc8-eNC (b) were measured by dynamic light scattering (DLS). (c) The putative hexagonal close packed (HCP) ordered structure for theoretical calculation of sgc8-Tetra number.

Discussion:

Although AFM image shows the flat shape of sgc8-eNC due to the collapse of 3D DNA structures associated with the dehydration process,[27] for the analysis of DLS data, DNA assemblies could be assumed to be spherical particles in a solution.[28] According to DLS data, the diameter of sgc8-Tetra ($D_{sgc8\text{-}Tetra}$) is 35 nm (Figure S18) and the diameter of sgc8-eNC ($D_{sgc8\text{-}eNC}$) is 254 nm (Figure 1b). On the basis of the well-known formula ($V=4\pi R^3/3$) for calculating the volume of a sphere, the corresponding volumes of sgc8-Tetra and sgc8-eNC are 2.24×10^4 nm^3 and 8.58×10^6 nm^3, respectively (Schemes S4a and S4b). If the basic structural units are assumed to be arranged in a HCP arrangement style to make full use of the space (every four adjacent spheres are tangent in pairs to form a tetrahedron, Scheme S4c), the filling factor is 74%.[29-31] Along this line, the number of sgc8-Tetra units in one sgc8-eNC is estimated to be 283 by utilizing the following equation:

$$V_{sgc8\text{-}eNC} \times 74\ \% \ / \ V_{sgc8\text{-}Tetra} = 8.58 \times 10^6 \times 74\% \ / \ 2.24 \times 10^4$$

On the basis of the above calculation results, because one sgc8-eNC has 283 sgc8-Tetra structural units, one structural unit has one set of building blocks at an equal molar ratio and the concentration of each building blocks in the reaction solution is 200 nM, the sgc8-eNC concentration is estimated to be 707 pM.

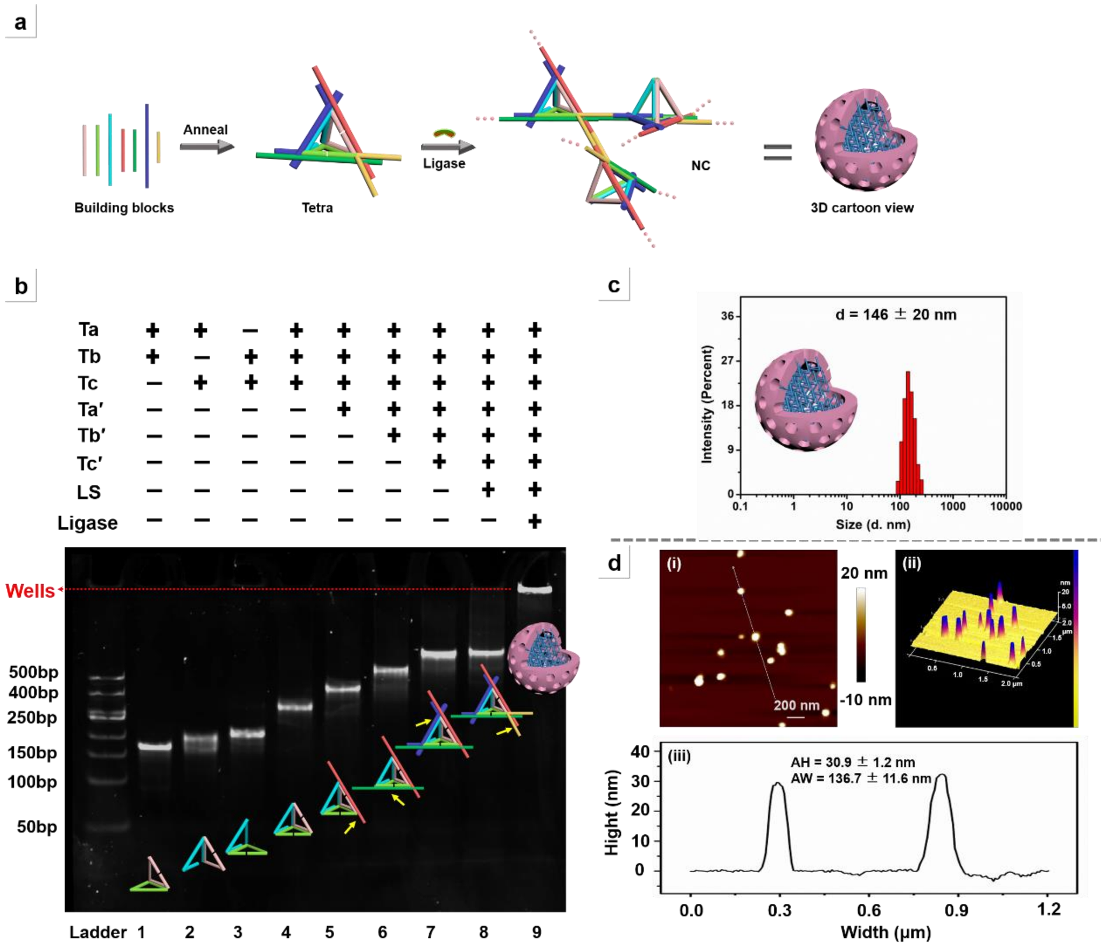

Figure S1. Characterization of basic NC consisting of DNA tetrahedra. (a) Schematic assembly of DNA nanocluster (NC) from DNA building blocks. (b) nPAGE (6%) analysis to characterize the stepwise assembly of DNA NC. The red arrow is the position of gel/well interface. (c) DLS measurement of hydrodynamic diameter of DNA NC; (D) AFM image (i) of DNA NC, corresponding 3D tapping-mode topographic image (ii) and cross-section profile (iii) along the white line shown in part i. Scale bar = 200 nm. AH and AW denote the average height and average width (n = 20), respectively. In the assembly solution, the concentration of each DNA building blocks is 200 nM, while the concentration of T4 DNA ligase is 8 U/μL. The working solution is 1 × T4 DNA ligase buffer.

Experimental procedure:

Bare basic NC object without targeting ligands was assembled as described in the section of "Stepwise assembly of DNA nanocluster". For DLS characterization, the sample was 10 times diluted with 1 × T4 DNA ligase buffer. The DLS measurement was carried out on a Malvern Instruments Zetasizer HS III (Malvern, UK) at room temperature according to the method described in the section of "Dynamic light scattering measurement". Similarly, the sample was diluted by a factor of 10 with 1 × T4 DNA ligase buffer, and AFM measurement was performed according to the procedure described in the section of "AFM characterization of DNA nanocluster (NC)". All the measurements were conducted in triplicate, and the values are expressed as means ± standard deviation (SD).

Discussion:

The DNA NC assembly is outlined in Figure S1a. As shown in Figure S1b, only one obvious band is observed in Lanes 1, 2 and 3, indicating the complete hybridization between every two DNA components. Subsequently, the shift in electrophoretic mobility is observed upon introduction of one DNA building block (Lanes 4 to 8), indicating the stepwise assembly, including the hybridization to LS responsible for the formation of scissors-shaped structure at vertex i. Strikingly, a nonpenetrating DNA band appears in Lane 9,

implying the advanced assembly of sgc8-eNC after encountering ligase even without adding additional DNA strands. The ligation-based sealing of nicks makes DNA Tetra aggregate by the cross-linking between different connection arms, leading to the dramatic increase in molecular weight and spatial complexity. Moreover, no bands of building blocks and unwanted byproducts appear in each lane, demonstrating that the assembly yield at each step is almost 100%. Moreover, clean single band appears in each gel lane regardless of strand compositions, especially in Lane 9 (basic NC nanostructure), implying a narrow molecular weight distribution, almost 100% assembly efficiency and no byproducts generated. This also indicates a desired controllable assembly process, enabling the structural control over the expected functional modification (e.g., introduction of targeting ligand) for specific recognition and targeted cellular uptake.

DNA NC was further studied by microscopic characterization techniques. As could be expected, upon addition of ligase, the size of DNA assembled products dramatically increases. Specifically, the DLS diameter increases to 146 ± 20 nm (Figure S1c), while AH and AW recorded from AFM measurement increase to 30.9 ± 1.2 nm and 136.7 ± 11.6 nm, respectively (Figure S1d-iii). Even if the size would generally become small in the dehydrated state, the height and width are much larger than DNA Tetra (Figure S17b). Similarly, the DLS diameter of DNA NC is also much larger than DNA Tetra (Figure S17c), manifesting the substantial aggregation of DNA Tetra units. This offers the convincing evidence that ligation-induced sealing of nicks triggers the advanced assembly of DNA Tetra units as such these units are tightly tied together by the cross-linking of connection arms.

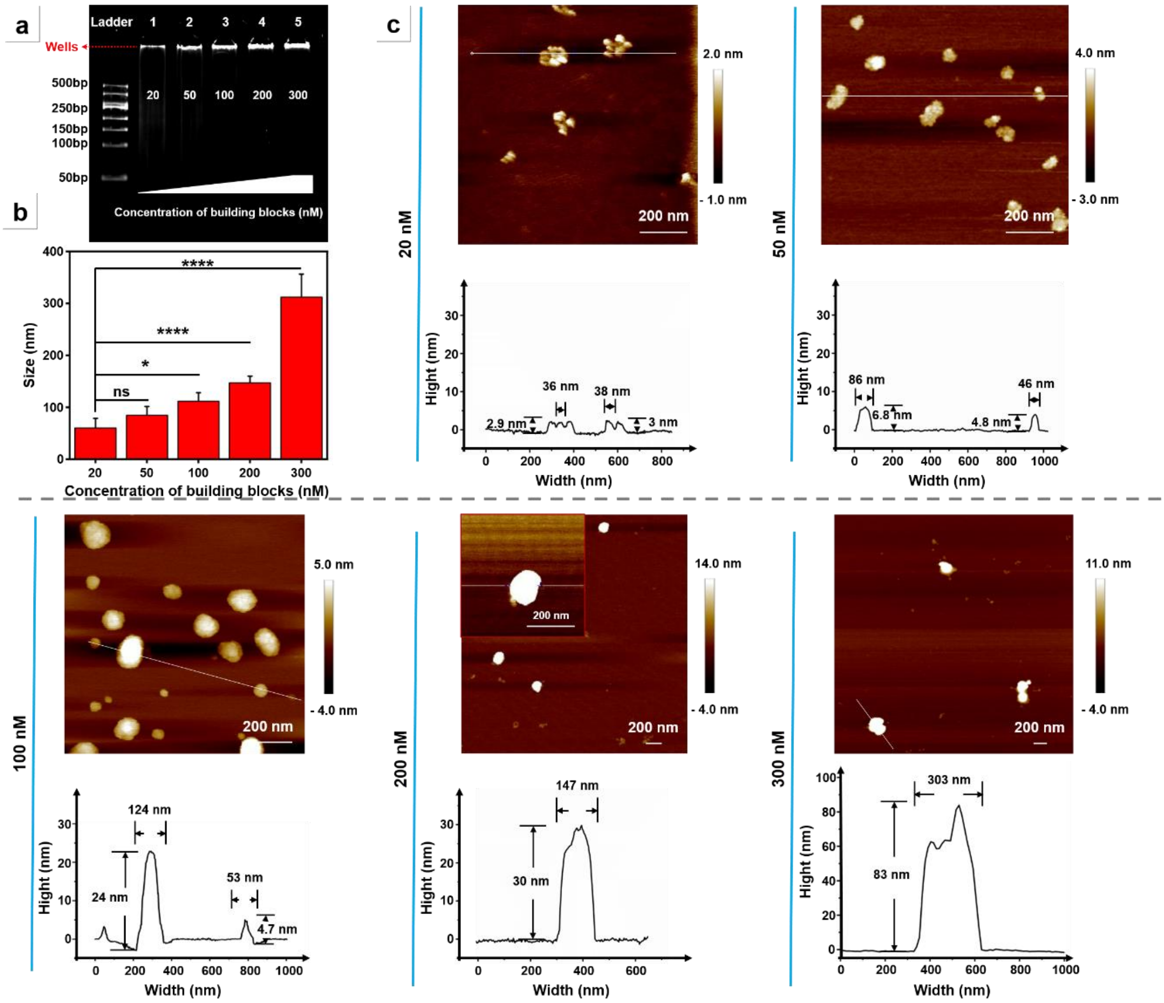

Figure S2. Influence of the concentration of building blocks on the assembly of DNA NC. (a) nPAGE (6%) analysis to characterize DNA nanoclusters assembled from different concentrations of building blocks: 20 nM, 50 nM, 100 nM and 200 nM. The red arrow indicates the position of gel/well interface. (b) Hydrodynamic diameter of corresponding DNA NCs measured by DLS. All the DLS measurements were conducted in triplicate, and the values are expressed as means ± standard deviation (SD). The statistical analysis of multiple comparisons were evaluated by one-way ANOVA. *P < 0.05, **P < 0.005, ***P < 0.0005, ****P < 0.0001, and ns represents no significant difference. (c) AFM images taken to furher characterize DNA NCs. Lower half of each panel: the corresponding cross-section profiles along the white lines in the upper half.

Discussion:

The construction of DNA nanostructures is an inter-unit process that is dependent on the concentration of building blocks.[32, 33] In this case, it is more difficult to assemble the large objects, such as 2D and 3D architectures, that often need micromolar and even submillimolar concentration of components.[34] Thus, the dependence of DNA NC on the building block concentration was explored by different methods. As shown in Figure S2a, nPAGE image indicates that DNA assembled products appear and are retarded at the initial position of the gel regardless of building block concentration, indicating the assembly of DNA nanoobjects even at low concentrations. The corresponding DLS data are shown in Figure S2b. It can be seen that nanoobject size shows an increasing trend with the concentration increase. Moreover, the morphology and size distribution do not necessarily concur with the expectations. In order to intuitively observe the assembled nanoclusters, the dependence of DNA NC on the building block concentration was further explored by AFM characterization. As shown in Figure S2c, the nanoclusters formed at a concentration of 20 nM show the petal-shaped structure, implying the raw accumulation of several Tetras. The cross-sectional analysis of representative nanoclusters reveals that the height of each petal is lower than 3 nm and the width is about 36 nm (approximately equals to the width of a Tetra, Figure S17b). With the increase in

building block concentration from 50 nM, 100 nM, 200 nM and to 300 nM, the nanoclusters with gradually increasing size were obtained. Specifically, the AH size of particles is not more than 100 nm and uneven at 50 nM, the sizes of assemblies are markedly different at 100 nM and geometrical configuration is irregular at 300 nM, suggesting a fundamentally uncontrollable assembly process. Very interestingly, the clusters assembled at 200 nM exhibit spherical morphology and uniform size distribution, and they have the height of about 30 nm and the width of about 147 nm, demonstrating an efficient assembly technique that is amenable to the construction of 3D DNA nanostructures and superior to the conventional methodologies [35, 36] that need much higher concentration (1 to 100 μM) of building blocks to construct a simple DNA tetrahedron and uncomplicated 1D DNA architecture. In the subsequent experiments, DNA NC assembled at 200 nM was used.

a

Sample	GC contents	Putative sticky end	Complementary sticky end
(i)	1-GC	5'-TAAGTAAAAAAA-3'	5'-TTACTTAAAAAA-3'
(ii)	2-GC	5'-TAGCTAAAAAAA-3'	5'-TTAGCTAAAAAA-3'
(iii)	3-GC	5'-TAAGCCAAAAAA-3'	5'-TGGCTTAAAAAA-3'
(iv)	4-GC	5'-TAGGCCAAAAAA-3'	5'-TGGCCTAAAAAA-3'
(v)	5-GC	5'-TCGGCCAAAAAA-3'	5'-TGGCCGAAAAAA-3'
(vi)	6-GC	5'-GCGGCCAAAAAA-3'	5'-TGGCCGCAAAAA-3'
(vii)	7-GC	5'-GCGGCCGAAAAA-3'	5'-CGGCCGCAAAAA-3'

b

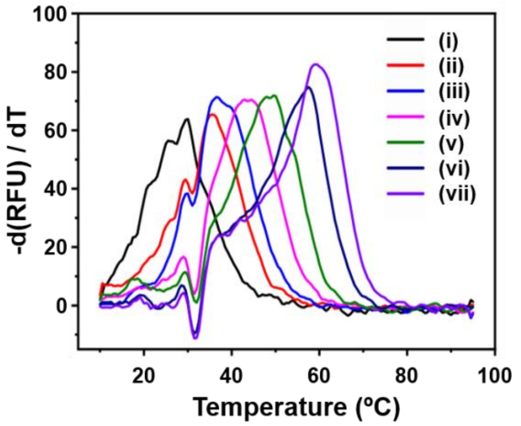

c

Sample	GC contents	T_m
(i)	1-GC	29.50 °C
(ii)	2-GC	34.50 °C
(iii)	3-GC	36.50 °C
(iv)	4-GC	44.50 °C
(v)	5-GC	49.50 °C
(vi)	6-GC	57.50 °C
(vii)	7-GC	59.00 °C

Figure S3. The dependence of melting behavior of different double-stranded (ds) fragments on the content of G/C base pairs (GC content). (a) The base sequences of putative randomly-designed short fragments and their complementary strands, accompanied by corresponding GC contents. (b) Melting curves of putative fragment/complementary strand hybrids (called ds-putative hybrids, dp-hybrids). (c) Melting temperature (T_m) of dp-hybrids recorded from panel b.

Experimental procedure:

Melting temperature (T_m) analysis was performed to evaluate the thermodynamic stability of the hybrids formed from hybridization between complementary putative short fragments. Firstly, 2.5 μL of 10 × T4 DNA ligase buffer and equal amounts of putative fragment and its complementary strand (2 μL, 10 μM) were added into 18.5 μL of H_2O and mixed well. After heating at 90°C for 5 min, the mixture was gradually cooled down to room temperature, forming the ds-hybrid. Next, 18 μL of resulting solution and 2 μL of SYBR Green I (10 ×) were added to a 0.2-mL PCR tube (Labselect, USA), followed by mixing and incubating for 10 min to allow SYBR Green I to fully interact with dsDNA. The Tm analysis was performed on a CFX96^{TM} real-time PCR detection system (Bio-RAD, Singapore). The temperature was increased from 10°C to 95°C, and the heating rate was 0.5°C/30 s. Three parallel measurements were performed for each sample, and the measured data were analyzed by using the software supplied with the instrument. Because the fluorescence intensity is proportional to the amount of SYBR Green I intercalated into DNA double helix structure, T_m can be estimated from the fluorescence signal of SYBR Green I.

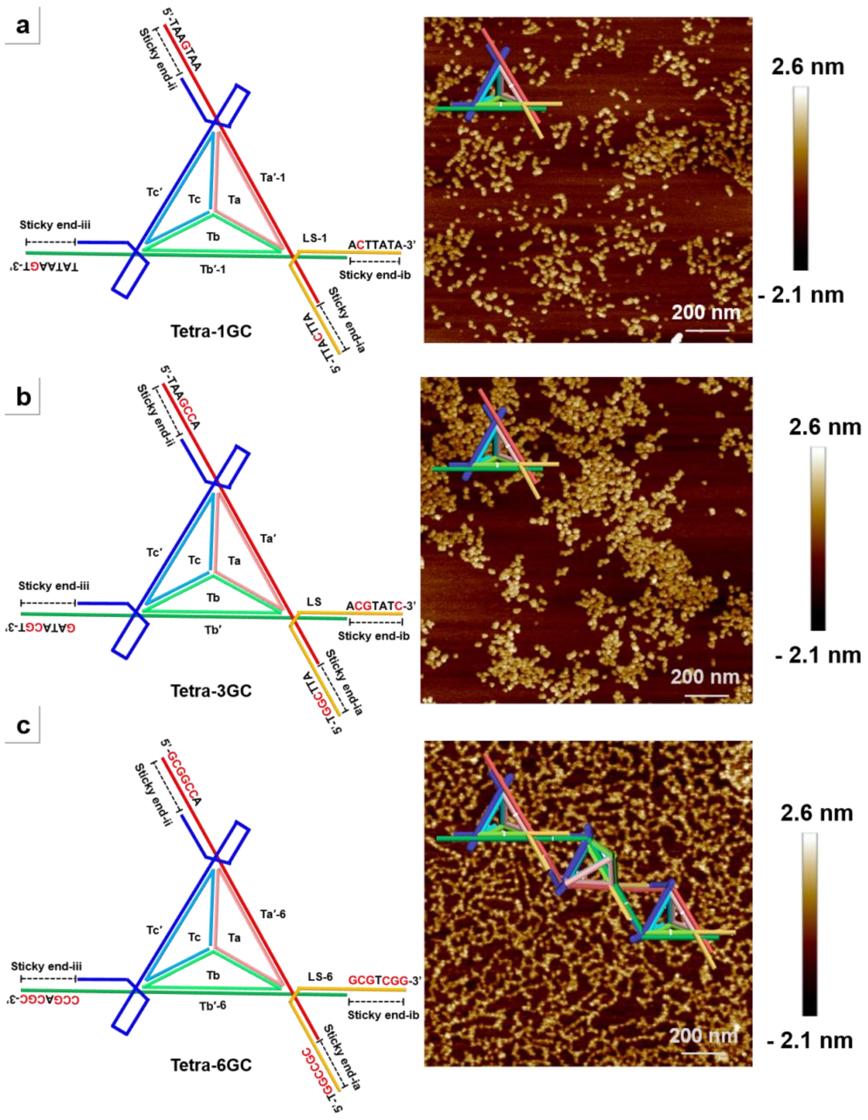

Figure S4. The relationship between DNA tetrahedron morphology and GC content in the sticky ends. (a) AFM characterization of DNA tetrahedron with 1-G/C base-incorporated sticky ends (called Tetra-1GC). (b) AFM characterization of DNA tetrahedron with 3-G/C base-incorporated sticky ends (called Tetra-3GC). (c) AFM characterization of DNA tetrahedron with 6-G/C base-incorporated sticky ends (called Tetra-6GC). The left parts show the corresponding schematic diagram. The three DNA tetrahedrons were assembled according to the same experimental procedure described in the section of "Stepwise assembly of DNA nanocluster".

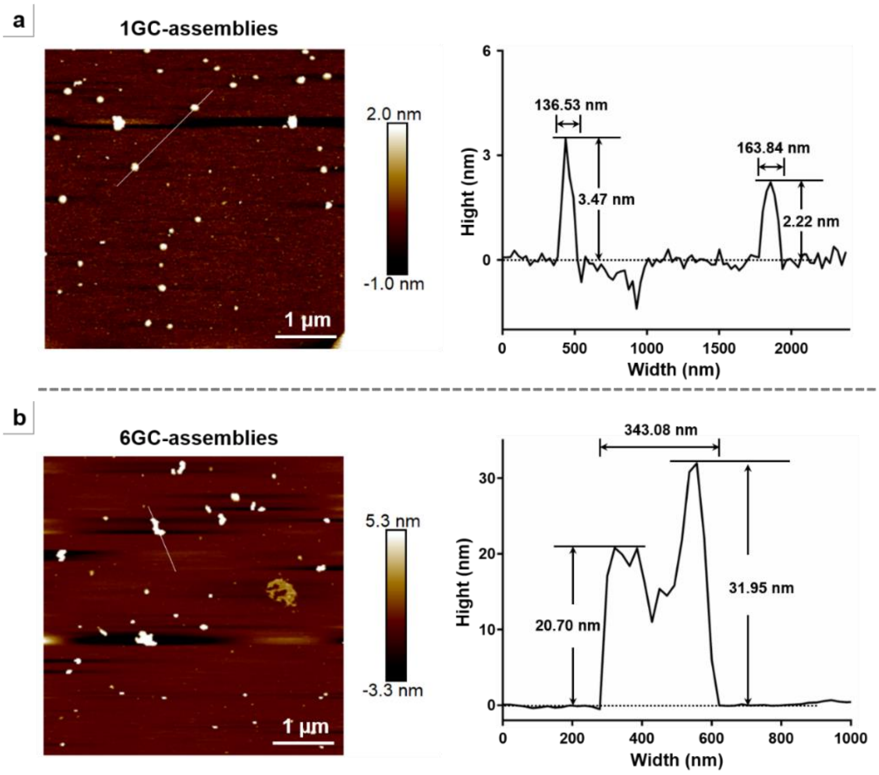

Figure S5. Structural morphology of ligated nanostructures assembled from DNA tetrahedra with the sticky ends of different GC contents. (a) AFM image of ligated products using Tetra-1GC as structural unit (called 1GC-assemblies), accompanied by the cross-section analysis. (b) AFM image of ligated products using Tetra-6GC as structural unit (called 6GC-assemblies), accompanied by the cross-section analysis. Due to the large size polydispersity of objects, only representative assembled products rather than statistical analysis are involved when performing the cross-section analysis.

Experimental procedure:

Tetra-1GC was assembled as described in the section of "Stepwise assembly of DNA nanocluster", but Ta'-1, Tb'-1 and LS-1 were used instead of Ta', Tb' and LS, respectively. Subsequently, T4 DNA ligase (0.5 µL, 400 U/µL) was added and incubated at 16°C overnight to seal the nicks in the edges and the hybridized sticky ends, followed by incubating 65°C for 10 min to terminate the ligation. Then, the resulting solution was diluted 10 times with 1 × T4 DNA ligase buffer for AFM measurement. The AFM characterization of 6GC-assemblies was performed according to the same procedure, but Ta'-6, Tb'-6 and LS-6 were instead used.

Discussion for Figures S3-S5:

The dependence of the interaction between sticky-ends on the G/C content was firstly explored by Melting temperature (T_m) analysis. The several fragments with different G or C numbers and corresponding complementary ones are shown in Figure S3a, while their melting curves and T_m are represented in Figure S3b and Figure S3c, respectively. One can see that T_m of hybridized fragments increases with the increment in the number of G/C base pairs, demonstrating that the interaction between connection arms can be indeed regulated by changing the G/C content of sticky ends. Along this line, three DNA tetrahedrons (Tetra-1GC, Tetra-3GC and Tetra-6GC) with different sticky ends were assembled and characterized by AFM measurement. The experimental results are represented in Figure S4, accompanied by the structural diagrams. Varying the association strength between the sticky-ends causes substantial difference in the morphology between DNA assemblies. It can be seen that most of Tetra-6GC are threaded into flexible wires where bumps and indentations are alternatively aligned (Figure S4C). The possible reason is that, due to the high G/C content, the sticky end-ia and sticky end-ib in one DNA Tetra stably hybridize with sticky

end-ii and sticky end-iii of another Tetra, respectively, formatting the head-to-tail multiple tandem repeats. In comparison, when the sticky ends cannot stably hybridize with their complementary fragment by reducing the low G/C content, Tetra-3GC units are not regularly organized (Figure S4b) and especially considerable amount of Tetra-1GC particles exist in a very dispersed manner (Figure S4a). Figure S5a shows more information. One can notice that the final assemblies from Tetra-1GC upon the ligation shows obvious structural polydispersity, and the height is roughly equivalent to that of Tetra units (Figure S17), indicating the failure of subsequent hierarchical assembly. If the G/C content in the sticky ends is too high (e.g., using Tetra-6GC as structural unit), some very large nanostructures are formed upon the facilitation effect of ligation. Moreover, the assembly process is uncontrollable, and the majority of assembled products are small (Figure S5b). Only Tetra-3GC can be amenable to the construction of DNA NC (seen in Figure S1 and Figure 1). Therefore, the Tetra-3GC was adopted in the current study. Unless otherwise indicated, DNA Tetra or structural unit mentioned has three G/C bases in each sticky end.

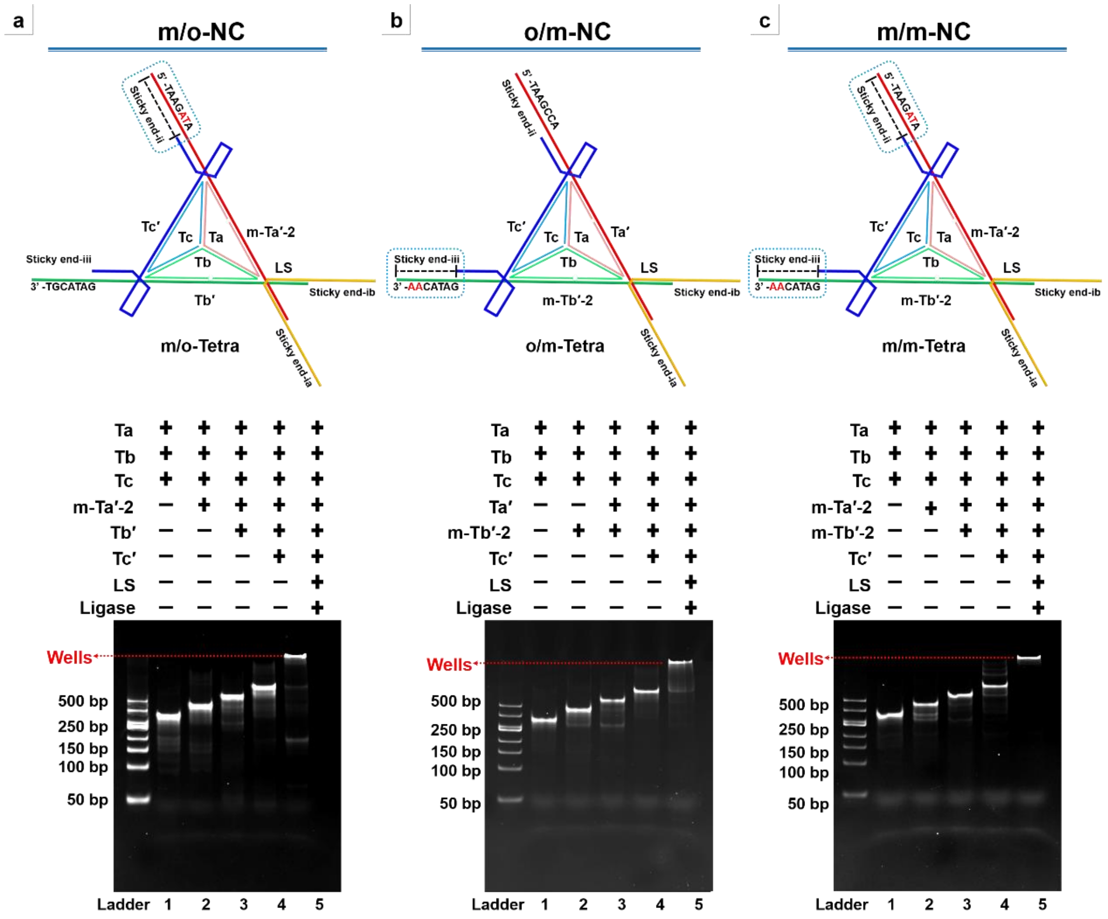

Figure S6. Assembly of DNA NC from tetrahedron unit with two mutant bases (red) in the sticky end-ii and/or sticky end-iii. (a) The m/o-Tetra (two mutant bases in the sticky end-ii) was used as the structural unit for the assembly of DNA nanocluster. The resulting assembled product is called m/o-NC; (b) The o/m-Tetra (two mutant bases in the sticky end-iii) as the structural unit for the assembly of DNA nanocluster. The corresponding product is called o/m-NC; (c) The m/m-Tetra (two mutant bases in each of the sticky ends ii and iii) as the structural unit for the assembly of DNA nanocluster. The corresponding product is called m/m-NC. The letters 'm' and 'o' indicate one building block with mutant (m) and original (o) sequence, respectively, in the sticky end-ii and/or sticky end-iii. The upper part shows the structural representation of DNA Tetra unit, while the lower part is the nPAGE image. The red arrow is the position of gel/well interface. To discuss the mutant base-contained products, the expected DNA nanocluster assembled from the building blocks without any mutant base is called o/o-NC in this section, and its corresponding structural unit is called o/o-Tetra (the same as Scheme S1 but without Apt-binding site).

Experimental procedure:

All the three mutant NC nanostructures were assembled according to the procedure described in the section of "Stepwise assembly of DNA nanocluster", but m-Ta'-2 was used instead of Ta' for m/o-NC, m-Tb'-2 was used instead of Tb' for o/m-NC and m-Ta'-2 and m-Tb'-2 were instead used for m/m-NC. The as-assembled NC sample (8 μL) was mixed with 2 μL of 10 × SYBR Green I and 2 μL of 6 × Loading buffer and incubated at room temperature for 5 min. The nPAGE was run at 80 V for 60 min in a 0.5 × TBE buffer on a gel electrophoresis instrument (Bio-RAD, USA). The gel was photographed on a Bio-RAD ChemDoc^{TM} XRS (Bio-RAD, USA), and the image analysis was performed with the Image Lab software (Bio-RAD, USA).

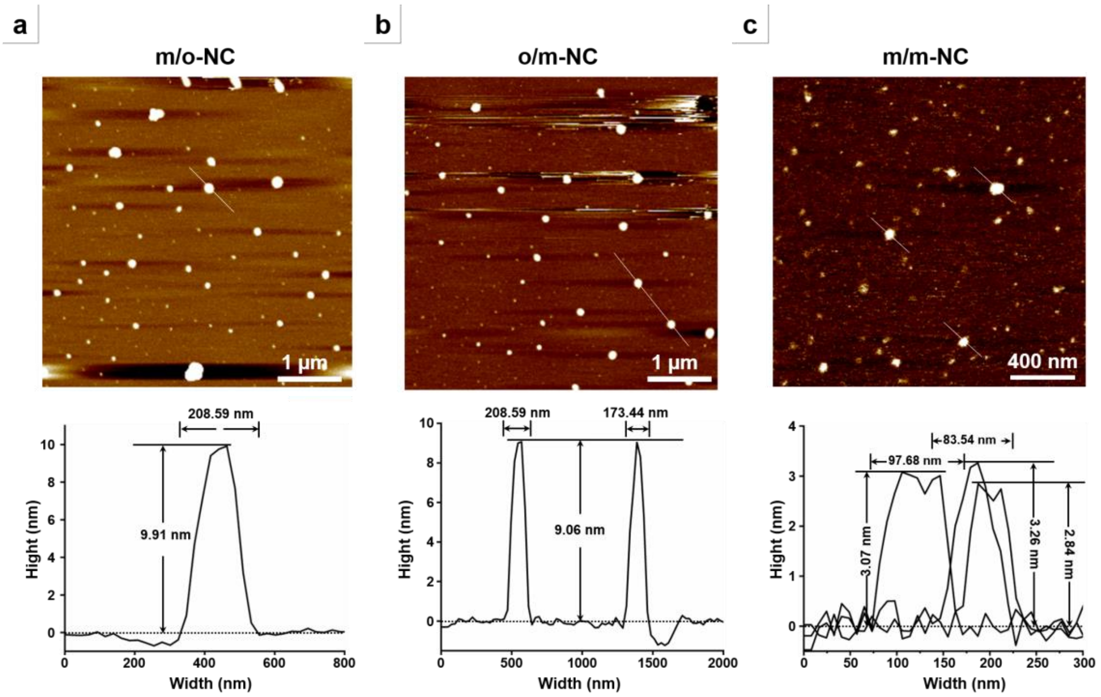

Figure S7. AFM characterization of three DNA assemblies with two-base-mutated sticky ends. AFM images of (a) m/o-NC, (b) o/m-NC, (c) m/m-NC. The lower part is the corresponding cross-section profiles along the white lines. Due to the large size polydispersity of objects, only representative assembled products rather than statistical analysis are involved when performing the cross-section analysis.

Discussion for Figures S6 and S7:

Three different Tetra units (m/o-Tetra, o/m-Tetra, m/m-Tetra) were designed for the assembly of different mutant NC nanostructures. For the m/o-NC, as shown in Figure S6a, the migration rate of bands in Lanes 1-4 gradually becomes slower, indicating the hybridization reaction upon the addition of each building block. When the LS and ligase were added, DNA bands do not display obvious migration in the gel (Lane 5). This should be attributed to the formation of m/o-NC that almost cannot penetrate into the gel, in which the hybridization of LS to DNA Tetra promotes the interaction between DNA Tetras and the sealing of nicks by T4 DNA Ligase rigidifies the DNA backbone and Tetra units. The similar phenomena were observed for the stepwise assembly of o/m-NC (Figure S6b) and m/m-NC (Figure S6c).

To further characterize the mutant assembled NC objects, AFM imaging was performed. As shown in Figure S7a, although the shape of some m/o-NCs is similar to o/o-NC (AH=30.9 nm, AW=136.7 nm, Figure S1), its height is much lower (9.91 nm), while its width is substantially larger (208.59 nm), indicating that the assembled structures are distinctly different from each other. The possible reason is that, even if only one sticky end contains two mutant bases, the mutant sticky end in one Tetra cannot efficiently hybridize with its complementary fragment in another Tetra and structural collapse easily occurs, thus disabling the formation of rigid 3D structure of DNA NC. Similar AFM data are observed for o/m-NC (Figure S7b). For m/m-NC (Figure S7c), the AFM image shows the smaller and uneven size. Specifically, its height is roughly equal to normal DNA tetrahedron (Figure S17) and its width is about two times larger than normal DNA tetrahedron, indicating the dimer of m/m-Tetra rather than a 3D NC architecture consisting of many structural units.

The experimental results demonstrate that m/o-NC, o/m-NC, and m/m-NC are the unanticipated structures. Namely, the base mutation in the sticky ends can cause the failure of DNA NC assembly.

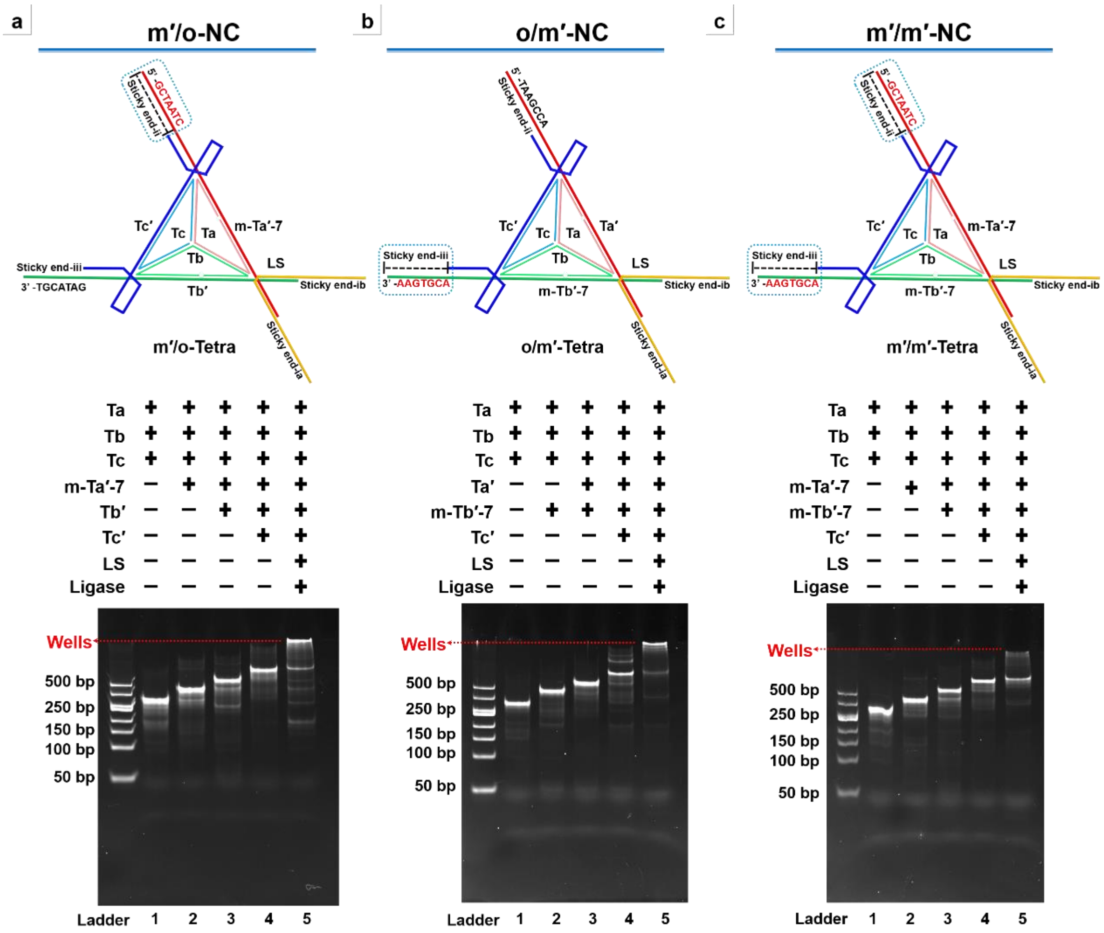

Figure S8. Assembly of mutant DNA NCs from tetrahedron unit with seven mutant bases (in red) in the sticky end-ii and/or sticky end-iii. (a) The m'/o-Tetra (seven mutant bases in the sticky end-ii) was used as the structural unit for the assembly, forming the m'/o-NC; (b) The o/m'-Tetra (seven mutant bases in the sticky end-iii) as the structural unit for the assembly, generating the o/m'-NC; (c) The m'/m'-Tetra (seven mutant bases in each of the sticky ends ii and iii) as the structural unit for the assembly, generating the m'/m'-NC. The letter ‘m'’ (different from the ‘m’ mentioned in Figure S6) indicates that one building block has more (seven) mutant bases in a given sticky end. The upper part shows the structural representation of DNA Tetra unit, while the lower part is the nPAGE image. The red arrow is the position of gel/well interface. The experiments were performed as described in Figure S6, but m-Ta'-7 and m-Tb'-7 were substituted for m-Ta'-2 and m-Tb'-2, respectively.

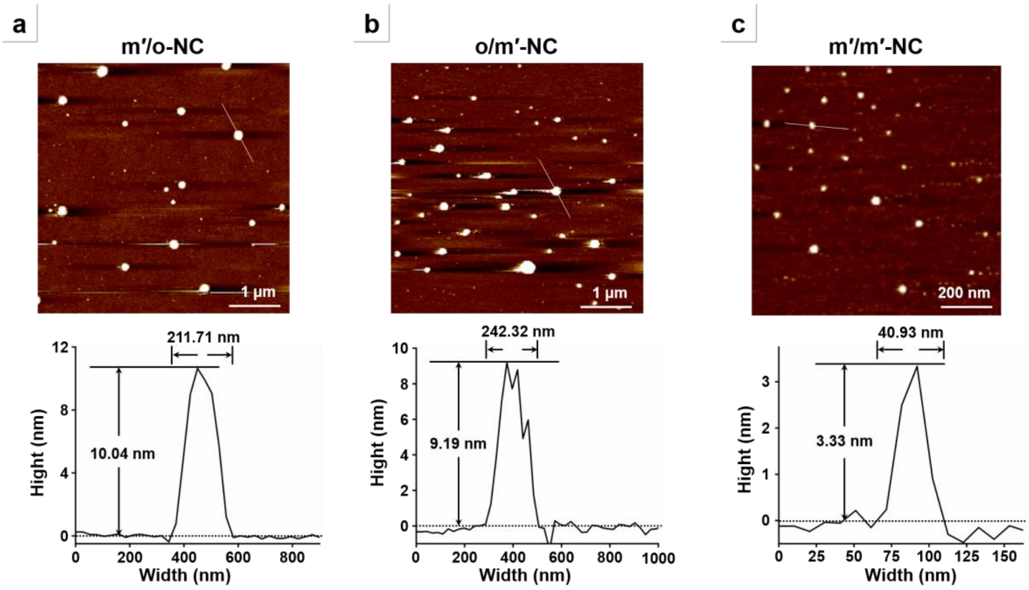

Figure S9. AFM characterization of three DNA assemblies with seven-base-mutated sticky ends. AFM images of (a) m'/o-NC, (b) o/m'-NC, (c) m'/m'-NC. The lower part is the corresponding cross-section profiles along the white lines. Due to the large size polydispersity of assembled objects, only representative products rather than statistical analysis are involved when performing the cross-section analysis.

Discussion for Figures S8 and S9:

As shown in Figure S8a, addition of each building block retards the electrophoretic mobility of DNA bands (lanes 1-4), indicating the stepwise hybridization of DNA components. When the LS and ligase were added, DNA bands do not display obvious migration in the gel (Lane 5), which should be due to the formation of m'/o-NC with large molecular weight (MW) that almost cannot penetrate into the gel. However, besides the main band, the appearance of a lower band with the relative low brightness in lanes 2, 3, 4 and 5 implies the incomplete assembly of building blocks into NC structure. Figure S8b displays the similar experimental phenomena. Moreover, the immobile bands with the large MW disappear in Lane 5 of Figure S8c, indicating the failure of the interaction between m'/m'-Tetra units. Namely, seven mutant bases existing in the sticky end-ii and/or sticky end-iii are more unfavorable for the hierarchical assembly of 3D nanoclusters.

Moreover, AFM imaging was performed to offer more information on the assembly. As shown in Figure S9a, although the spherical shape of m'/o-NC is observed, it has a much lower height (10.04 nm) and substantially larger width (211.71 nm) than o/o-NC (AH=30.9 nm, AW=136.7 nm, Figure S1), indicating the structural collapse due to the mutant sticky end-ii. The similar AFM images are observed from o/m'-NC (Figure S9b). When the m'/m'-Tetra was used as the structural unit with both mutant sticky end-ii and mutant sticky end-iii, the height and width of assembled product (m/m-NC) become much smaller and are roughly equal to that of DNA tetrahedron (Figure S17). Namely, there is no obvious interaction between the structural units (m'/m'-Tetra), indicating the impossibility of 3D assembly.

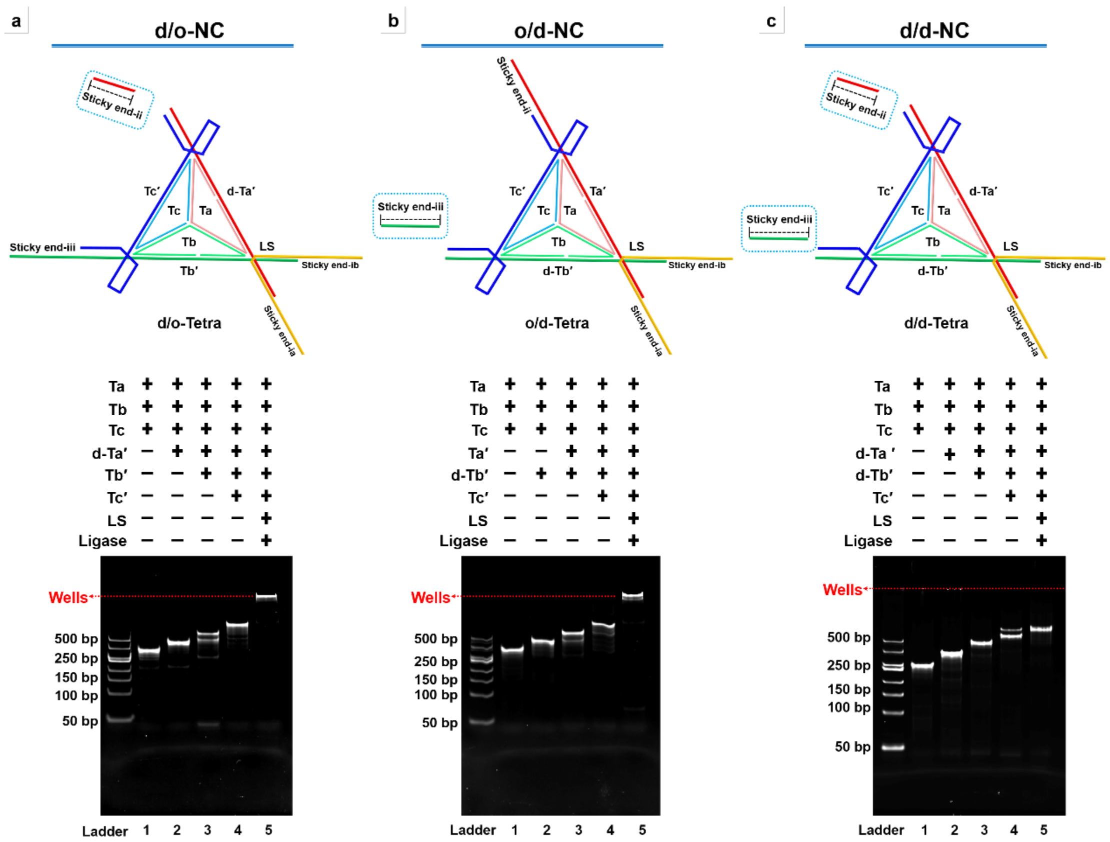

Figure S10. Assembly of DNA NCs from tetrahedron unit without the sticky end-ii and/or sticky end-iii. The deleted sticky ends are indicated by free fragments. (a) The d/o-Tetra without the sticky end-ii at vertex-ii was used as the structural unit for the assembly, forming the d/o-NC; (b) The o/d-Tetra without the sticky end-iii at vertex-iii as the structural unit for the assembly, generating the o/d-NC; (c) The d/d-Tetra, where both the sticky end-ii and the sticky end-iii were respectively removed from vertex-ii and vertex-iii, as the structural unit for the assembly, obtaining the d/d-NC. The letter 'd' indicates the deletion of sticky ends. The upper part shows the structural representation of DNA Tetra unit, while the lower part is the nPAGE image. The stray fragments are the domains deteted from the sticky ends, and the red arrow is the position of gel/well interface. The experiments were performed using the same procedure as Figure S6, but d-Ta' and d-Tb' were used instead of m-Ta'-2 and m-Tb'-2, respectively.

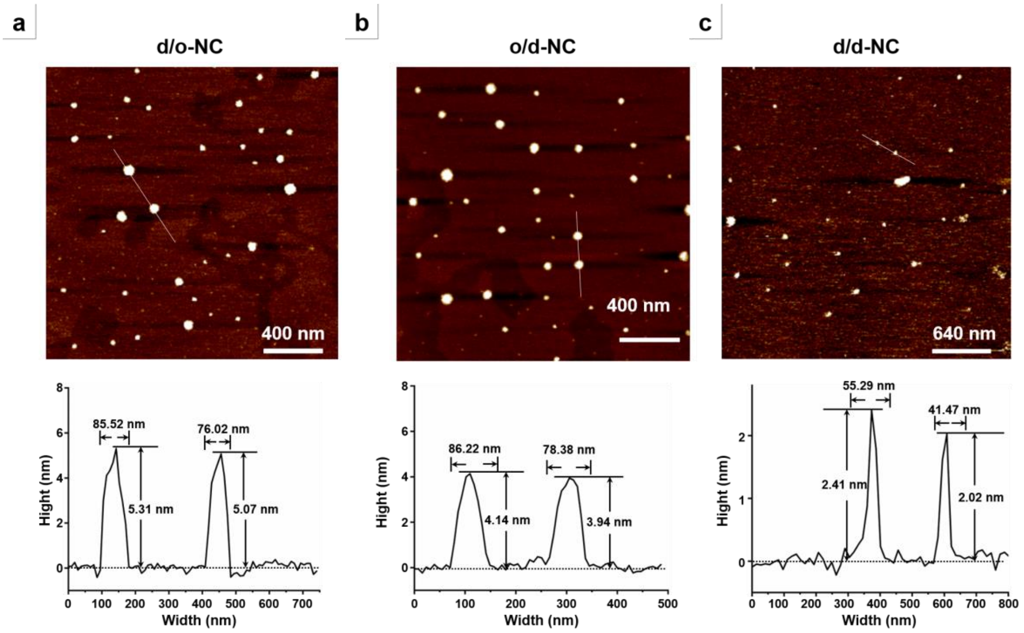

Figure S11. AFM characterization of DNA NCs assembled from tetrahedron unit without the sticky end-ii and/or sticky end-iii. AFM images of (a) d/o-NC, (b) o/d-NC and (c) d/d-NC. The lower part is the corresponding cross-section profiles along the white lines. Due to the large size polydispersity of objects, only representative assembled products rather than statistical analysis are involved when performing the cross-section analysis.

Discussion for Figures S10 and S11:

As shown in Figure S10a, the migration rate of bands in lanes 1-4 gradually decrease upon the addition of more building blocks, indicating the stepwise hybridization. The appearance of byproduct band in Lane 3 implies the deteriorated assembly capability. No obvious band migration in Lane 5 should be owing to the formation of d/o-NC with large molecular weight (MW). Similar experimental phenomena are observed for o/d-NC as shown in Figure S10b. When both sticky end-ii and sticky end-iii are deleted, the assembled products with large MW disappear (seen in Lane 5 in Figure S10c), indicating no interaction between DNA d/d-Tetra units.

AFM images provide the more information as shown in Figure S11. There is no obvious difference in the shape and size between d/o-NC (Figure S11a) and o/d-NC (Figure S11b), which is reasonable because only one sticky end is deleted from their structural unit. Compared with o/o-NC (AH=30.9 nm, AW=136.7 nm, Figure S1), their height and width are much smaller. Moreover, though the height is slightly higher than normal DNA tetrahedron (AH=2.6 nm, AW=33.2 nm, Figure S17) possibly duo to the rigidity associated with the hybridization of the other two sticky ends between different Tetra units, the width is only about two times larger than Tetra monomer. Naturally, d/o-NC and o/d-NC are considered to be the dimer composed of their own structural units. After deleting the two sticky ends, the resulting(Figure 11c) d/d-NC displays the size similar to normal DNA tetrahedron, indicating that d/d-NC essentially is the d/d-Tetra monomer.

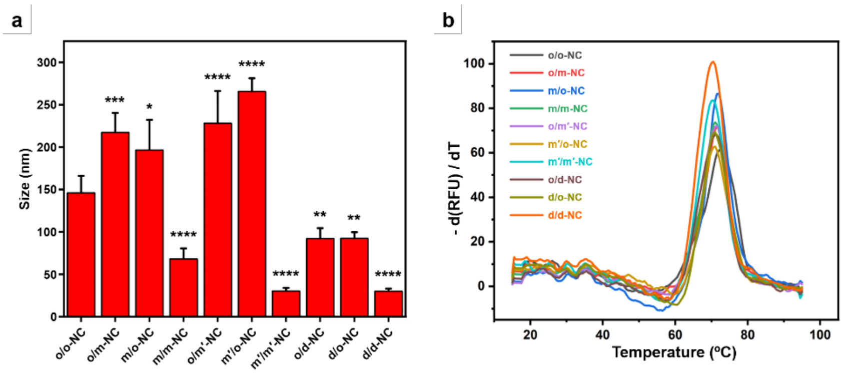

Sample	T_m
o/o-NC	72.67 (±0.24) °C
o/m-NC	71.88 (±0.22) °C
m/o-NC	71.88 (±0.22) °C
m/m-NC	71.00 (±0.00) °C
o/m'-NC	71.63 (±0.25) °C
m'/o-NC	71.00 (±0.71) °C
m'/m'-NC	70.50 (±0.58) °C
o/d-NC	71.63 (±0.22) °C
d/o-NC	70.88 (±0.22) °C
d/d-NC	70.50 (±0.00) °C

Melting temperature (T_m)

Figure S12. DLS size and thermodynamic stability of several mutant DNA NC variants. (a) Hydrodynamic diameter of several mutant DNA NC variants measured by DLS. All the DLS measurements were conducted in triplicate, and the measured values are expressed as means ± standard deviation (SD). (b) Thermodynamic stability of several mutant DNA NC variants. Melting temperature analysis of the DNA NC variants was performed by using real-time PCR. On the right is the corresponding T_m recorded from the melting curves, and the average value and standard deviation were estimated from three parallel measurements. The statistical analysis of multiple comparisons were evaluated by one-way ANOVA. *P < 0.05, **P < 0.005, ***P < 0.0005, ****P < 0.0001.

Experimental procedure:

Melting temperature (T_m) analysis was performed to study the thermodynamic stability of NC variants. DNA NC was assembled according to the experimental procedure previously described. Then, 18 μL of NC solution and 2 μL of SYBR Green I (10 ×) were added to a 0.2-mL PCR tube (Labselect, USA), followed by thoroughly mixing to allow the SYBR Green I to fully intercalate into dsDNA and fluoresce. The T_m analysis was performed on a CFX96™ real-time PCR detection system (Bio-RAD, USA), and the temperature was scanned from 10°C to 95°C at a heating rate of 0.5°C/30 s. The final data were analyzed using the software that comes with the instrument. The DLS measurement was performed according to the procedure described in Figure S1.

Discussion:

The DLS hydrodynamic diameter of mutant DNA NC variants (o/o-NC, m/o-NC, o/m-NC, m/m-NC, m'/o-NC, o/m'-NC, m'/m'-NC, d/o-NC, o/d-NC, and d/d-NC) is shown in Figure S12a. It can be noticed that the particle size distribution is basically consistent with the corresponding AFM data. In order to further verify the effect of sticky ends on the structural properties of DNA nanoclusters, the thermodynamic stability of DNA NC variants was analyzed by performing melting temperature analysis. The melting curve was generated through monitoring the fluorescence change of SYBR Green I during heating. When the temperature increases, the dsDNA-intercalated SYBR Green I is released and the fluorescence decreases, obtaining a characteristic melting profile. As shown in Figure S12b, o/o-NC does show the highest T_m value (72.67 °C), indicating the desirable thermodynamic stability. The base change, even if the mutation and deletion occur in only one sticky end, causes the decrease in T_m. Moreover, the base mutation or deletion in two sticky ends does cause the further decrease in T_m. It is also of note that too high T_m can hamper the hierarchical assembly. For example, Tetra-6GC is totally inappropriate to serve as the structural unit for the assembly of DNA NC (seen in Figures S3-S5).

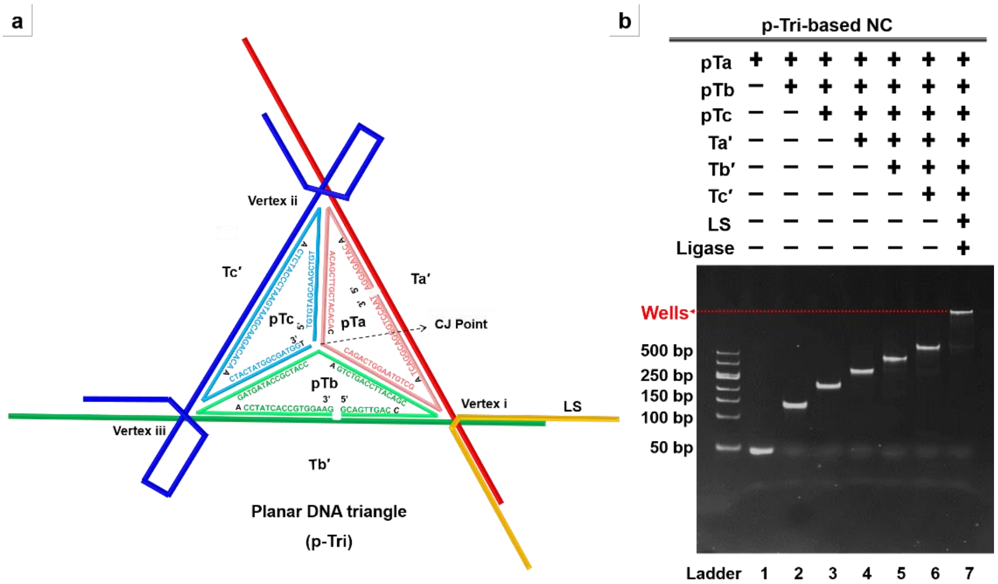

Figure S13. The dependence of DNA NC on the 3D structure of DNA tetrahedron unit. (a) Schematic diagram of planar DNA triangle (p-Tri) and the base sequence of components. Compared with the basic Tetra unit of DNA NC (Figure S1), the vertex iv is flattened by shortening the lateral edge length. (b) The step-by-step assembly of p-Tri-based NC variant (abbreviated as p-NC) ‘observed’ by nPAGE analysis. The p-Tri was assembled through the procedure described in the section of "Stepwise assembly of DNA nanocluster", but p-Ta, p-Tb and p-Tc were used instead of Ta, Tb and Tc, respectively. The red arrow is the position of gel/well interface. The nPAGE analysis and AFM measurement were performed as described in Figure S6.

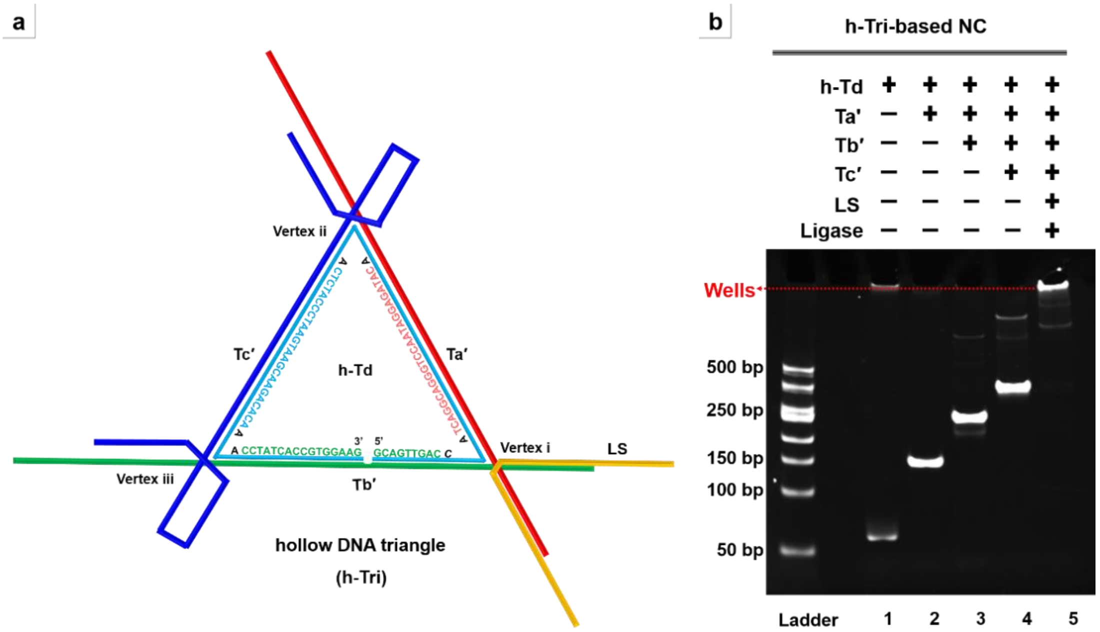

Figure S14. The assembly of DNA nanostructure consisting of common DNA triangle unit that is also called hollow DNA triangle without Y-shaped backbone in the center. (a) Schematic illustration of hollow DNA triangle (h-Tri) and base sequence of components. Compared with p-Tri represented in Figure S13, the Y-shaped backbone is removed from the center in h-Tri. (b) The step-by-step assembly of h-Tri-based nanostructure evidenced by nPAGE analysis. The red arrow is the position of gel/well interface.

Experimental procedure:

For the assembly of h-Tri DNA structure, 2.5 µL of 10 × T4 DNA ligase buffer and equal amounts (0.5 µL, 10 µM) of h-Td, Ta', Tb', Tc' and LS were added into 20.5 µL of H_2O and mixed well. After heating at 90°C for 5 min, the mixture was placed in a dry bath incubator and gradually cooled down to room temperature. Subsequently, T4 DNA ligase (0.5 µL, 400 U/µL) was added and incubated at 16°C overnight to allow the formation of corresponding NC variant (called h-NC). The procedures for nPAGE analysis and AFM characterization are consistent with Figure S6.

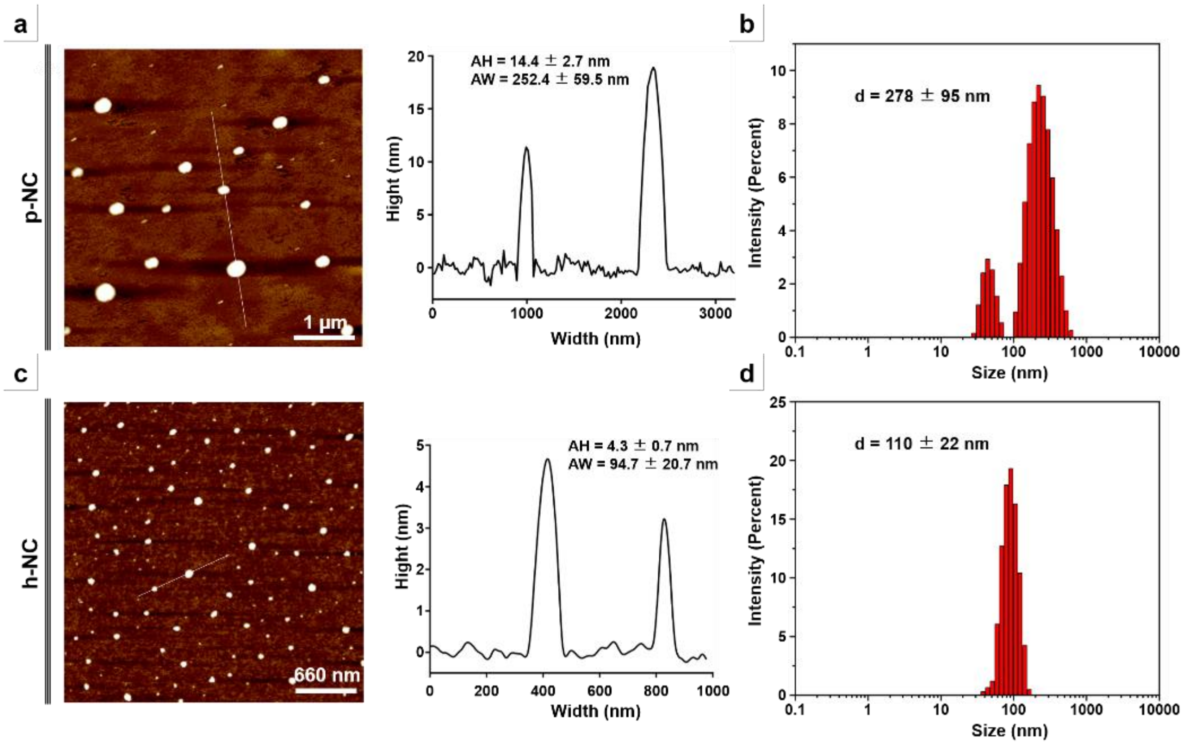

Figure S15. AFM and DLS characterization of p-NC and h-NC. (a) AFM image of p-NC assembled from p-Tri unit and cross-section profile of AFM image along the white line. (b) Hydrodynamic diameter of p-NC assembled from p-Tri unit measured by DLS. (c) AFM image of h-NC assembled from h-Tri unit and cross-section profile of AFM image along the white line. (d) Hydrodynamic diameter of h-NC assembled from h-Tri unit measured by DLS. AH and AW denote the average height and average width (n = 20), respectively. All the DLS measurements were conducted in triplicate, and the values are expressed as means ± standard deviation (SD).

Discussion for Figures S13-S15:

In addition to the exploration of the relationship between the mutant sticky ends of structural unit and the assembly of NC objects, the influence of 3D structure of tetrahedron unit on the assembly of NC was also studied. For this purpose, the planar DNA triangle (p-Tri) with a Y-shaped backbone in the center and hollow DNA triangle (h-Tri) without the Y-shaped backbone were separately used as the structural unit for the assembly of NC where all base sequences in three edges of bottom face and four connection arms, including four sticky ends, remain unchanged. Their structural diagrams are illustrated in Figure S13a and Figure S14a, respectively. The former was designed by shortening the length of lateral edges to flatten the out-pointing vertex-iv into a central junction point (CJ point) with three branches. Namely, the three lateral 'edges' and vertex-iv are condensed within a triangular plane (i.e., bottom face). In comparison, the latter is designed by further removing the Y-shaped backbone from the center of DNA triangle via substituting p-Ta, p-Tb, and p-Tc with a long h-Td during the assembly process. The p-Tri and h-Tri have the same edges and connection arms as the bottom face of o/o-Tetra. The corresponding assembled products are called p-NC and h-NC, respectively.

As shown in Figure S13b, the nPAGE image shows the distinct difference in band mobility between adjacent lanes, indicating the stepwise hybridization during the assembly of p-NC. However, AFM measurement (Figure S15a) shows that p-NCs are heterogeneous in size. Average width (AW) and average height (AH) are 252.4 ± 59 nm and 14.4 ± 2.7 nm, respectively. Compared with the size of o/o-NC (Figure 1), p-NC has the slightly larger AW and much smaller AH, demonstrating the easy collapse of assembled structure. Moreover, the relative standard deviation (RSD) of AW is more than 23%, implying the

simultaneous formation of small or large products. Figure S15b shows the corresponding DLS data. The sizes of assembled products are indeed distributed into two different regions, and the average particle size is 278 ± 95 nm (the RSD of more than 34%).

Using the same methods (i.e., electrophoretic analysis and AFM measurement), the assembly of h-NC was explored. Although the nPAGE analysis shows that h-NC can be assembled from DNA components without obvious by-products (Figure S14b), the assembled products are distinctly different from o/o-NC. As shown in Figure S15c, the AW and AH are 94.7 ± 20.7 nm and 4.3 ± 0.7 nm, respectively, indicating that h-NC is much smaller than o/o-NC and shows the heterogeneous distribution in size. Since the diameter of double-helical DNA is about 2 nm,[37, 38] the thickness of h-NC is only equal to two layers of h-Tetras, indicating the assembly failure. Similar to p-NC, the relative standard deviation (RSD) of AW is 22%, indicating the simultaneous generation of h-NCs with various sizes. In Figure S15d, the corresponding DLS data show that the average particle size is 110 ± 22 nm (RSD of more than 20%).

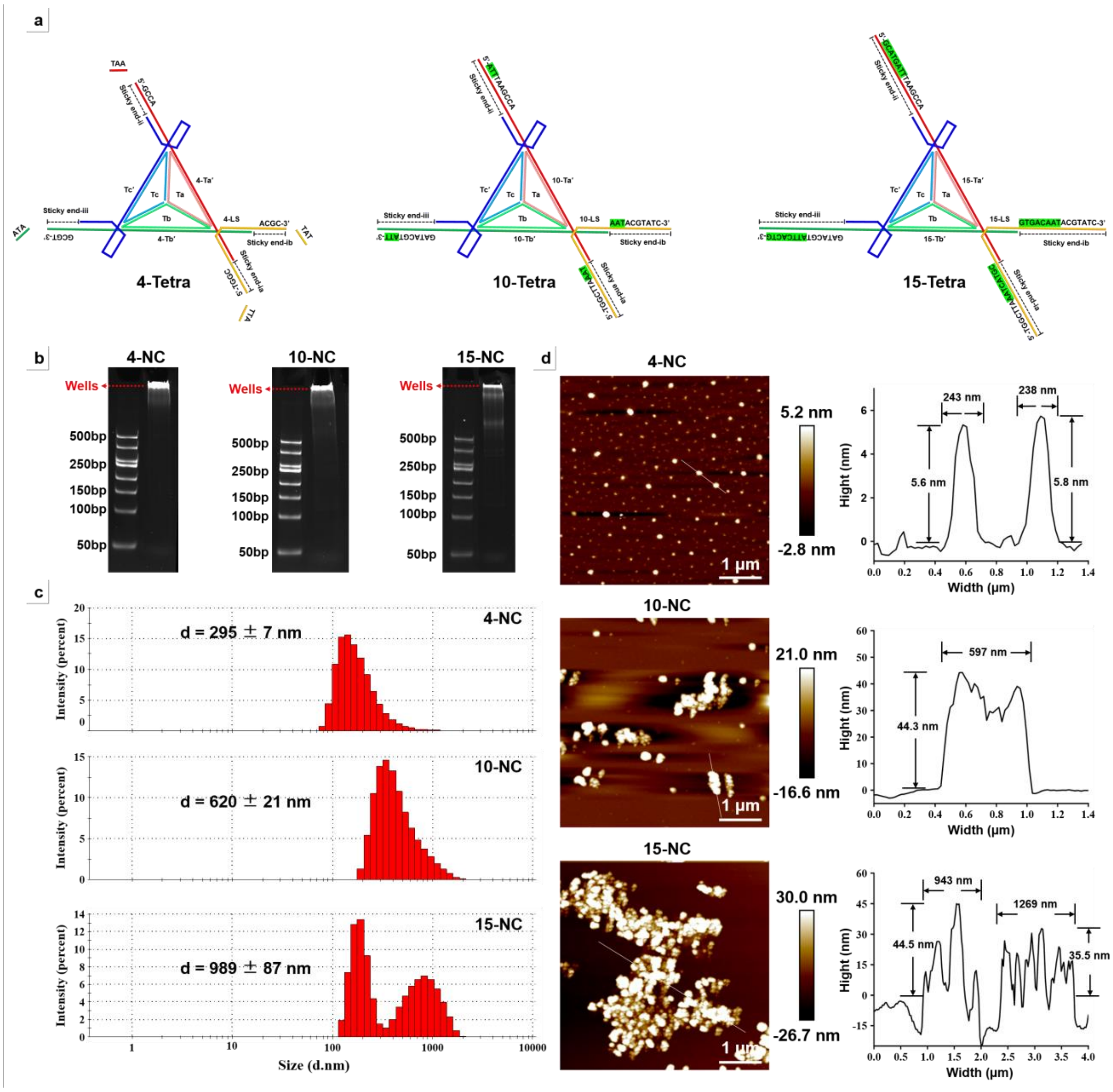

Figure S16. The effect of base number in sticky ends on the assembly of nanoclusters. (a) Structural representation of DNA Tetra unit with different base numbers (4, 10 and 15 bases) in the sticky ends that are called 4-Tetra, 10-Tetra and 15-Tetra, respectively. Compared with o/o-Tetra, the stray fragments denote the domains deteted from the sticky ends, while the fragments with green background are the insertion bases to extend the sticky ends. (b) Native PAGE (nPAGE, 6%) analysis of corresponding assembled NC objects (called N-NC). 4-NC, 10-NC and 15-NC represents the nanoobjects that are assembled from basic structural units 4-Tetra, 10-Tetra and 15-Tetra, respectively. The red arrow is the position of gel/well interface. (c) Hydrodynamic diameter of N-NC measured by DLS and (d) corresponding AFM data. All the DLS measurements were conducted in triplicate, and the values are expressed as means ± standard deviation (SD). For the AFM images, Scale bar is 1 μm.

Experimental procedure:

The N-NC object was assembled as described in the section of "Stepwise assembly of DNA nanocluster". Then, the assembled N-NC sample was 10 times diluted with 1 × T4 DNA ligase buffer. The DLS measurement was carried out according to the method described in the section of "Dynamic light scattering measurement", while AFM measurement was performed according to the procedure described in the section of "AFM characterization of DNA nanocluster (NC)". All the measurements were conducted in triplicate, and the values are expressed as means ± standard deviation (SD).

Discussion:

The base number in the sticky ends was changed to further explore the effect of sticky ends on the assembly of DNA nanoclusters, and the experimental results are shown in Figure S16. One can notice that, compared with o/o-NC (AH=30.9 nm, AW=136.7 nm measurd by AFM; DLS size of 146, Figure S1), the

DLS size and AFM width of all the N-NCs are much larger, indicating the occurrence of the assembly from DNA building blocks. However, the height (less than 5.8 nm) of 4-NC is only slightly higher than normal DNA tetrahedron (AH=2.6 nm, Figure S17), indicating the substantail collapse into two layers of basic structural units and the assembly failure of expected three-dimensional structure. Although the height of 10-NC and 15-NS obviously increases, their size (620 nm for the former and 989 nm for the latter) is too large to be effectively internalized by most cancer cells.[39] This is because the structural units strongly aggregate (seen in corresponding AFM images) owing to the stable hybridiztion between sticky ends. These experimental results demonstrate that the rational design of sticky ends is more conducive to the assembly of desirable DNA NC.

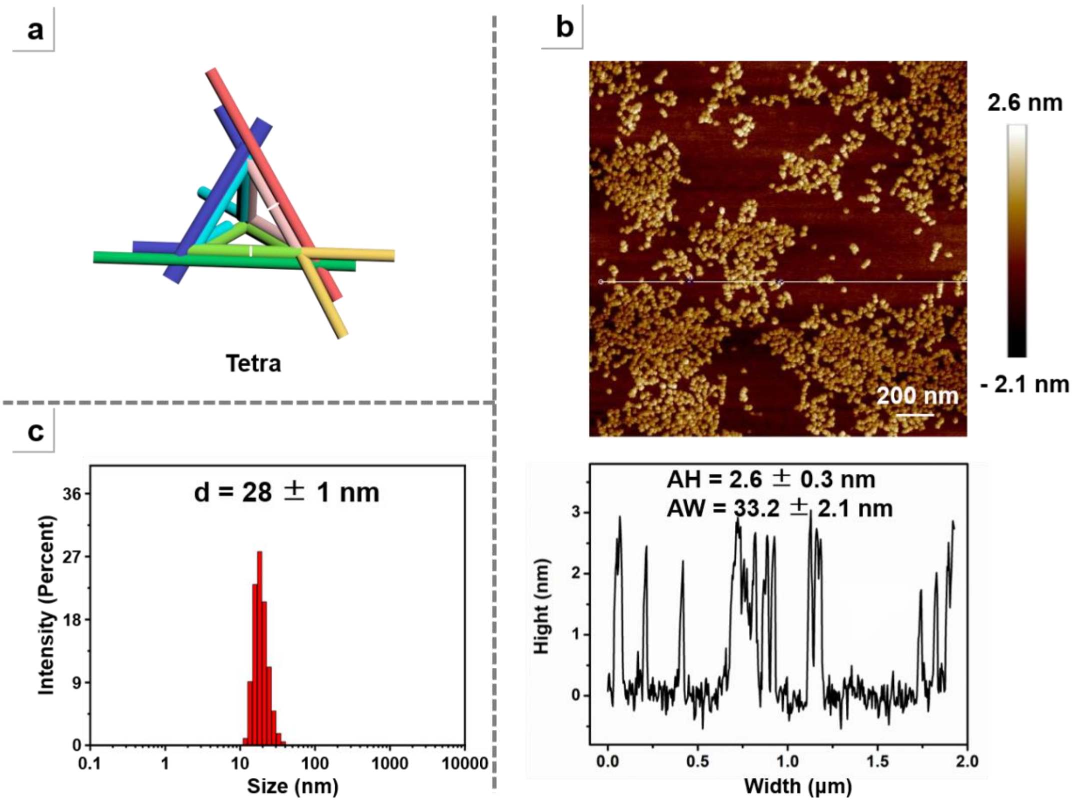

Figure S17. Characterization of DNA tetrahedron unit. (a) Schematic illustration of DNA tetrahedron unit. (b) AFM image and cross-section analysis of DNA tetrahedron units. (c) DLS characterization of DNA tetrahedron units. AH and AW denote the average height and average width (n = 20), respectively.

Experimental procedure:

DNA tetrahedron was assembled by simple annealing of seven DNA components. In short, 2.5 µL of 10 × T4 DNA ligase buffer and equal amounts (0.5 µL, 10 µM) of Ta, Tb, e-Tc, Ta', Tb', Tc' and LS DNA strands were added into 19 µL of H_2O and mixed well. After heating at 90°C for 5 min, the solution was allowed to gradually cool down to room temperature, forming the DNA tetrahedron. The concentration of DNA tetrahedron unit was 200 nM. DLS measurement and AFM scanning were conducted as described in Figure S1.

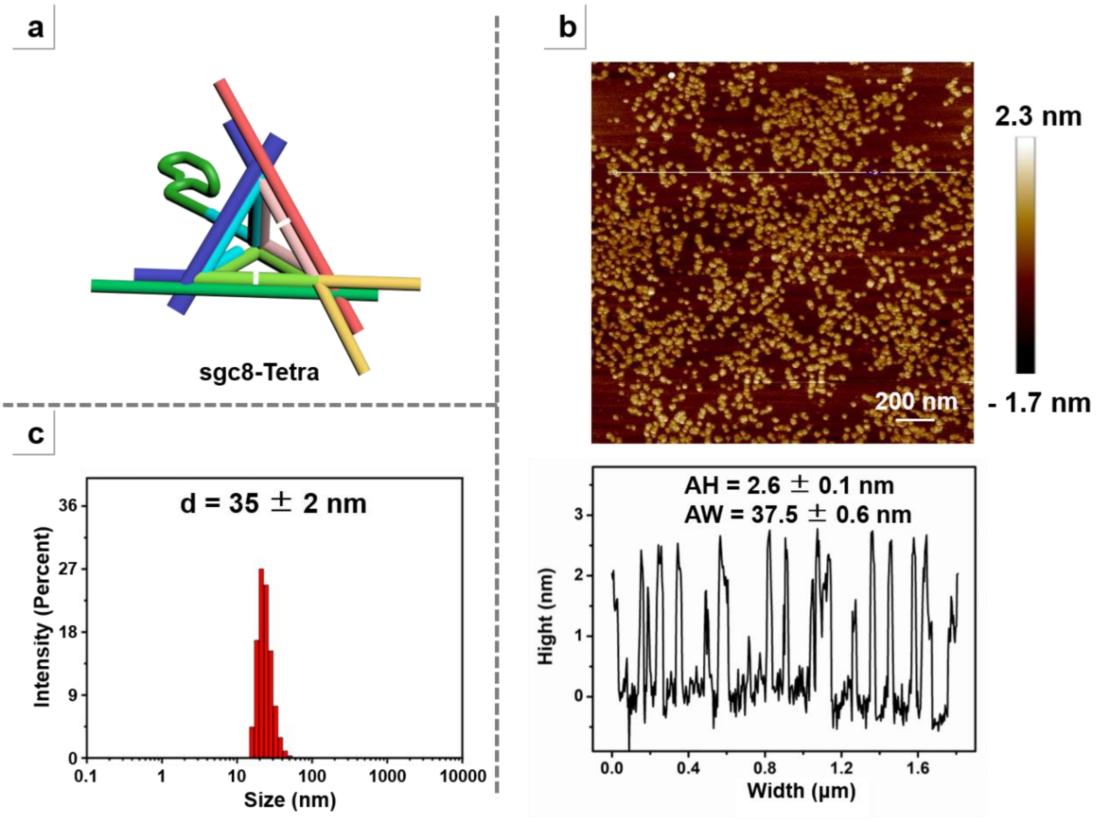

Figure S18. Characterization of DNA tetrahedron unit hybridized with sgc8 aptamer (sgc8-Tetra). (a) Schematic illustration of sgc8-Tetra unit. (b) AFM image of sgc8-Tetra unit, accompanied by the cross-section analysis along the while line. (c) DLS measurement of sgc8-Tetra. The first several experimental steps for the assembly are the same as Figure S17 and finally T4 DNA ligase was added to seal the nicks to promote the hierarchical assembly. The details are shown in the section of "Stepwise assembly of DNA nanocluster". DLS measurement was conducted as described in the section of "Dynamic light scattering measurement", while the AFM scanning was performed as shown in the section of "AFM characterization of DNA nanocluster (NC)".

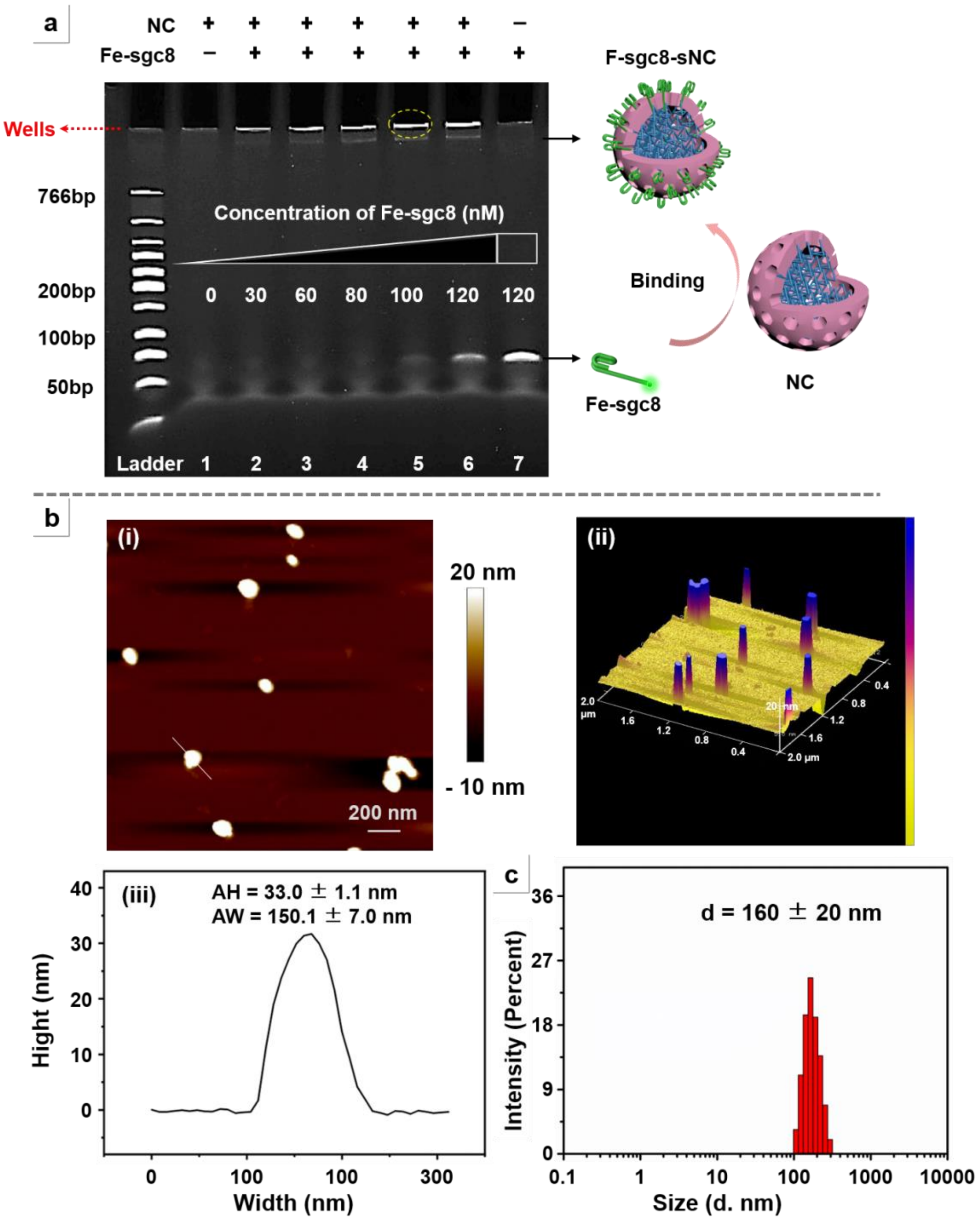

Figure S19. Characterization of aptamer-sheathed nanocluster, sgc8-sNC. (a) Hybridization of e-sgc8 to basic DNA NC. The aptamer concentration for the preparation of sgc8-sNC was optimized by changing the concentration of fluorescently modified e-sgc8 (Fe-sgc8) but keeping a constant concentration of DNA NC. The nPAGE analysis was performed to estimate the residual amount of Fe-sgc8. Lane 1 represents the pre-constructed NC serving as the control without Fe-sgc8; Lane 2-6 represents the NC in the presence of different amounts of Fe-sgc8. Lane 7 indicates Fe-sgc8. In the assembly solution, except for Fe-sgc8, the concentration of building blocks is 200 nM. The working solution is 1 × T4 DNA ligase buffer. The red arrow is the position of gel/well interface. The experimental procedure is described in the section of “Optimization of aptamer concentration for the assembly of sgc8-sNC materials”. (b) AFM characterization of sgc8-sNC. (i) AFM image, (ii) The three-dimensional (3D) pattern, (iii) The cross-section profile of sgc8-sNC along the white line. (c) DLS characterization of sgc8-sNC. The assembly of sgc8-sNC is described in the section of “Stepwise assembly of DNA nanocluster”, while DLS measurement was performed as described in the section of "Dynamic light scattering measurement" and the AFM scanning was carried according to the procedure described in the section of "AFM characterization of DNA nanocluster (NC)".

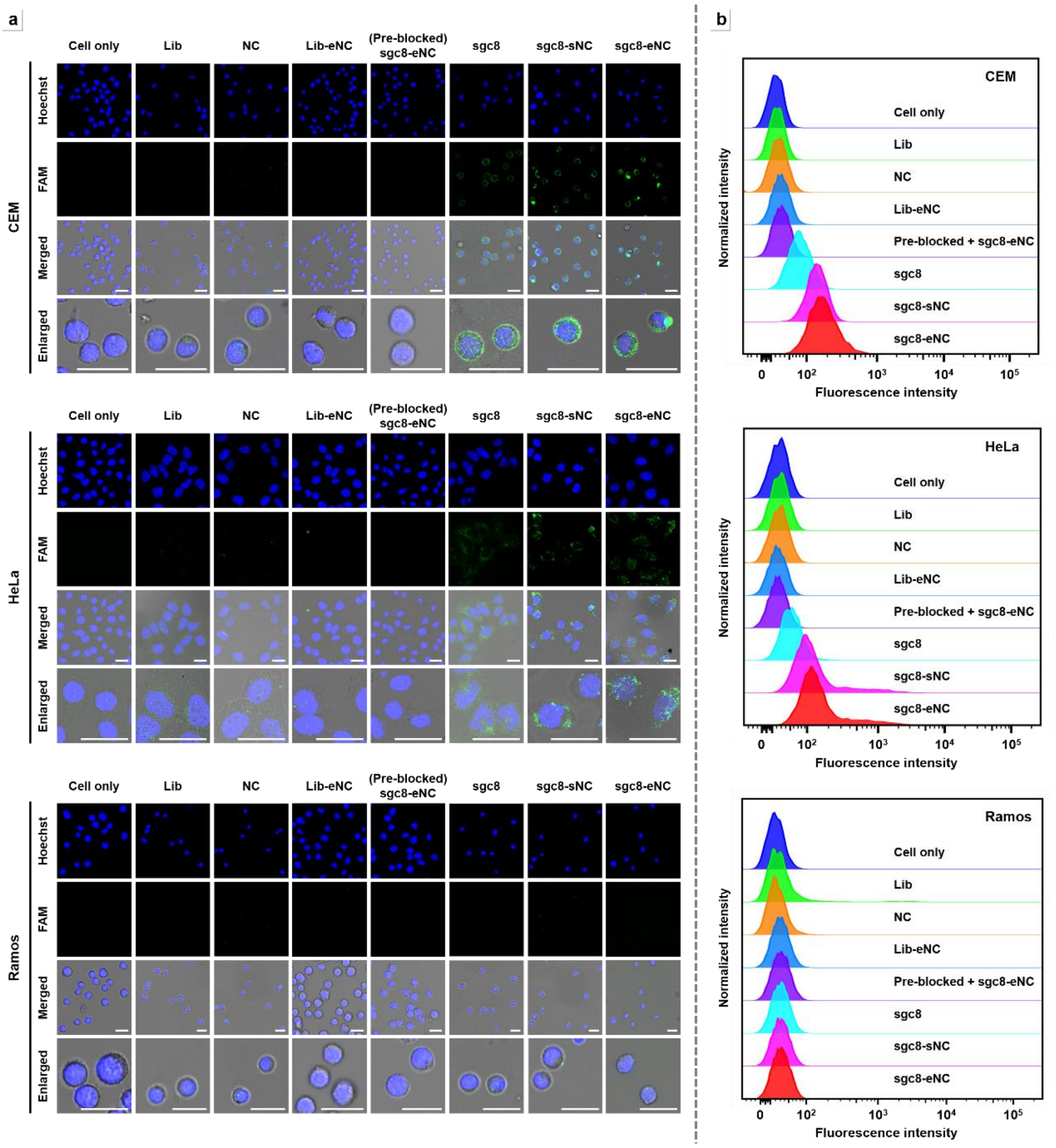

Figure S20. **Specific recognition of sgc8-eNC toward target cancer cells.** (a) Confocal laser scanning microscope (CLSM) images of CEM, HeLa cells and nontarget Ramos cells incubated with sgc8-eNC and the counterpart nanostructures. To emit the fluorescence signal, Tb'-FAM was used for the assembly of NCs (e.g., sgc8-eNC) where other components have no any fluorophore, while F-Lib and F-sgc8 were employed in Lib and sgc8 groups, respectively. The group of Pre-blocked + sgc8-eNC indicates that the receptors on cell surface were pre-blocked with free sgc8 before incubation with sgc8-eNC. Blue fluorescence originates from Hoechst 33342 stained nuclei, while green fluorescent represents FAM-labeled DNA nanostructures internalized into the cells. Scale bar = 25 μm. (b) The corresponding flow cytometric analyses of CEM, HeLa and Ramos cells. The experimental procedure is shown in the section of "Specific recognition of target cancer cells". Scale bar = 20 μm.

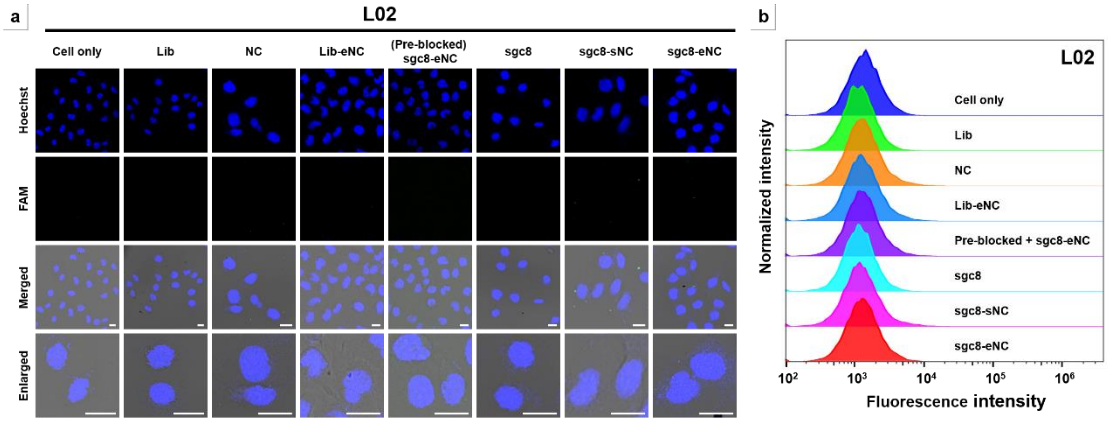

Figure S21. No cross-binding of sgc8-eNC to normal cells. (a) CLSM images of L02 cells incubated with different DNA nanostructures where Tb'-FAM was instead used to emit the fluorescence signal. Blue fluorescence originates from Hoechst 33342 stained nuclei, while green fluorescent represents FAM-label DNA nanostructures internalized into cells. (b) Flow cytometric analysis of corresponding L02 cells. The experimental procedure is shown in the section of "Specific recognition of target cancer cells". Scale bar = 20 μm.

Discussion:

L02 was used as a normal model cell to further verify the selective recognition of sgc8-eNC. As shown in Figure S21a, no detectable difference in fluorescence signal between Lib group and sgc8 group is observed because L02 cells are PTK7-negative cells.[40-42] Similarly, no fluorescence shift is observed in other experimental groups, including sgc8-eNC, indicating no cellular uptake. The flow cytometry analysis shows the consistent experimental results (Figure S21b), demonstrating that sgc8-eNC is not internalized by healthy cells.

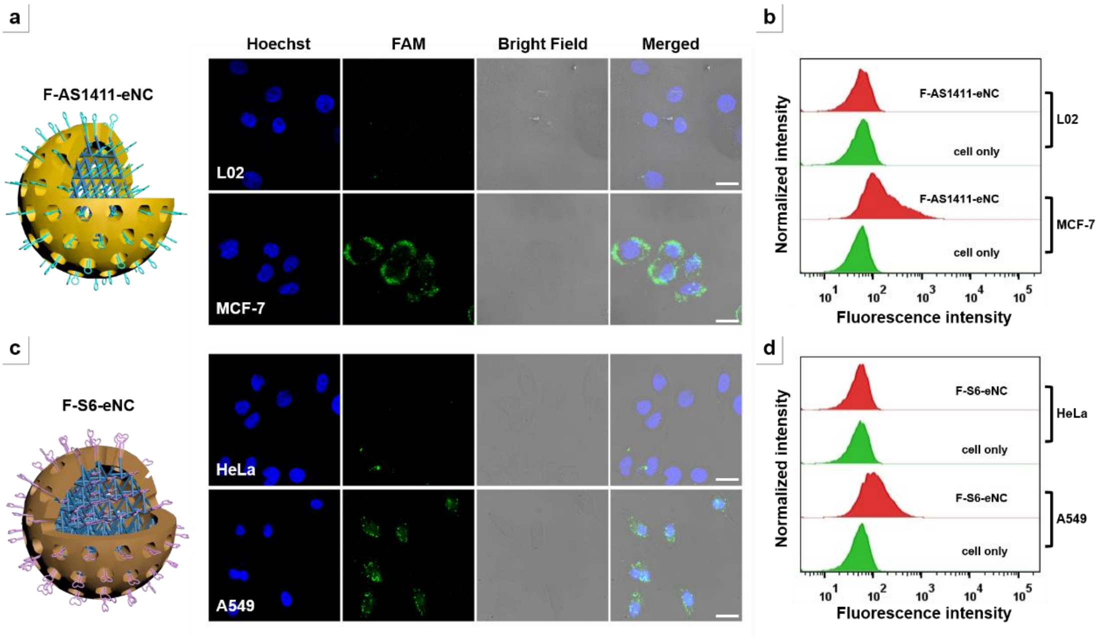

Figure S22. The universality of aptamer-conjugated DNA nanoclusters for specific recognition of cancerous cells. (a) CLSM images and (b) flow cytometric quantification of target MCF-7 cells and control negative L02 cells incubated with F-AS1411-eNC. (c) CLSM images and (d) flow cytometric quantification of target A549 cells and control negative HeLa cells incubated with F-S6-eNC. Scale bar = 20 μm.

Experimental procedure:

Firstly, F-AS1411-eNC and F-S6-eNC were separately assembled through the previously described procedure in the section of "Stepwise assembly of DNA nanocluster", but the targeting ligands, AS1411 and S6, were used instead of sgc8, respectively. Moreover, to make DNA NC fluoresce, the Tb' was substituted with Tb'-FAM. Then, selective recognition ability of the two aptamer-conjugated eNCs was explored as described in the section of "Specific recognition of target cancer cells".

Discussion:

In order to study the universality of aptamer-embedded DNA nanocluster, the sgc8 aptamer was substituted with the AS1411 that specifically recognizes human breast cancer cells (MCF-7 cells) [43] or with S6 molecule that specifically binds to human lung adenocarcinoma cells (A549 cells) [44] for the assembly of two new nanoclusters: F-AS1411-eNC and F-S6-eNC. The schematic diagrams are shown in the left half of Figures S22a and S22c, respectively. It can be seen from CLSM images (Figure S22a) that there is a high fluorescence signal in MCF-7 cells, while the control group L02 cells do not fluoresce. The flow cytometric data are consistent with the CLSM images (Figure S22b), proving that F-AS1411-eNC can specifically target MCF-7 cells. Similarly, both CLSM images and flow analysis demonstrate that FAM-S6-eNC has specific binding ability toward target A549 cells against negative non-target HeLa cells (Figure S22c and S22d). In this study, sgc8–eNC was employed for subsequent experiments.

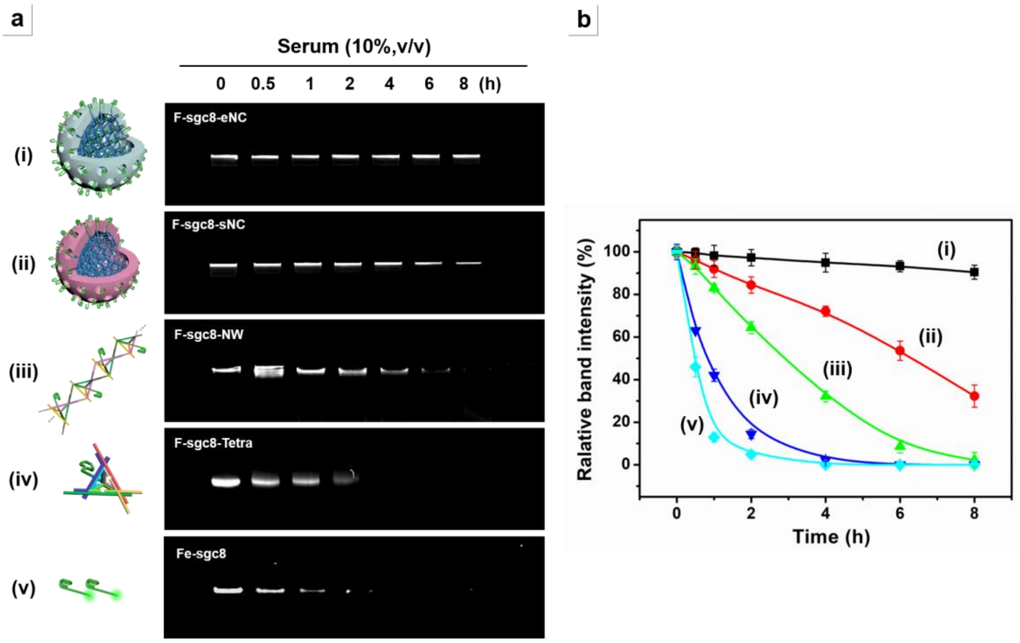

Figure S23. Serum stability of DNA NCs. (a) nPAGE images of (i) FAM-sgc8-eNC, (ii) FAM-sgc8-sNC, (iii) FAM-sgc8-NW, (iv) FAM-sgc8-Tetra and (v) Fe-sgc8. FAM was attached onto sgc8 strand in each nanoassembly. (b) Quantitative evaluation of residual DNA strands recorded from (a). The error bars represent the standard deviation (SD, n = 3). Except that Fe-sgc8 concentration for the assembly of Fe-sgc8-sNC is 100 nM (seen in Figure S19), the concentration of DNA components involved is 200 nM. The DNA nanostructures were separately incubated in 10% fresh mouse serum for different time periods and then subjected to nPAGE analysis.

Experimental procedure:

For the DNA FAM-sgc8-NW, the assembly was performed according to the experimental procedure adopted by Gao and her colleagues,[8] and the same DNA components were used. Specifically, equal amounts (0.5 µL,10 µM) of DNA components were added into 19 µL of H_2O and 2.5 µL of 10 × TAE/Mg^{2+} buffer (200 mM acetic acid, 400 mM Tris, 125 mM $MgCl_2$ and 20 mM EDTA, pH = 7.6) and mixed thoroughly. The resulting solution was heated at 90°C for 5 min and then slowly cooled down to 25°C by incubating in a dry bath incubator. Afterward, the linker strand (0.5 µL, 10 µM) was added and incubated at 25°C for 2 h. Subsequently, the Fe-sgc8 (0.5 µL, 10 µM) was added and allowed to hybridize for 2 h at 25°C, forming FAM-sgc8-NW (the final concentration of DNA tetrahedron unit, 200 nM). The details on the experimental procedure for the assembly of other DNA nanostructures and exploration of the serum stability of all DNA nanostructures are described in the section of “Evaluation of serum stability”.

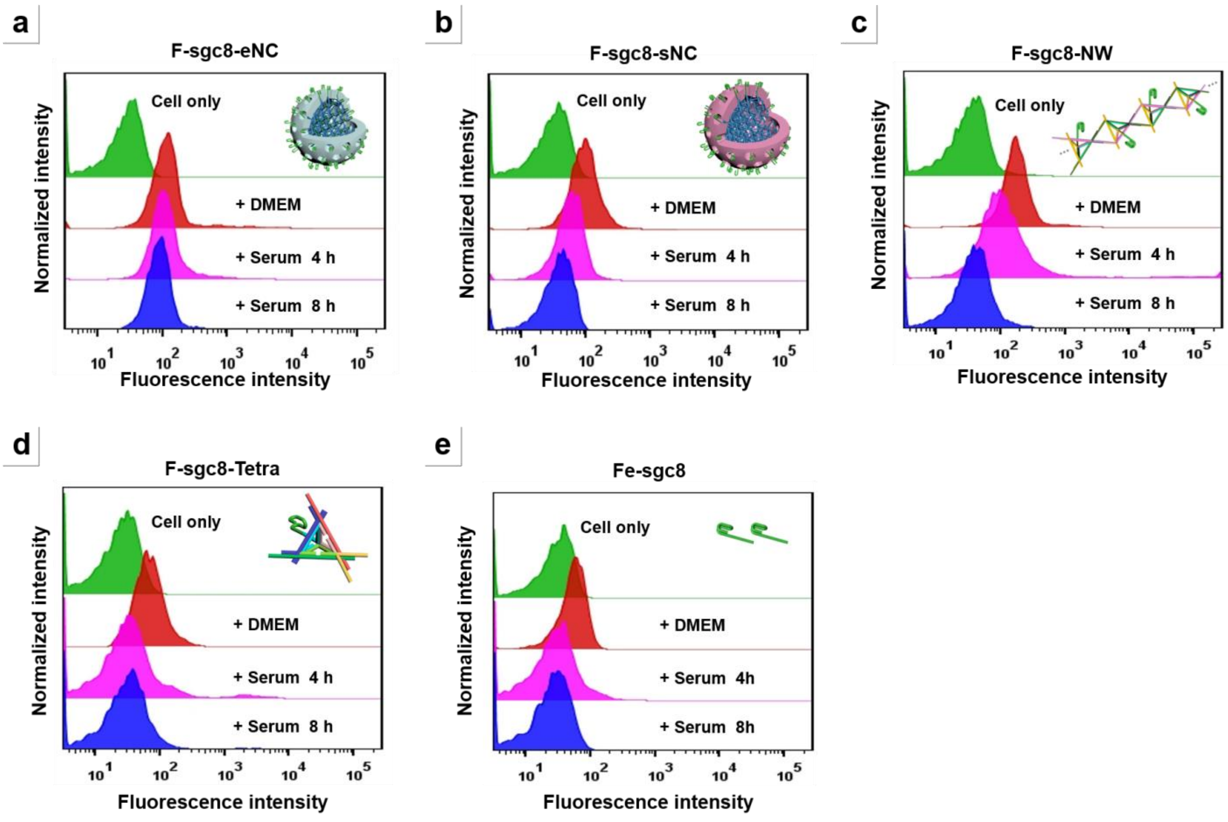

Figure S24. Cellular internalization of aptamer-conjugated DNA nanomaterials pre-subjected to the serum degradation into target HeLa cells, which was evaluated by the flow cytometry analysis: FAM-sgc8-eNC (a), FAM-sgc8-sNC (b), FAM-sgc8-NW (c), FAM-sgc8-Tetra (d) and Fe-sgc8 (e). The five DNA nanostructures or molecule were pre-treated with 10% fresh mouse serum for 4 or 8 h. The fluorescence signal of HeLa cells treated with corresponding intact DNA nanostructures was used as positive control (red histogram), while fluorescence signal from cells only without any treatment was employed as negative control (green histogram).

Experimental procedure:

Firstly, 1×10^5 HeLa cells were added to a 24-well plastic plate with a round coverslip and cultured in complete Dulbecco's Modified Eagle Medium (DMEM) (10% FBS and 1% penicillin-streptomycin) in a humidified 5% CO_2 atmosphere overnight at 37°C. Then, 3 µL of fresh mouse serum and 1 × T4 buffer (2 µL) were mixed with DNA nanostructure of interest (25 µL) and incubated at 37°C for 4 h or 8 h where the final serum concentration is 10%. After washing the cells three times with PBS buffer, 30 µL of pre-treated DNA nanostructures and 170 µL of DMEM were added to the 24-well plastic plate and cultured in a humidified atmosphere of 5% CO_2 at 37°C for 2 h. The resulting HeLa cells were digested with trypsin without EDTA and resuspended in 1 mL of PBS. Flow cytometry analysis was conducted by counting at least 10000 cells on a BD FACSAria™ III flow cytometer (BD Biosciences, USA).

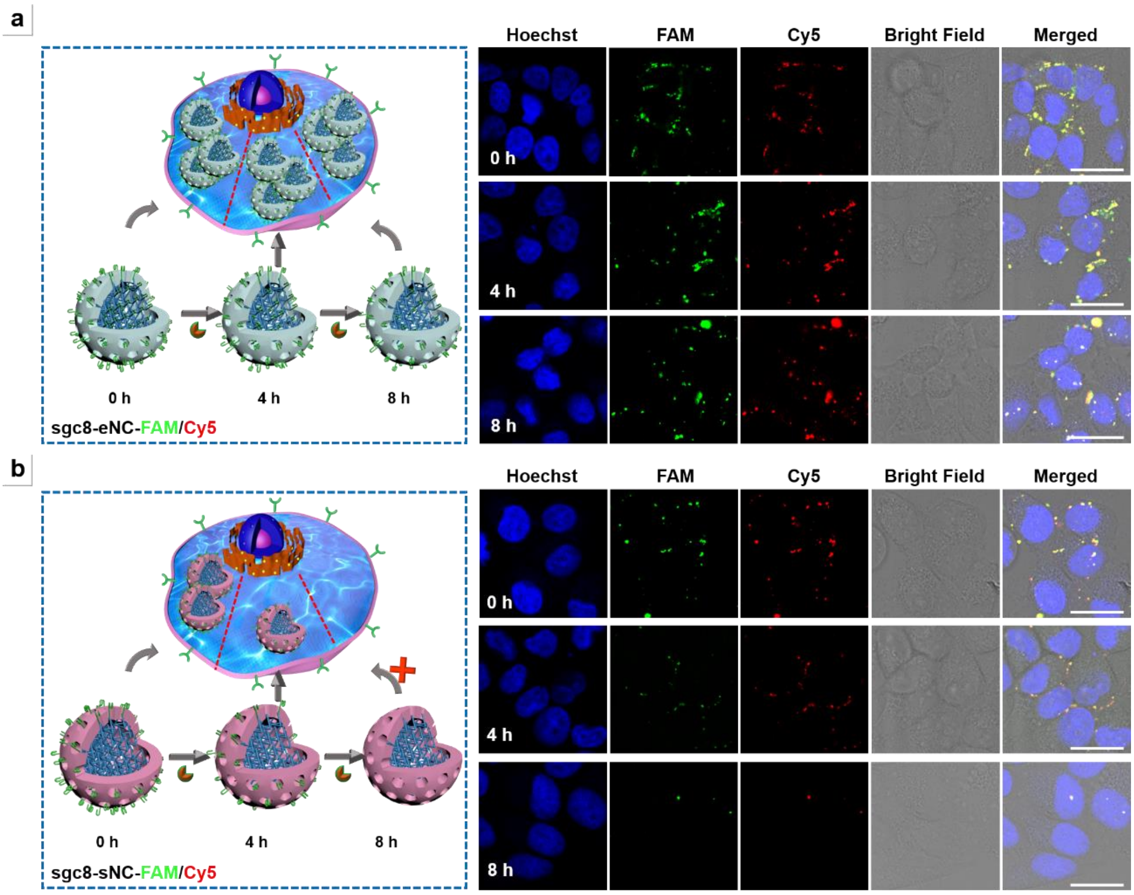

Figure S25. Serum stability analysis of cell-targeting ability of aptamer-conjugated DNA NC evaluated by dual-color fluorescence imaging at the cellular level. (a) CLSM images of HeLa cells treated with sgc8-eNC exposed to fresh mouse serum for different time periods (0 h, 4 h and 8 h), (b) the same as panel a but sgc8-sNC counterpart was instead used. The left panel is the schematic diagram of experimental process. To emit the dual-color fluorescence signal, Tb'-Cy5 and Fe-sgc8 were used for the assembly of aptamer-conjugated DNA NC. The sgc8-incorporated NCs were pre-treated with serum according to the same procedure described in Figure S23. Other experimental details, for example, staining cell nuclei with Hoechst 33342, are seen in the section of “Specific recognition of target cancer cells”. Scale bar = 20 µm.

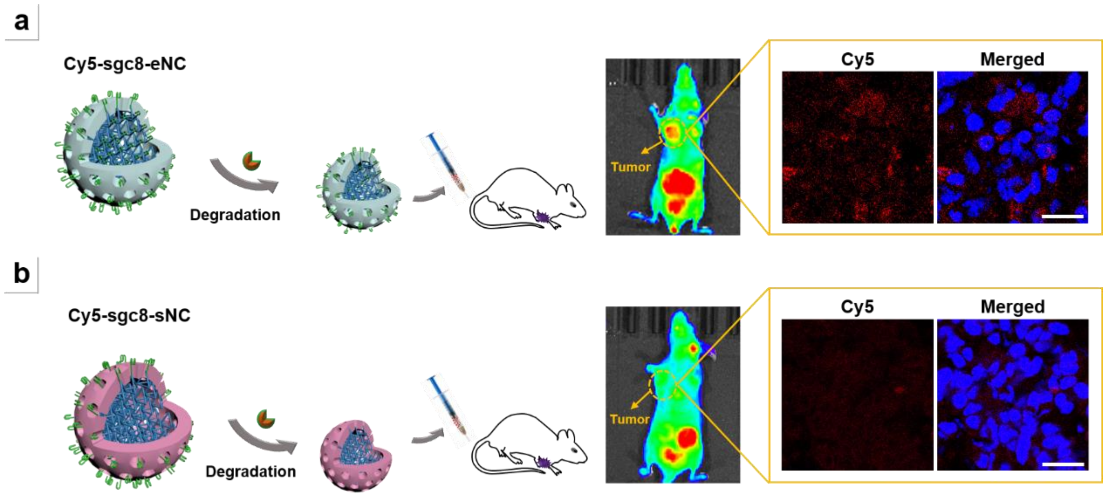

Figure S26. Serum stability analysis of cell-targeting ability of aptamer-conjugated DNA NC by whole-animal *in vivo* imaging of tumor-bearing mice. (a) *In vivo* image of whole body systemically injected with Cy5-sgc8-eNC and CLSM image of frozen slices of tumor tissues. Left panel: Schematic diagram of serum treatment of Cy5-sgc8-eNC and subsequent administration into tumor-bearing animal model. (b) The same as (a) but Cy5-sgc8-sNC counterpart was instead explored. Scale bar = 20 μm.

Experimental procedure:

First, the HeLa tumor-bearing mouse model was established according to the experimental procedure described in the section of "*In vivo* mouse xenograft model". When the tumor volume reaches 400 mm^3, the mouse was used to study the *in vivo* distribution of DNA NCs. Specifically, firstly, to emit the fluorescence signal, Tb'-Cy5 was used instead of Tb' for the assembly of DNA nanoclusters (Cy5-sgc8-eNC and Cy5-sgc8-sNC), followed by the concentration treatment (final concentration of DNA tetrahedron units, about 500 nM). Then, fresh mouse serum (final concentration, 10%) was added and mixed thoroughly, followed by incubation at 37°C for 4 h. Afterward, DNA nanoclusters were administrated through the tail vein injection. The whole-animal *in vivo* imaging was performed at 4 h post-administration. Next, the nude mice were anesthetized and sacrificed, the tumor tissues were collected for the preparation of frozen section. Tumor tissues were cut into 7-μm thickness of slices and washed twice with PBS. After staining the tissue slice with Hoechst 33342 at room temperature for 15 min, 2 μL of Antifade Mounting Medium was added dropwise onto the center of cover slip and placed upside down on a microscope slide. The slices were imaged on CLSM system to estimate the accumulation of DNA assemblies within the tissues.

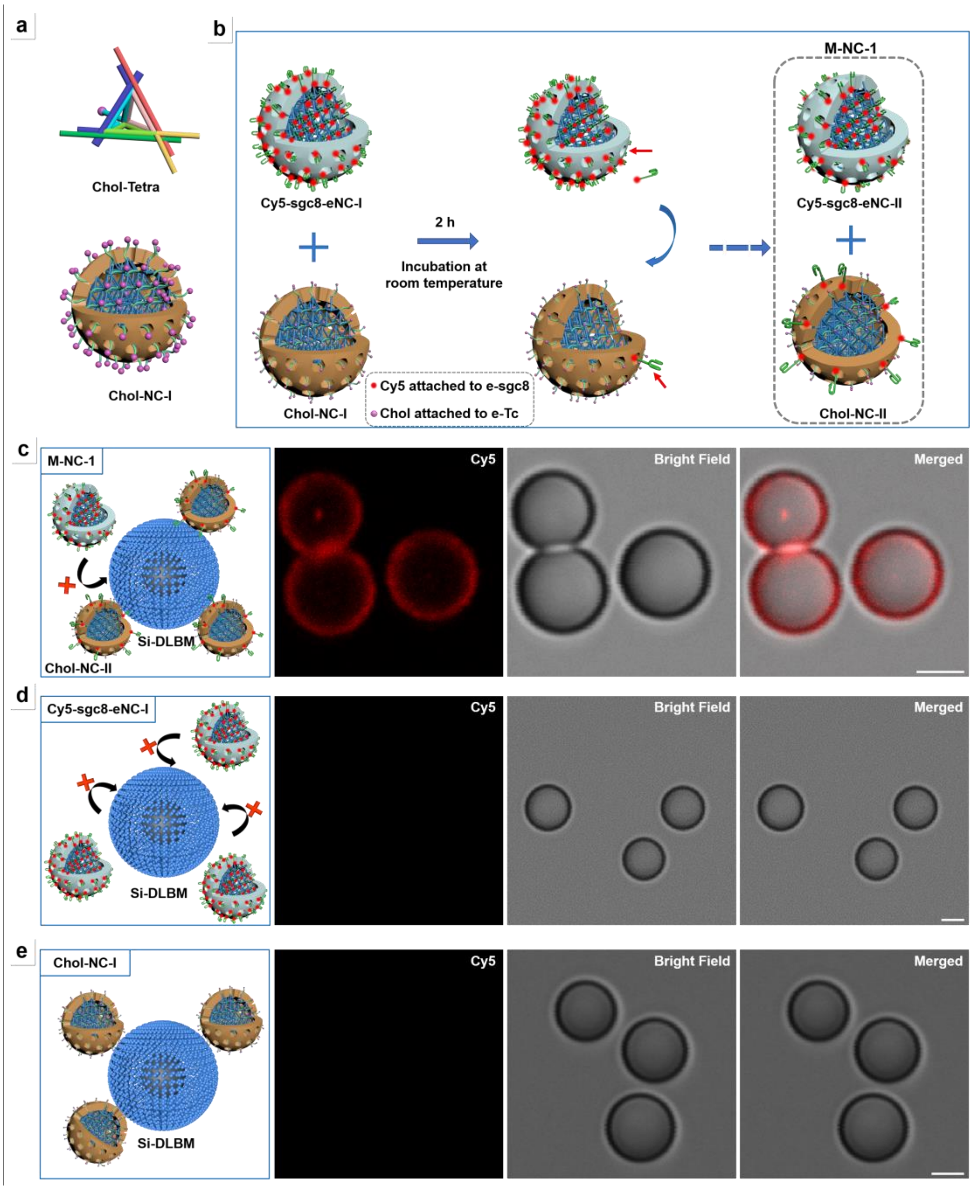

Figure S27. Single-color fluorescence imaging to validate the transfer of aptamers between different DNA NC nanostructures. (a) Structural representation of DNA NC with cholesterol group (called Chol-NC-I) and its tetrahedron unit consisting of Ta, Tb, Chol-e-Tc, Ta', Tb', Tc' and LS. (b) Schematic diagram of the transfer of Cy5e-sgc8 from Cy5-sgc8-eNC-I to Chol-NC-I, generating M-NC-1 containing Cy5-sgc8-eNC-II and Chol-NC-II capable of fluorescing. (c) CLSM images of Si-DLBM exposed to M-NC-1 (prepared by incubating Cy5-sgc8-eNC-I with Chol-NC-I for 2 h). (d) CLSM images of Si-DLBM exposed to only Cy5-sgc8-eNC-I. (e) CLSM images of Si-DLBM exposed to only Chol-NC-I. Scale bar = 2.5 μm.

Experimental procedure:

The Chol-NC-I and Cy5-sgc8-eNC-I were assembled through the previously described procedure (seen in "Stepwise assembly of DNA nanocluster"), but Chol-e-Tc was used instead of Tc for the former assembly and Cy5e-sgc8 was substituted for e-sgc8 for the latter assembly. Then, Cy5-sgc8-eNC-I (50 μL) was mixed with Chol-NC-I (50 μL) and incubated at room temperature for 2 h. After 5 μL of pre-synthesized Si-DLBM (seen in the section of "Si-DLBM construction") was added and mixed, the resulting solution was incubated at room temperature for 30 min, followed by centrifugation at 10000 RPM for 10 min. The supernatant was removed, and the precipitates were washed twice with 50 μL of PBS and resuspended in 20 μL of PBS. Finally, the resuspension (10 μL) was pipetted onto a confocal small dish, and the Cy5-based CLSM imaging was performed with a 20 × water objective at the excitation wavelength of 645 nm and emission wavelength of 670 nm.

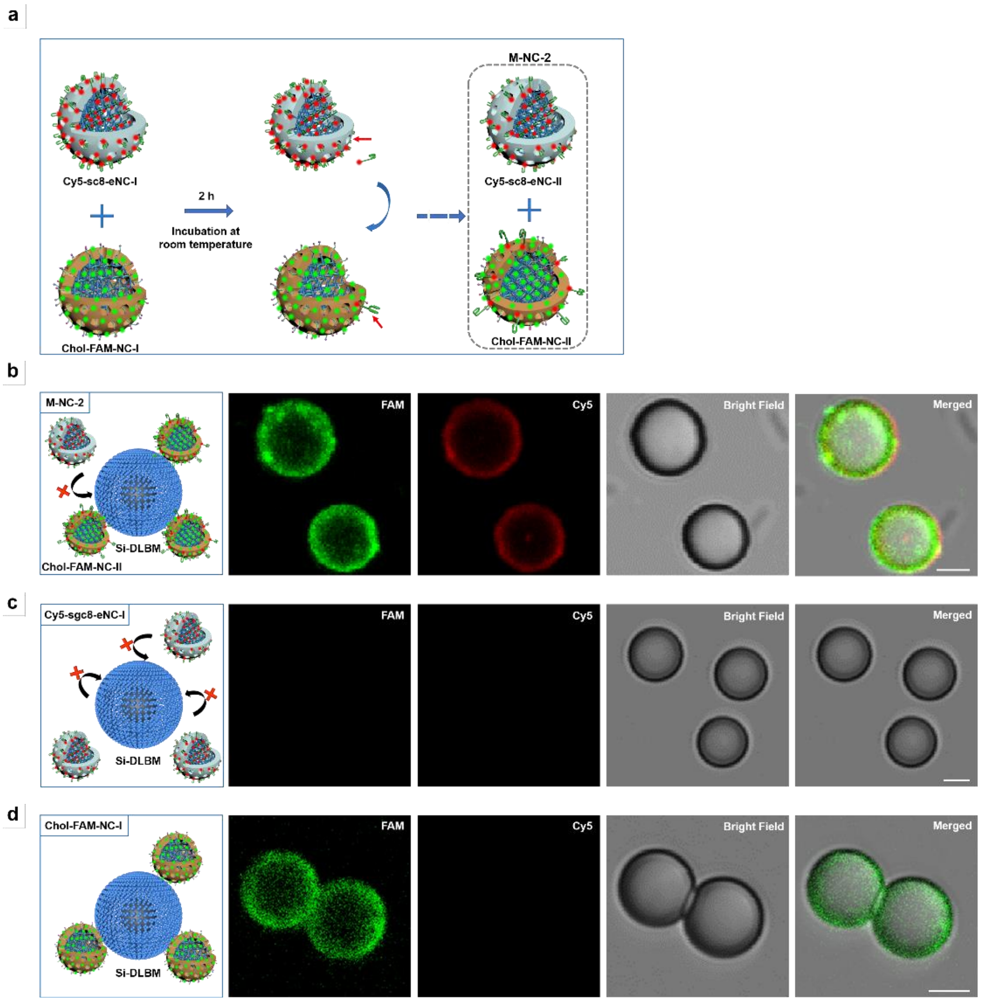

Figure S28. Dual-color fluorescence imaging to validate the transfer of aptamers between different DNA NC nanostructures. (a) Schematic diagram of the transfer of Cy5e-sgc8 from Cy5-sgc8-eNC-I to Chol-FAM-NC-I, generating M-NC-2 consisting of Cy5-sgc8-eNC-II and Chol-FAM-NC-II. For Cy5-sgc8-eNC-I, Cy5 fluorophore was attached to e-sgc8 (called Cy5e-sgc8), while Chol-FAM-NC-I contained Chol-e-Tc and Tb'-FAM. After binding to Cy5e-sgc8, Chol-FAM-NC-I (essentially forming Chol-FAM-NC-II) can emit two types of fluorescence signals. (b) CLSM images of Si-DLBM exposed to M-NC-2. (c) CLSM images of Si-DLBM exposed to only Cy5-sgc8-eNC. (d) CLSM images of Si-DLBM exposed to only Chol-FAM-NC-I. The experiments were performed as described in the section of “Dual-color fluorescence imaging of Si-DLBM”. Scale bar = 2.5 μm.

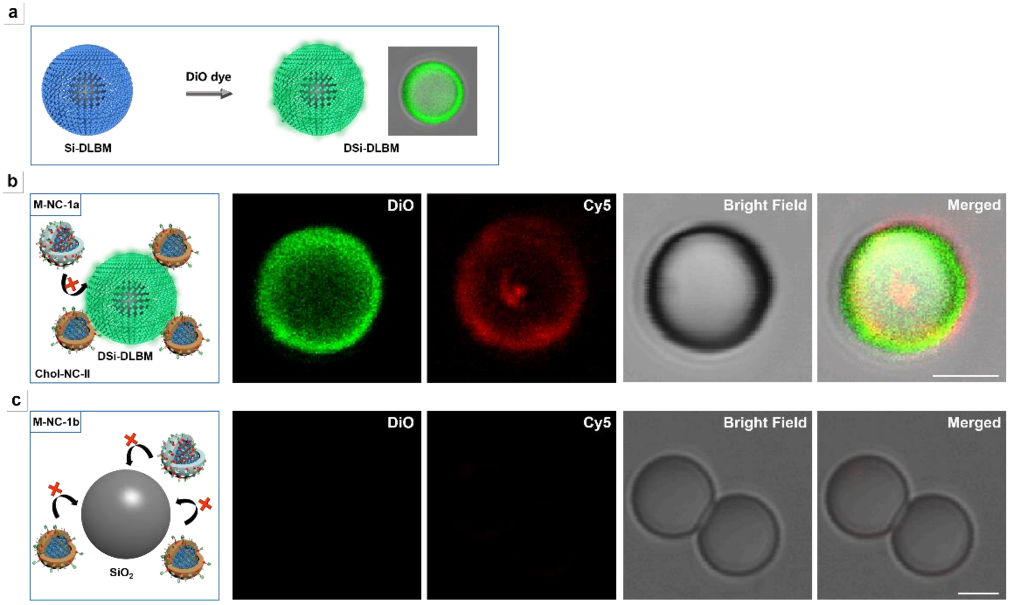

Figure S29. Validation of the hydrophobic interactions between Chol-NC and Si-DLBM particles with the help of dye-stained Si-DLBM. (a) Schematic diagram of staining the DOPC liposome bilayer of Si-DLBM with 3,3-dioctadecyloxacarbocyanine perchlorate cell membrane dye (abbreviated as DiO). The resulting DiO stained Si-DLBM was abbreviated as DSi-DLBM. (b) CLSM image of DSi-DLBM incubated with M-NC-1 consisting of Cy5-sgc8-eNC-II and Chol-NC-II capable of fluorescing. (c) The same as (b) but bare silica microsphere (SiO_2) was used instead of DSi-DLBM. Scale bar = 2.5 µm.

Experimental procedure:

The DiO cell membrane dye (Beyotime, Jiangsu, China) was used to stain Si-DLBM. Briefly, 50 µL of Si-DLBM was subjected to ultrasonic treatment (40 KHz, 5min), and then 2.5 µL of DiO storage solution (100 µM, in ethanol) was added and mixed thoroughly. The final concentration of DiO was 5 µM. The resulting solution was incubated at room temperature for 10 min, and the excess dye DiO was removed by centrifugation at 10000 RPM for 10 min. The precipitates were washed three times with ultra-pure water and resuspended in 50 µL of 1 × T4 buffer, obtaining DSi-DLBM.

The samples for the fluorescence imaging were prepared as described in Figure S27, but DSi-DLBM and bare SiO_2 microsphere were substituted for Si-DLBM in panel b and panel c, respectively. The CLSM imaging was performed with a 20× water objective under imaging conditions of DiO (λ_{ex} = 488 nm, λ_{em} = 518 nm) and Cy5 (λ_{ex} = 645 nm, λ_{em} = 670 nm) on Leica TCS SP8 inverted confocal microscope (Leica, Germany).

Discussion:

DiO dye is a lipophilic membrane dye that can dye the phospholipid bilayer on the surface of Si-DLBM and emit green fluorescence under the excitation wavelength of 488 nm (λ_{ex} = 488 nm, λ_{em} = 518 nm) (Figure S29a). As show in panel DiO of Figure S29b, the green fluorescence of DiO appears when the excitation wavelength of 488 nm was adopted to excite the DSi-DLBM, while no green florescence is observed in Figure S29c, indicating the formation of DOPC liposome bilayer on DSi-DLBM. Similarly, when the excitation wavelength of 645 nm was used to excite the DSi-DLBM treated with M-NC-1 consisting of Cy5-sgc8-eNC-II and Chol-NC-II, the red fluorescence of Cy5 is detected in panel Cy5 of Figure S29b. Under identical conditions, there is no red fluorescence in Figure S29c. These fluorescence images demonstrate that, to capture Chol-NC onto SiO_2 microsphere, it is necessary to prepare Si-DLBM by coating SiO_2 microsphere with DOPC liposome bilayer. Moreover, Cy5 fluorescence signal does originate from the transfer of Cy5-modified aptamer onto Chol-NC-I (forming Chol-NC-II) rather than from the non-specific adsorption onto SiO_2 microsphere.

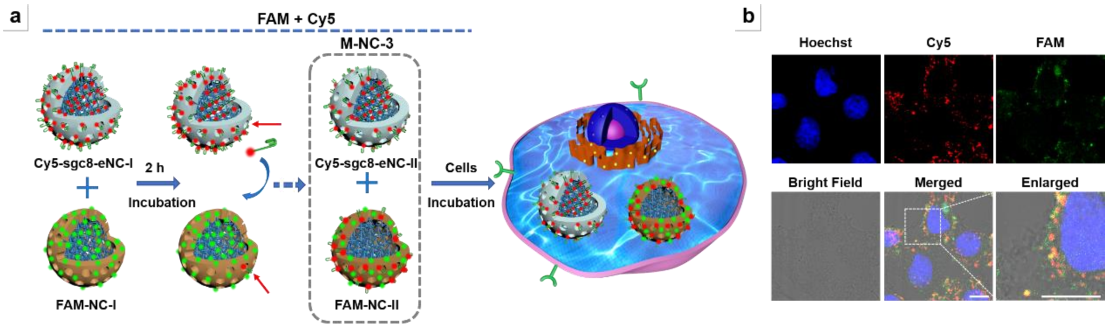

Figure S30. Validation of aptamer transfer between different DNA NCs at the cellular level by dual-color fluorescence imaging. (a) Schematic diagram of the incubation of Cy5-sgc8-eNC-I first with FAM-NC-I and then with HeLa cells, leading to the transfer of Cy5e-sgc8 from the former to the latter and the cellular internalization. After receiving Cy5e-sgc8, FAM-NC-II has two fluorophores: Cy5 and FAM. (b) Dual-color CLSM images of the resulting cells. Scale bar = 20 μm.

Experimental procedure:

The FAM-NC-I and Cy5-sgc8-eNC-I were assembled through the previously described procedure (seen in "Stepwise assembly of DNA nanocluster"), but Tb'-FAM and e-Tc were instead used for the former assembly and Cy5e-sgc8 was substituted for e-sgc8 for the latter assembly. Then, FAM-NC-I (25 μL) was mixed Cy5-sgc8-eNC-I (25 μL) and incubated at room temperature for 2 h. After mixing with 150 μL of culture medium, the resulting solution was incubated with HeLa cells in a humidified atmosphere of 5% CO_2 at 37°C for 2 h. Afterward, the culture medium was removed, and the cells were washed twice with PBS and fixed with 4% paraformaldehyde for 15 min, followed by staining with Hoechst 33342 (10 μg/mL) for 5 min. Subsequently, the Hoechst staining solution was removed, and the cells were washed twice with PBS. Finally, 2 μL of Antifade Mounting Medium was added dropwise onto the center of cover slip and placed upside down on a microscope slide. The fluorescence imaging of cells was performed on a Leica SP8 laser scanning confocal microscope (CLSM, Leica, Germany). Hoechst 33342 was excited at a wavelength of 405 nm, while FAM was excited at 488 nm and Cy5 was excited at 638 nm.

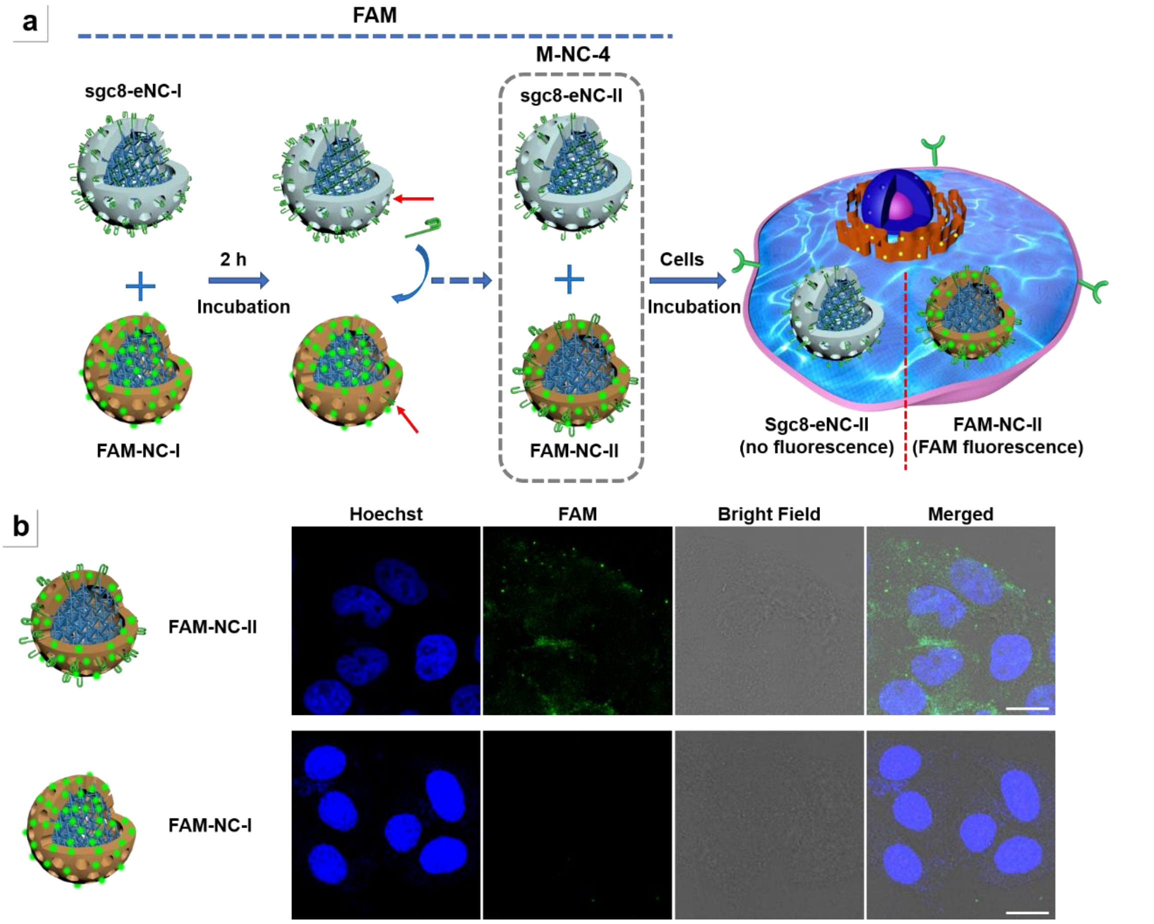

Figure S31. Validation of aptamers transfer between different DNA NCs by label-free aptamer-mediated cellular internalization. (a) Schematic diagram of the incubation of label-free sgc8-eNC-I first with FAM-NC-I and then with HeLa cells. The internalization of FAM-NC-II into cells requires the transfer of aptamers. Only by receiving the e-sgc8 transferring from sgc8-eNC can FAM-NC (essentially forming FAM-NC-II) enter the cells and fluoresce upon the laser excitation when fluorescence imaging. (b) Single-color CLSM images of cells incubated with the mixture (called M-NC-4) of sgc8-eNC-II/FAM-NC-II (upper panel) or with only FAM-NC-I (lower panel). Scale bar = 20 μm.

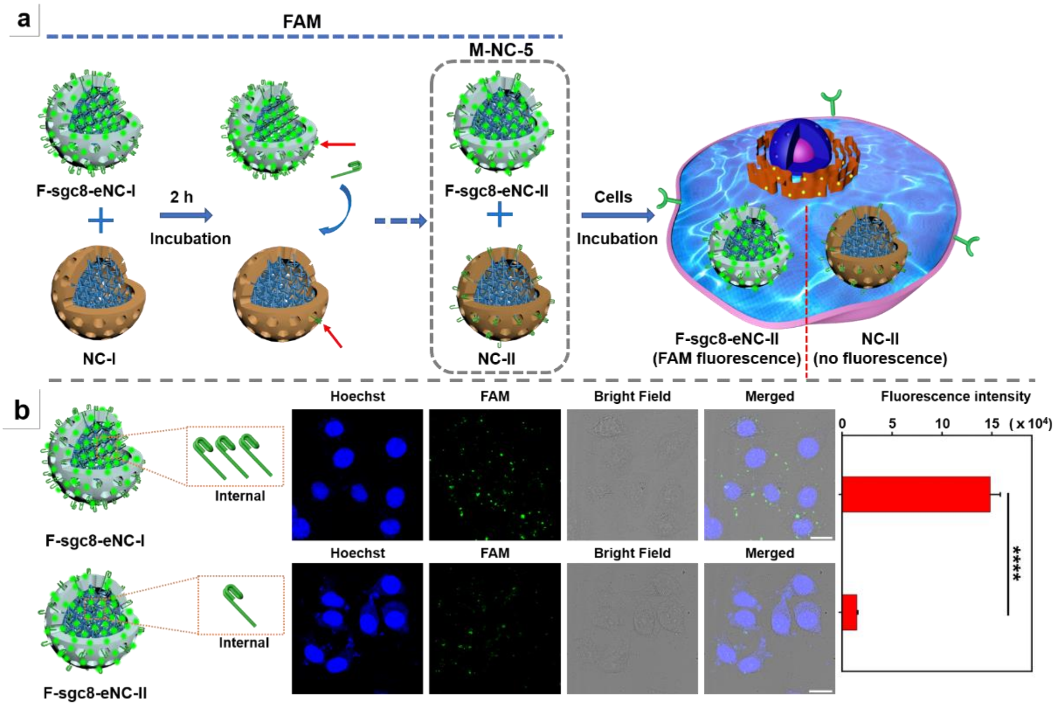

Figure S32. Validation of aptamer transfer out from F-sgc8-eNC by measuring the decrease in the fluorescence intensity of internalized nanostructures *via* CLSM technique. (a) Schematic diagram of the incubation of F-sgc8-eNC-I first with label-free DNA basic NC and then with HeLa cells, leading to the inter-particle transfer of e-sgc8 and cellular internalization. For F-sgc8-eNC-I, only Tb'-FAM component can fluoresce. Meanwhile, because label-free e-sgc8 was used in this section, even if it transfers from F-sgc8-eNC-I to DNA NC-I (forming F-sgc8-eNC-II and NC-II), the internalized NC-II is invisible by confocal microscopy. (b) Single-color CLSM images of the cells incubated with only F-sgc8-eNC-I or with the mixture (M-NC-5) of F-sgc8-eNC-II and invisible NC-II. In the right: the quantitative fluorescence intensity per cell calculated via dividing the total fluorescence intensity of the whole image by cell number. ****$P < 0.0001$, two-tailed unpaired t test. Scale bar = 20 μm.

Discussion for Figures S30-S32:

The transfer of aptamer between different NCs was validated *via* performing CLSM imaging of cells. When Cy5-sgc8-eNC and FAM-NC were pre-incubated at room temperature and then incubated with HeLa cells, two different fluorescence signals are expected to appear because of the aptamer transfer (Figure S30a). As could be expected for aptamer redistribution to FAM-NC, besides representative Cy5 fluorescence signal from target cells, FAM fluorescence appears as shown in Figure S30b, proving the internalization of both FAM-NC and Cy5-sgc8-eNC. If sgc8-eNC is not modified with the fluorophores (label-free e-sgc8 used instead) (Figure S31a), the cells incubated with the mixture of FAM-NC-I and sgc8-eNC-I still display green FAM fluorescence (the upper of Figure S31b), while incubation with FAM-NC alone does not produce any fluorescence signal due to the lack of aptamers (the lower of Figure S31b), intuitively indicating that e-sgc8 does transfer to FAM-NC.

Because only F-sgc8-eNC (including type I and type II) fluoresces (NC-I and NC-II have no fluorophores, Figure S32a), the fluorescence intensity from the cells is considered to manifest the cellular internalization ability of F-sgc8-eNC. As shown in CLSM images of Figure S32b, the cells treated with F-sgc8-eNC-I show a strong fluorescence signal (upper panel). In comparison, after incubating F-sgc8-eNC-I with DNA NC-I in advance, the corresponding cells show a weak fluorescence signal (lower panel), which is proved by the quantitative fluorescence signal in the bar graphs (right part), indicating the decrease of the internalization ability upon co-incubation with DNA NC-I. Namely, the number of aptamers in F-sgc8-eNC-II is smaller than F-sgc8-eNC-I, demonstrating the transfer of aptamers.

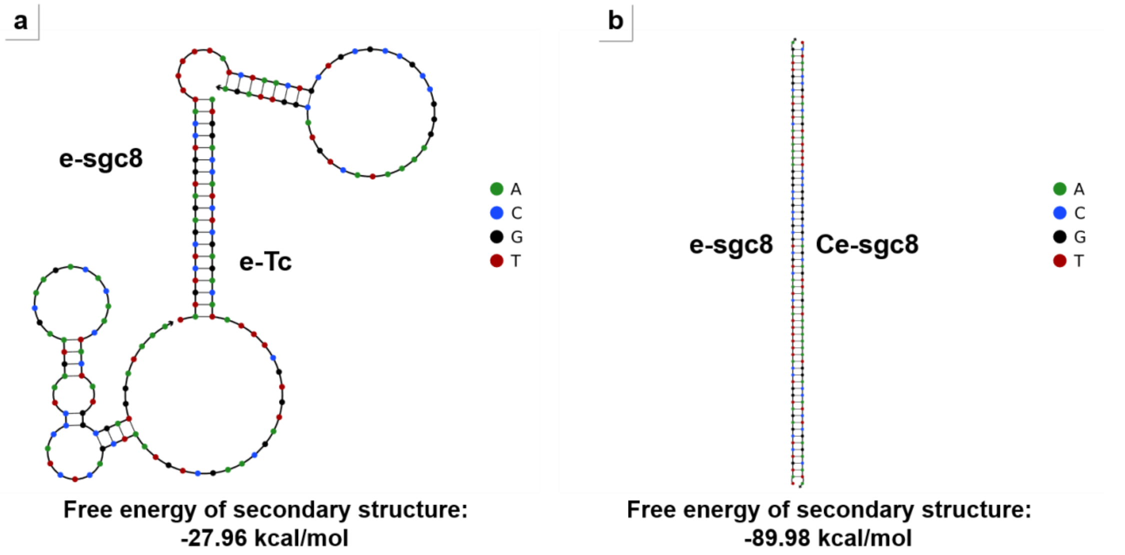

Figure S33. Free energy change for the secondary structure of aptamer-incorporated DNA duplex, which was calculated by the website (http://www.nupack.org/). Panel a: the part hybridization between e-Tc and e-sgc8; Panel b: the complete hybridization between e-sgc8 and Ce-sgc8. The lower free energy for Ce-sgc8/e-sgc8 duplex indicates that Ce-sgc8 can compete for e-sgc8 with the e-Tc strand and thus was used to peel off e-sgc8 from sgc8-eNC in the subsequent experiments.

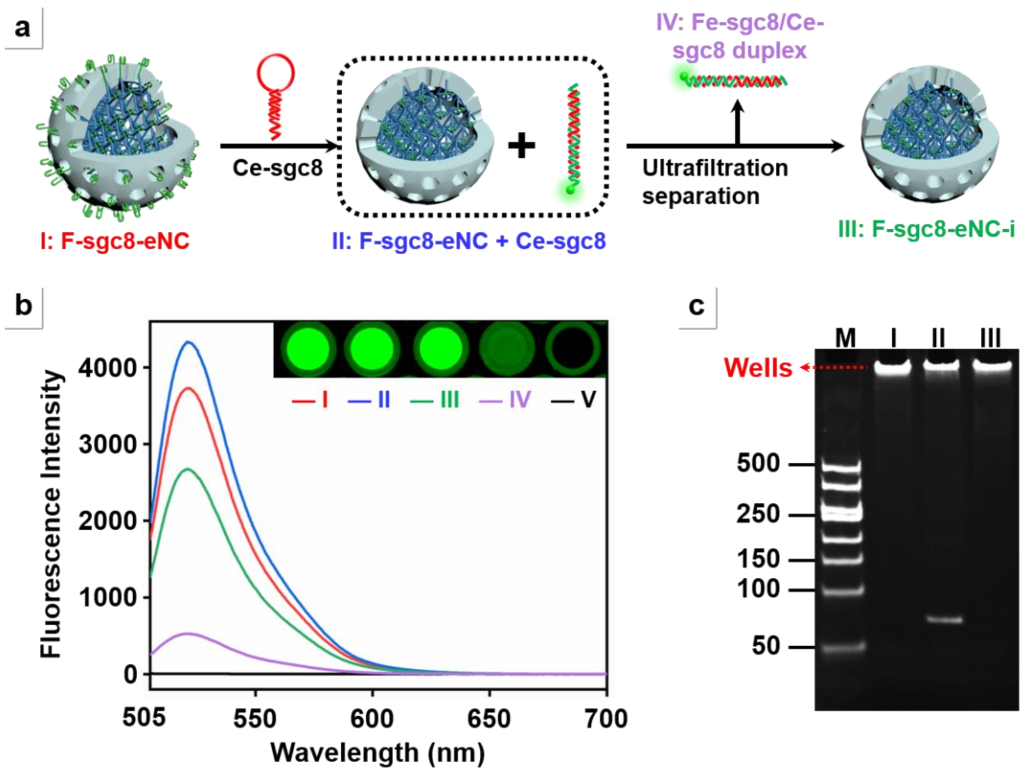

Figure S34. Peeling off the surface-hybridized aptamers from sgc8-eNC by competitive hybridization to its complementary probe. (a) Schematic diagram of hybridization competition for Fe-sgc8 between FAM-sgc8-eNC and Ce-sgc8, accompanied by ultrafiltration separation. The e-Tc component in FAM-sgc8-eNC is partly complementary to Fe-sgc8, while Ce-sgc8 completely hybridizes with Fe-sgc8. (b) The fluorescence spectra of FAM-sgc8-eNC before and after peeling off the surface-confined Fe-sgc8 by hybridization competition: I, FAM-sgc8-eNC; II, the mixture of FAM-sgc8-eNC + Ce-sgc8; III, the same as II but subjected to ultrafiltration separation (FAM-sgc8-eNC-i); IV, Fe-sgc8/Ce-sgc8 duplex in the filtrate; V, Blank (1 × T4 DNA Ligase buffer). (C) Gel electrophoresis to verify the feasibility of ultrafiltration centrifugation for the separation of Fe-sgc8/Ce-sgc8 duplex from FAM-sgc8-eNC-i. M stands for DNA marker. Samples I, II and III are the same as panel b. DNA Marker was stained with SYBR Green I, while samples I, II and III was imaged via exciting FAM fluorescence. The red arrow is the position of gel/well interface.

Experimental procedure:

The FAM-sgc8-eNC was assembled as described in the section of "Stepwise assembly of DNA nanocluster", but the Fe-sgc8 was used instead of e-sgc8. Then, Ce-sgc8 (1 μL, 10 μM) was added to FAM-sgc8-eNC (100 μL) (Sample I), allowed to keep at room temperature for 30 min (formatting Sample II). Afterward, 50 kD MerckMillipore Amicon® Ultra was used to separate the filtrate from the particle pellets. Specifically, 100 μL of 1 × T4 DNA Ligase buffer was used to equilibrate the inner membrane of ultrafiltration tube, and then centrifuged at 4000 RPM for 5 min. Sample II (100 μL) was added to the ultrafiltration tube and centrifuged at 10000 RPM for 5 min. After transferring the filtrate to a centrifuge tube, the ultrafiltration tube was washed twice with 1 × T4 DNA Ligase buffer (50 μL). The washing solutions were collected and merged into the filtrate, obtaining Sample IV. Moreover, 50 μL of 1 × T4 DNA Ligase buffer was added into the ultrafiltration tube and carefully washed filter membrane with a pipette. The inner tube of ultrafiltration tube was placed upside down, followed by centrifugation at 10000 RPM for 5min. The dissolution, washing and centrifugation process was repeated once, and the resulting solutions were collected and merged together, obtaining Sample III.

Before the fluorescence measurement, all the samples were diluted to 200 μL with 1 × T4 DNA Ligase buffer. The fluorescence spectra were collected on a fluorescence spectrometer (Hitachi, Ltd., Japan) and the instrument parameters were described as follows: λ_{ex} = 495.0 nm; λ_{em} = 505.0 - 700.0 nm; Scan speed: 240 nm/min; PMT Voltage: 700 V; Response: 2.0 s). The samples I-IV were also transferred into the

cuvettes for fluorescence photographing by Amersham Typhoon scanners (GE Healthcare Bio-Sciences AB, Sweden). Equal volume of 1 × T4 DNA Ligase buffer was used as Blank.

For gel electrophoresis analysis, each of DNA samples (10 µL) was mixed with 2 µL of 6 × Loading buffer, while the DNA marker (5 µL) was mixed with 2 µL of 10 × SYBR Green I and 2 µL of 6 × Loading buffer. The resulting solutions were allowed to keep at the room temperature for 5 min and then loaded separately into the wells of electrophoretic gel. The nPAGE (6%) was run at 80 V for 60 min in a 0.5 × TBE buff er on a gel electrophoresis instrument (Bio-RAD, USA). The gel image was photographed on a ChemiDoc™ XRS Imaging system with the image acquisition and analysis software Image Lab (Bio-RAD, USA).

Discussion:

As illustrated in Figure 34a, the surface-hybridized aptamers are peeled off from sgc8-eNC by the preferential hybridization to the complementary probes and then extracted by ultrafiltration centrifugation for fluorescence measurement. As shown in Figure S34b, FAM-sgc8-eNC displays a high fluorescence peak (Line I). The difference in fluorescence intensity between Sample I and Sample II indicates the hybridization reaction between FAM-sgc8-eNC and Ce-sgc8. Moreover, falling out of Fe-sgc8 from FAM-sgc8-eNC could cause the increase of spatial distance between fluorophores and result in the restoration of self-quenched FAM emission, thereby leading to the slightly stronger fluorescence intensity than FAM-sgc8-eNC (Sample I). The fluorescence intensity of Sample III is weaker than Samples I and II, demonstrating that some Fe-sgc8 probes were peeled off from FAM-sgc8-eNC and extracted after ultrafiltration centrifugation, which is further validated by the obvious fluorescence signal detected from the filtrate (sample IV). The fluorescence photographs in the Inset show the identical trend of fluorescence change. The more reliable evidence is the change in electrophoretic behavior of FAM-sgc8-eNC. As shown in Figure S34c, only single one DNA band is observed for Sample I. There is an obvious band with the faster mobility besides a main band in Sample II, indicating the formation of Fe-sgc8/Ce-sgc8 duplex. Only one main band is detected in Sample III, suggesting that Fe-sgc8/Ce-sgc8 duplex were removed by ultrafiltration centrifugation.

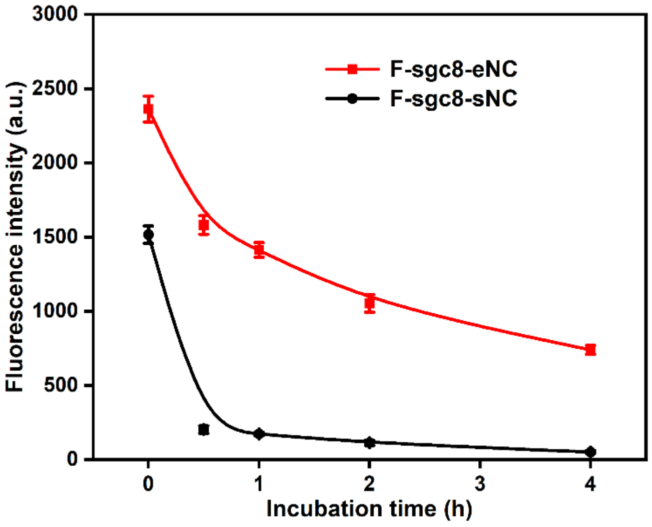

Figure S35. Time-dependent hybridization competition of Ce-sgc8 for e-sgc8 with DNA NC. The change in the fluorescence intensity of F-sgc8-eNC (red line) and F-sgc8-sNC (blacke line) in the presence of excess Ce-sgc8 was recorded at different incubation time points after ultrafiltration. The error bars represent the standard deviation (n = 3).

Experimental procedure:

FAM-sgc8-eNC was assembled as described in the section of "Stepwise assembly of DNA nanocluster", but the Fe-sgc8 was used instead of e-sgc8. Then, excess Ce-sgc8 (4 µL, 10 µM) was added to FAM-sgc8-eNC (100 µL), followed by keeping at room temperature for different time periods (0, 0.5, 1, 2 or 4 h). Afterward, 50 kD MerckMillipore Amicon® Ultra was used to separate the filtrate from the particle pellets. Specifically, 100 µL of 1 × T4 DNA Ligase buffer was used to equilibrate the inner membrane of ultrafiltration tube, and then centrifuged at 4000 RPM for 5 min. The reaction solution (104 µL) was added to the ultrafiltration tube and centrifuged at 10000 RPM for 5 min. After the filtrate was discarded, 200 µL of 1 × T4 DNA Ligase buffer was added into the ultrafiltration tube and carefully washed filter membrane with a pipette, followed by centrifugation at 10000 RPM for 5 min. The filtrate was discarded, and this operation was repeated twice. Next, 100 µL of 1 × T4 DNA Ligase buffer was added into the ultrafiltration tube and carefully washed filter membrane with a pipette. The inner tube of ultrafiltration tube was placed upside down and centrifugated at 10000 RPM for 5 min. This operation was repeated once, and the volume of resulting solution was 200 µL. The fluorescence spectra were collected on a fluorescence spectrometer (Hitachi, Ltd., Japan) and the instrument parameters were described as follows: λ_{ex} = 495.0 nm; λ_{em} = 505.0 - 700.0 nm; Scan speed: 240 nm/min; PMT Voltage: 700 V; Response: 2.0 s). The experimental procedure for exploration of the hybridization between Ce-sgc8 and FAM-sgc8-sNC is the same as FAM-sgc8-eNC, but FAM-sgc8-sNC was instead used.

Discussion:

In order to intuitively verify the outward migration of aptamers from the internal cavity, we incubated F-sgc8-eNC with excess Ce-sgc8 for different time periods and monitored the remaining fluorescence emitting from DNA nanoclusters after ultrafiltration. As shown in Figure S35, the initial fluorescence intensity of F-sgc8-eNC (red line) is obviously higher than F-sgc8-sNC (black line), indicating that more Fe-sgc8 can be loaded during the assembly of F-sgc8-eNC. It is reasonable because, compared with F-sgc8-sNC, F-sgc8-eNC accommodate Fe-sgc8 in the internal cavity besides the furface. Moreover, the fluorescence intensity of F-sgc8-sNC rapidly decreases in the initial stage and is almost exhausted (only 13.4% remained) within 0.5 h, indicating a rapid surface hybridization process between Fe-sgc8 and Ce-sgc8. In contrast, most (66.9%) of the fluorescene intensity of F-sgc8-eNC is retained after 0.5-h incubation and then only slowly decreases over the entire period of subseqent incubation. Even after 4-h incubation,

the residual fluorescence intensity still is more than 30%, indicating a gradual hybridization process between Fe-sgc8 and Ce-sgc8. It is natural because Fe-sgc8 needs more time to move outward from the internal cavity of F-sgc8-eNC and then reach the surface and hybridize with Ce-sgc8. These experimental results demonstrate that, besides anchoring onto the surface of DNA eNC, targeting aptamers are encapsulated in the internal cavity and can gradually move outward as neended, thereby ensuring that sgc8-eNC maintains its high cell-targeting ability during the drug delivery process.

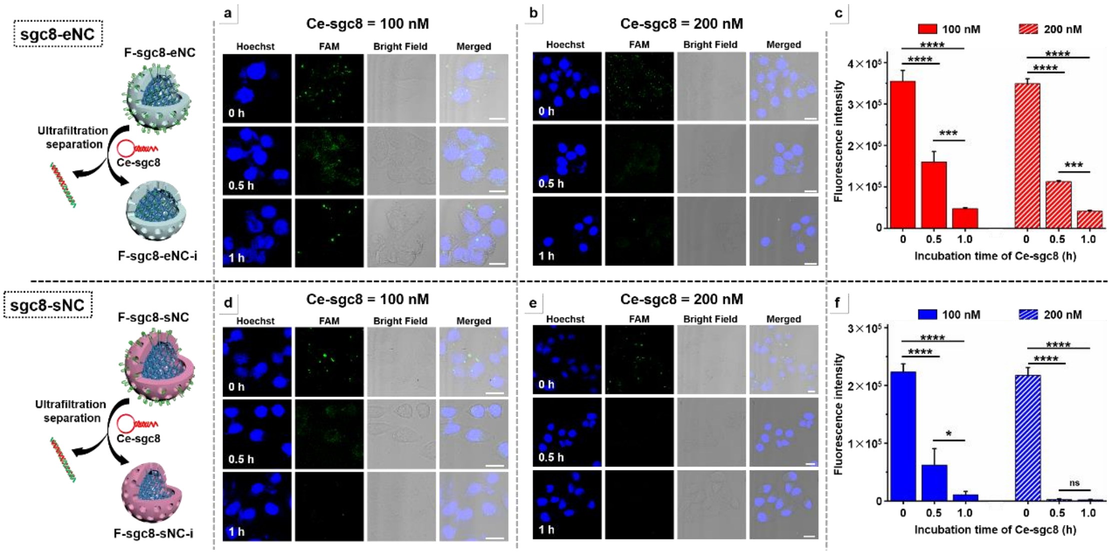

Figure S36. The influence of incubation time and Ce-sgc8 concentration on the hybridization competition with DNA NC for e-sgc8. In the far left is the schematic illustration of hybridization competition of Ce-sgc8 with F-sgc8-eNC (upper) or F-sgc8-sNC (lower) for Fe-sgc8. After Fe-sgc8/Ce-sgc8 duplex is separated by ultrafiltration centrifugation, the corresponding products are F-sgc8-eNC-i and F-sgc8-sNC-i, respectively. (a) CLSM image of HeLa cells subjected to F-sgc8-eNC-i that was pre-treated with 100 nM Ce-sgc8 by incubating for different time periods. (b) The same as (a) but 200 nM Ce-sgc8 was involved. (c) The quantitative fluorescence intensity estimated from CLSM images in panels b and c by Image J software. (d), (e) and (f) are the same as panels a, b and c, respectively, but F-sgc8-sNC was substituted for F-sgc8-eNC. The statistical analysis of multiple comparisons were evaluated by one-way ANOVA. *P < 0.05, **P < 0.005, ***P < 0.0005, ****P < 0.0001, and ns represents no significant difference. Scale bar = 20 μm.

Experimental procedure:

A given amount of Ce-sgc8 was mixed with F-sgc8-eNC (100 μL, 200 nM). The final concentration of the former was 100 nM or 200 nM, while the final concentration of the latter was 100 nM. The reaction solution was allowed to keep at room temperature for a given time point (0, 0.5 or 1 h). Afterward, 50 kD MerckMillipore Amicon® Ultra was used to separate the filtrate from the particle pellets. Specifically, 100 μL of 1 × T4 DNA Ligase buffer was used to equilibrate the inner membrane of ultrafiltration tube, and then centrifuged at 4000 RPM for 5 min. The reaction solution (100 μL) was added to the ultrafiltration tube and centrifuged at 10000 RPM for 5 min. The filtrate was discarded and the ultrafiltration tube was washed twice with 1 × T4 DNA Ligase buffer (50 μL). Subsequently, 50 μL of 1 × T4 DNA Ligase buffer was added into the ultrafiltration tube and carefully washed filter membrane with a pipette. The inner tube of ultrafiltration tube was placed upside down, followed by centrifugation at 10000 RPM for 5min. The dissolution, washing and centrifugation process was repeated once, and the pellets were resuspended in 100 μL of 1 × T4 DNA Ligase buffer, obtaining F-sgc8-eNC-i solution.

For the cell fluorescence imaging, the F-sgc8-eNC-i (25 μL) was mixed with 175 μL of culture medium and incubated with HeLa cells (5×10^4 cells) in a humidified atmosphere of 5% CO_2 at 37 °C for 2 h. Afterward, the culture medium was removed, and the cells were washed twice with PBS and fixed with 4% paraformaldehyde for 15 min, followed by staining with Hoechst 33342 (10 μg/mL) for 5 min. Subsequently, the Hoechst staining solution was removed, and the cells were washed twice with PBS. Finally, 2 μL of Antifade Mounting Medium was added dropwise onto the center of cover slip and placed upside down on a microscope slide. The fluorescence imaging of cells was performed on a Leica SP8 laser scanning confocal microscope (CLSM, Leica, Germany). Hoechst 33342 was excited at a wavelength of 405 nm, while FAM was excited at 488 nm. Similar experimental procedure was adopted for the preparation of F-sgc8-sNC-i solution and for subsequent cell treatment before fluorescence imaging.

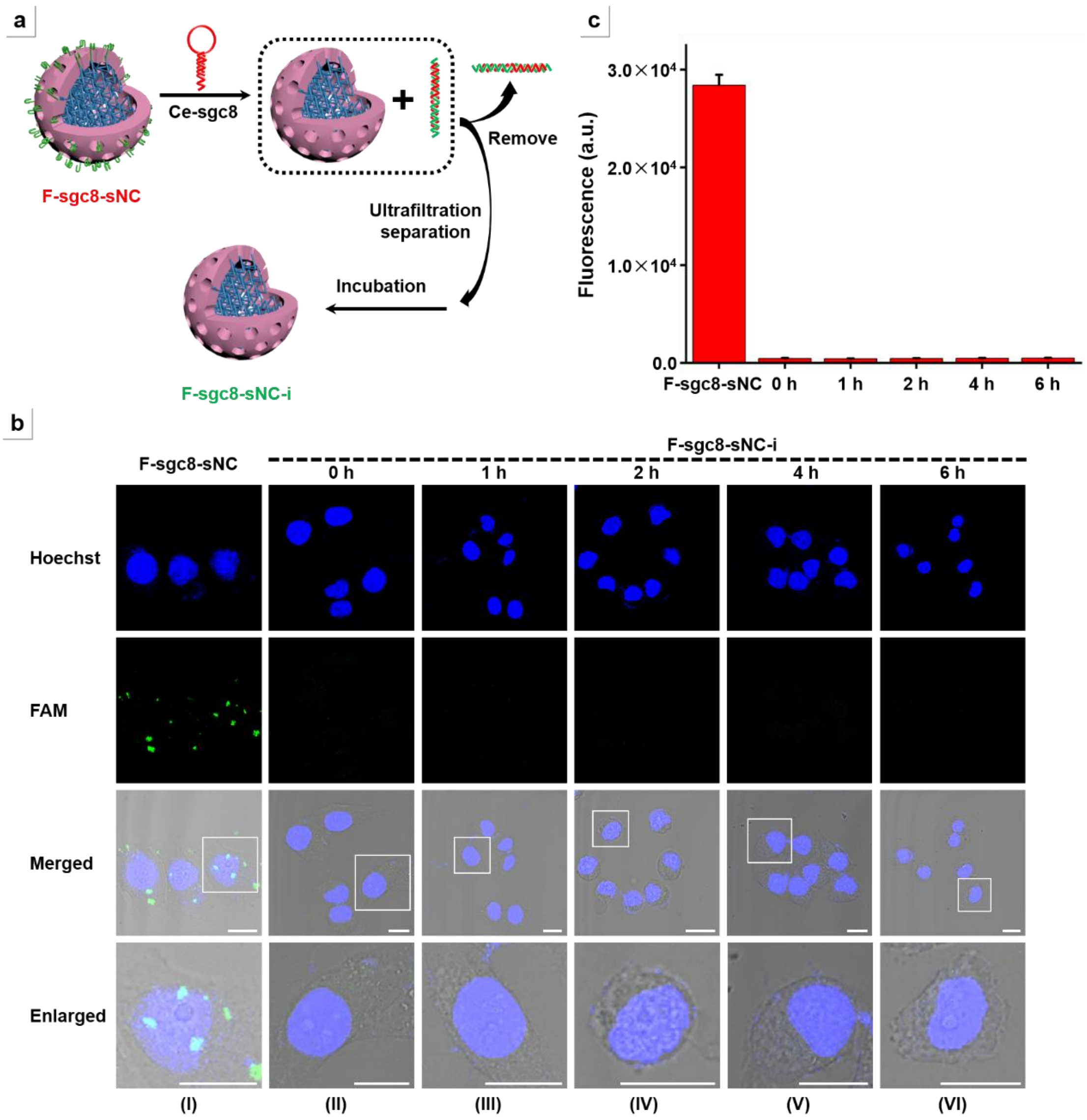

Figure S37. Possible mechanism for peeling off surface-hybridized aptamers from sgc8-sNC. (a) Schematic diagram of the displacement of surface-hybridized e-sgc8s by Ce-sgc8 and subsequent ultrafiltration centrifugation and incubation for a given time interval. The corresponding product is called F-sgc8-sNC-i. (b) CLSM images of HeLa cells treated with F-sgc8-sNC or F-sgc8-eNC-i that was prepared with the following three steps: incubating F-sgc8-sNC with Ce-sgc8 for 30 min, ultrafiltration centrifugation and allowing F-sgc8-sNC-i solution to stand for 1 h, 2 h, 4 h or 6 h. Scale bar = 20 µm. (c) Quantitative fluorescence intensity recorded from the same cells as panel b. The experiments were performed as described in the section of "Outward movement of aptamers in the cavity of sgc8-eNC toward the surface" but sgc8-sNC was used instead of sgc8-eNC.

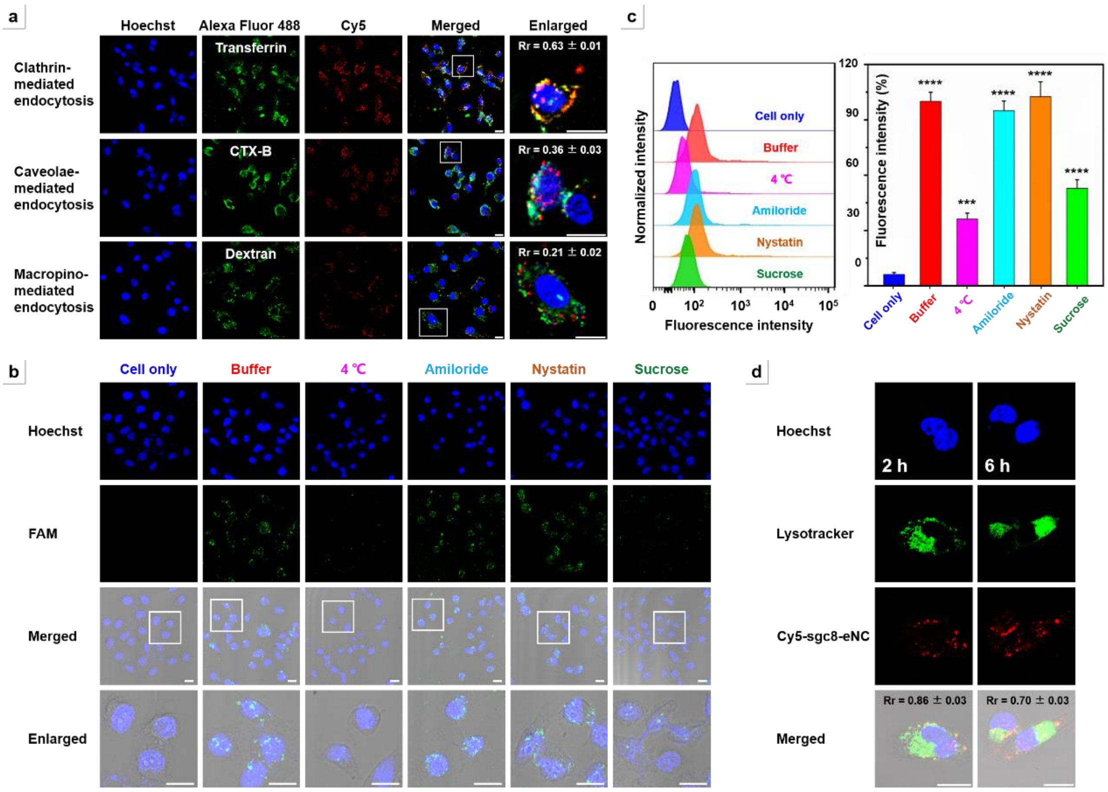

Figure S38. The endocytosis pathway of sgc8-eNC and endosomal escape properties. (a) Colocalization analysis of the fluorescent markers (green fluorescence) of different endocytosis pathway with Cy5-sgc8-eNC (red fluorescence) by CLSM imaging of HeLa cells. (b) CLSM analysis of HeLa cells treated with FAM-sgc8-eNC in the presence of different endocytosis inhibitors or at 4°C, accompanied by the corresponding flow cytometry analysis (c). Buffer indicates the absence of any inhibitor. The right part is the corresponding quantitative fluorescence intensity recorded from flow cytometry data. Error bars represent the standard deviation (n = 3). The statistical analysis of multiple comparisons were evaluated by one-way ANOVA. ***P < 0.0005, ****P < 0.0001. (d) CLSM images of Cy5-sgc8-eNC (red fluorescence) colocalizing (yellow fluorescence) with LysoTracker Green (green signal) at different time intervals of uptake. Rr represents the Pearson correlation coefficient that was obtained *via* Image J software. Scale bar = 20 μm.

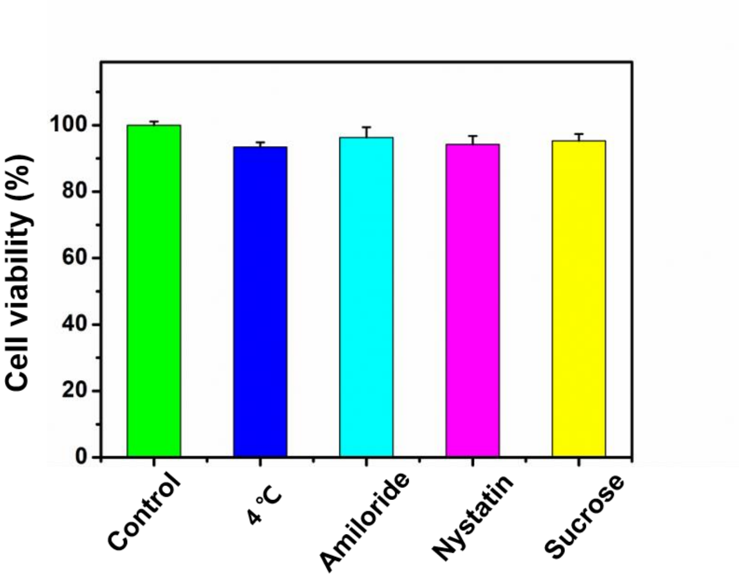

Figure S39. General totoxicity of the inhibitors of endocytic pathways toward HeLa cells. The HeLa cells were separately incubated with amiloride (133 µg/mL), nystatin (15 µg/mL) and sucrose (154 mg/mL) or at 4℃ for 2 h. The cell viability was evaluated via CCK-8 assay. The error bars represent the standard deviation (n = 3).

Experimental procedure:

HeLa cells (2000) were cultured in a 96-well plastic plate with complete culture medium (10% FBS and 1% penicillin-streptomycin) for overnight at 37°C in a humidified atmosphere of 5% CO_2. After washing three times with PBS buffer, to evaluate the general toxicity of inhibitors, 10 µL of inhibitor and 90 µL of culture medium were added, and the cells were further cultured in a humidified atmosphere of 5% CO_2 at 37°C for 2 h. For low temperature treatment, the HeLa cells were cultured in 100 µL of culture medium at 4°C for 2 h with protection from light. The resulting cells were washed three times with PBS buffer and further cultured in 100 µL of complete culture medium (with 10% FBS) in a humidified atmosphere of 5% CO_2 at 37°C for 24 h. Subsequently, 10 µL of CCK-8 solution (Meilun, China) was added to each well and incubated for 2 h at 37°C in a humidified atmosphere. The absorbance of 450 nm of different wells was measured using a NanoQuant Absorbance Microplate Reader (Tecan Infinite M200 PRO, Austria).

Discussion:

Cytotoxicity of the inhibitors of endocytic pathways or low temperature treatment toward HeLa cells for a given time period was evaluated via CCK-8 assay. One can notice that the cell viability of HeLa cells is higher than 90% regardless of the nature of inhibitors, and there is also no obvious cell damage upon incubation at 4℃, indicating that these inhibitors and low temperature treatment have no obvious cytotoxicity toward HeLa cells.

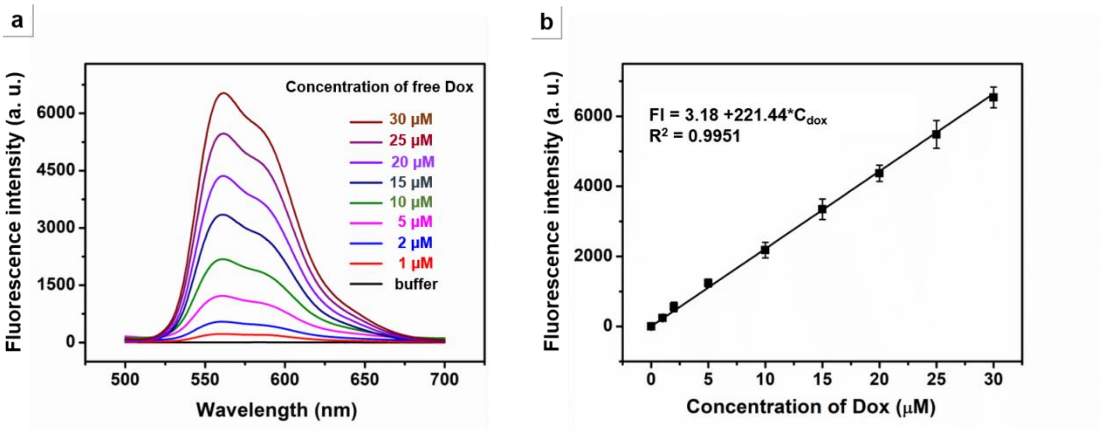

Figure S40. The standard linear calibration curve of Doxorubicin (Dox) fluorescence versus its concentration. (a) The fluorescence spectra of free Dox at different concentrations. (b) The linear relationship between Dox concentration and fluorescence intensity (560 nm), accompanied by a standard linear regression equation in Inset. The error bars represent the standard deviation (n = 3). The experimental procedure is shown in the section of "Drug Loading".

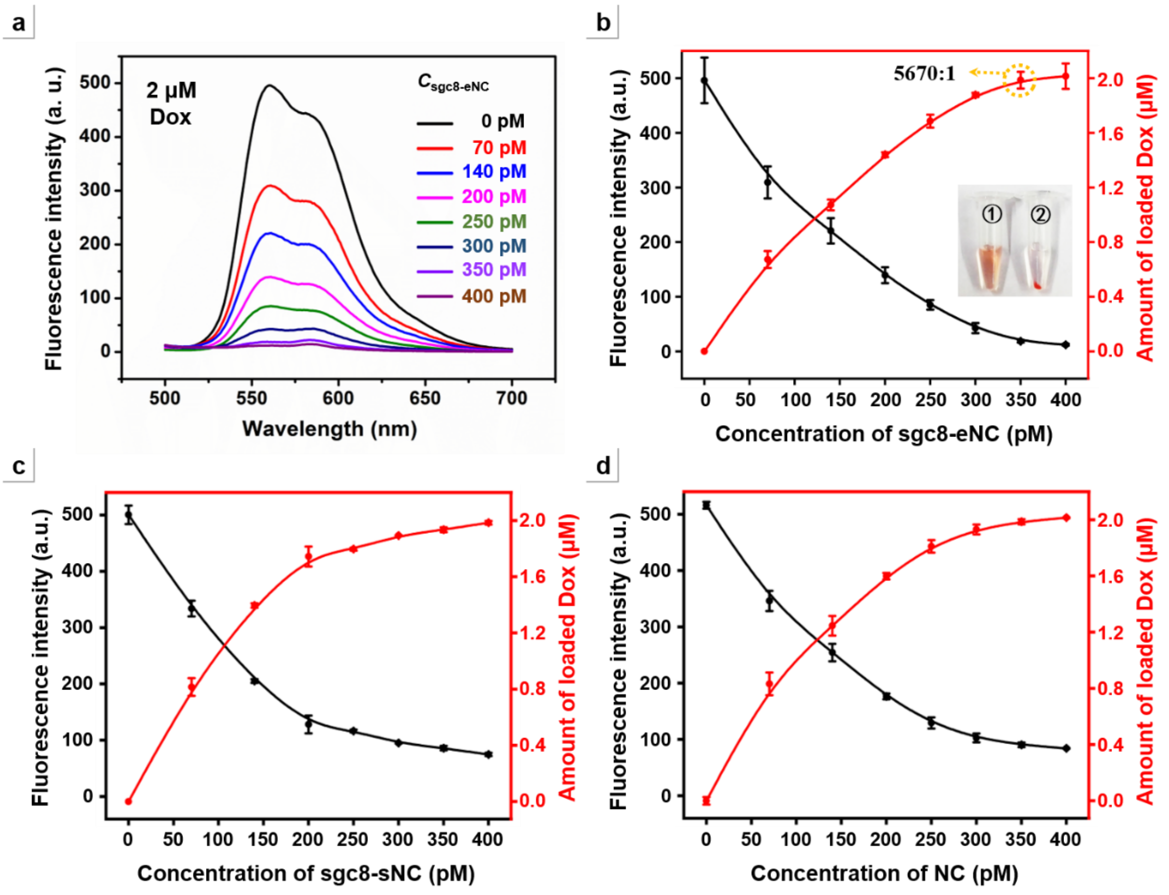

Figure S41. Drug payload capacity of different DNA assemblies. (a) Fluorescence spectrum of Dox (2 μM) in the presence of different concentrations of sgc8-eNC. The fluorescence quenching occurs when Dox is intercalated into the ds-DNA fragments in sgc8-eNC. (b) Corresponding fluorescence peak intensity of Dox at 560 nm (black) and concentration of Dox loaded (red). Inset: Photographs of Dox in the absence ① and presence of ② sgc8-eNC at molar ratio of 5670:1. (c) Fluorescence peak intensity of Dox (2 μM) in the presence of different concentrations of sgc8-sNC (black line) and corresponding concentration of Dox loaded (red line). (d) Fluorescence peak intensity of Dox (2 μM) in the presence of different concentrations of NC (black line) and corresponding concentration of Dox loaded (red line). The error bars represent the standard deviation (n = 3). The experimental procedure is shown in the section of "Drug Loading".

Discussion for Figures S40 and S41:

Firstly, the standard linear calibration curve was constructed by measuring the fluorescence intensity in the presence of given concentration of Doxorubicin (Dox) fluorescence. As shown in Figure S40, one can observed a good linear relationship between the fluorescence intensity of Dox and its concentration. Then, the concentration of sgc8-eNC was estimated from the following formula:

$$C_{sgc8\text{-}eNC} = C_{sgc8\text{-}Tetra}/(V_{sgc8\text{-}eNC}/V_{sgc8\text{-}Tetra})$$

$C_{sgc8\text{-}Tetra}$ is equal to the concentration of DNA component since the assembly proceeds at a molar ratio of 1:1, while the value of $V_{sgc8\text{-}eNC}/V_{sgc8\text{-}Tetra}$ is estimated to be 283 as discussed in Scheme S4. Meanwhile, Figure S41a reflects the loading capability of sgc8-eNC that is determined by the dependence of Dox fluorescence on the concentration of sgc8-eNC. When Dox concentration was fixed at 2 μM, the fluorescence intensity gradually decreases with increasing sgc8-eNC concentration, indicating that a gradually increasing amount of Dox was loaded into sgc8-eNC since Dox fluorescence is quenched when intercalated into dsDNAs.[14, 45] No obvious difference in the fluorescence intensity between Line 350 pM and Line 400 pM demonstrates the complete intercalation of Dox into sgc8-eNC. The corresponding products are called Dox-sgc8-eNC.

Using the standard linear calibration curve, the amount of surplus Dox molecules was estimated from the recorded fluorescence peak by subtracting the residual fluorescence. The loaded drug amount was obtained by subtracting the surplus Dox amount from the total amount of Dox. Figure S41b shows the NC concentration-dependent fluorescence intensity recorded from Figure S41a and the dynamic relationship between the amount of loaded Dox and NC concentration, accompanied by the Dox-to-NC ratios at the high concentration. When the high concentration of sgc8-eNC is present (e.g., 350 nM, Dox-to-sgc8-eNC ratio of 5670:1), Dox fluorescence is substantially quenched as such no obvious fluorescence change is detected at the higher concentration (e.g., 400 pM) of sgc8-eNC. Dox-to-sgc8-eNC ratio of 5670:1 indicates an approximately 19 to 100-fold improved drug loading capacity compared with traditional DNA nanomaterials.[8, 14, 46, 47] To observe the encapsulation behavior of sgc8-eNC by naked eyes, the mixture at the same ratio (No. 2) was centrifuged and photographed, and free Dox solution under identical conditions was used as the control (No. 1). As shown in Inset, Sample 1 is brown and homogeneous, while there are clear red precipitates on the bottom of Tube 2 and the supernatant is basically colorless and transparent, proving that Dox molecules were almost completely loaded into sgc8-eNC. Thus, the Dox-to-sgc8-eNC ratio of 5670:1 was used in the subsequent experiments.

As can be seen from Figure S41c and S41d, the Dox loading behavior of sgc8-sNC and NC is roughly similar to sgc8-eNC. However, at the concentration of 350 pM, sgc8-sNC and bare NC possess the molar ratio of Dox-to-vehicle is 5593:1 and 5526:1, respectivley. The corresponding residual fluorescence intensities are 85.9 a.u. and 90.5 a.u., respectively, which are obviously higher than the residual fluorescence value (18.5 a.u.) of sgc8-eNC. This is reasonable because e-sgc8 aptamers installed in the internal cavity of sgc8-eNC induce the formation of additional ds DNA fragments and thus increase Dox loading capability to an extent. Namely, while sgc8-sNC and NC possess a desirable drug loading capability, sgc8-eNC exhibits the further improved encapsulation efficiency and loading efficiency.

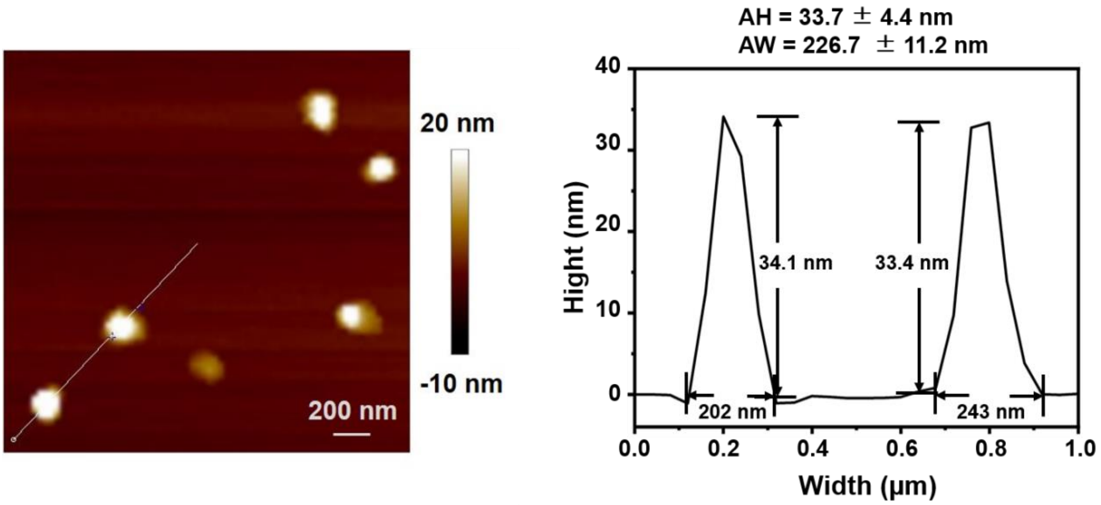

Figure S42. AFM characterization of Dox-sgc8-eNC. On the left is AFM image of Dox-sgc8-eNC prepared by inserting Dox into sgc8-eNC. On the right is the corresponding cross-section profile along the white line. The experimental procedure for preparation of Dox-sgc8-eNC is shown in the section of “Drug Loading”, while AFM measurement was performed according to the procedure described in the section of "AFM characterization of DNA nanocluster (NC)".

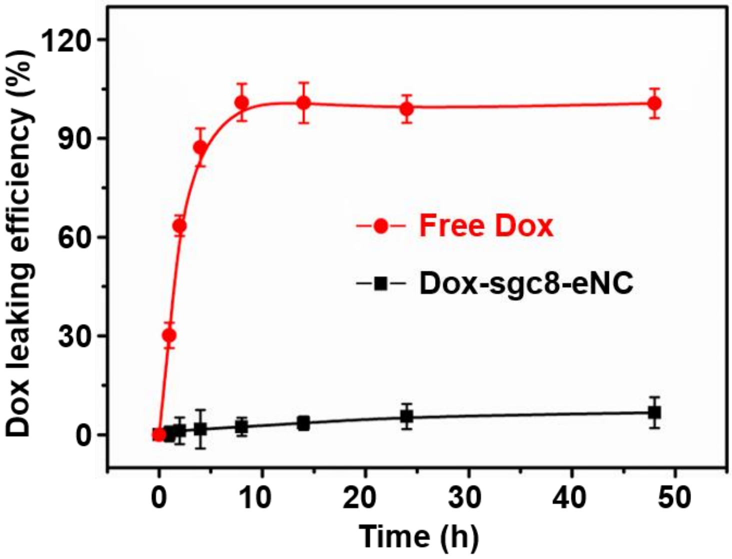

Figure S43. The stability of Dox-sgc8-eNC formulation. The black line represents the dynamic relationship between Dox leakage from Dox-sgc8-eNC formulation and incubation time. Equivalent concentration of free Dox was used as a control (red line). The error bars represent the standard deviation (n = 3). The working solution is PBS buffer (137 mM NaCl, 2.7 mM KCl, 10 mM Na_2HPO_4, 2 mM KH_2PO_4, pH = 7.4) (approximate pH of the blood).

Experimental procedure:

The stability of Dox-sgc8-eNC complexes was evaluated by measuring the amount of Dox leaking from Mini Dialysis Device (3.5K MWCO, Thermo Fisher, USA) when incubating in PBS buffer. Specifically, 100 μL of Dox-sgc8-eNC (3.5 nM; the equivalent concentration of Dox, 20 μM) was added into Mini Dialysis Device, and the same amount of free Dox was used as a control. The Mini Dialysis Device was immersed in PBS buffer and kept at room temperature with gentle shaking (200 RPM) under light-sealed conditions. At different time points (0, 1, 2, 4, 8, 14, 24, and 48 h), 200 μL of dialysate was taken out, and the fluorescence spectra were collected from 500 to 700 nm at the excitation wavelength of 495 nm on Hitachi F-7000 fluorescence spectrometer under the Xenon lamp as excitation light source (Hitachi Ltd., Japan). Then, the solution was placed back to make the original volume of dialysate unchanged.

Discussion:

The stability of Dox-sgc8-eNC was evaluated by a drug leakage experiment. The leakage efficiency (LE) of Dox is estimated according to the following formula:

$$LE= C_r/C_t \times 100\%$$

Where C_r indicates the cumulative amount of Dox released, while C_t represents the initial amount. C_r was quantified by comparing the fluorescence intensity recorded at a given time point to standard linear calibration curve (Figure S40). As shown in Figure S43, one can notice that free Dox can rapidly diffuse from dialysis bag into PBS buffer (red line), while the Dox loaded into sgc8-eNC almost does not leak (black line) even if keeping 48 h (only 6.7% cumulative lease), proving the desirable stability of Dox-sgc8-eNC in a physiological environment (pH 7.4).

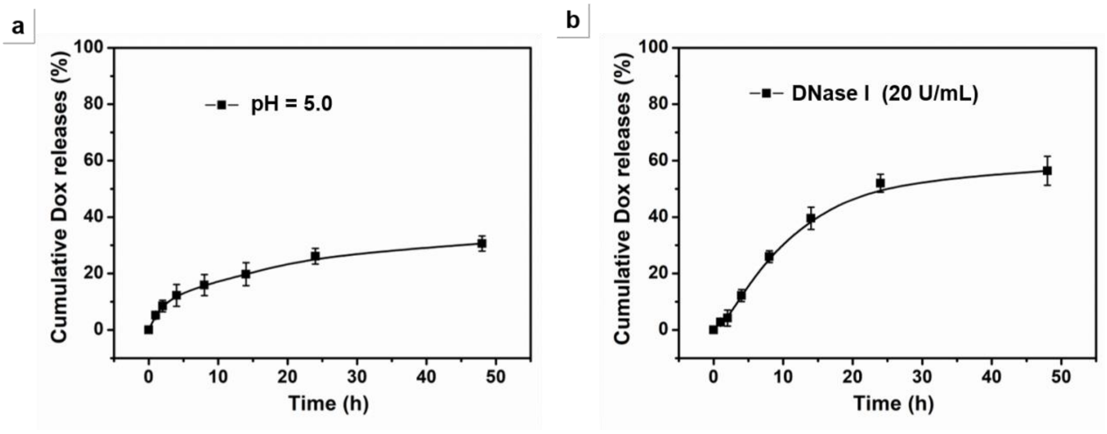

Figure S44. ***In vitro*** **cumulative release of Dox from Dox-sgc8-eNC in harsh biological conditions.** The release efficiency of Dox from sgc8-eNC in two different harsh biological solutions is described as follows: (a) PBS solution (pH 5.0), (b) PBS buffer (pH=7.4) in the presence of 20 U/mL DNase I. The corresponding experiments were conducted using the procedure similar to the leakage evaluation (Figure S43), but Dox-sgc8-eNC was instead incubated in the acidic buffer or DNase I solution. The error bars represent the standard deviation (n = 3).

Discussion:

The release efficiency of Dox drugs from the Dox-sgc8-eNC was evaluated under two harsh simulated endogenous conditions. Firstly, the PBS buffer (pH=5.0) was used to simulate the acidic microenvironment of tumors.[48] As indicated in Figure S44a, about 30% of Dox drugs can be released from sgc8-eNC carrier, which is much larger than the value measured in normal physiological environment (PH = 7.4, black line of Figure S43), indicating the stimuli-responsive release of Dox in an acidic tumor microenvironment. The similar experimental results were previously reported by a considerable number of literature studies.[49-52] This should be attributed to the probable influence of low pH on DNA base pair formation and Dox-DNA interaction,[13] enabling the release of loaded Dox drugs in acidic tumor microenvironment. Then, PBS buffer (pH=7.4) in the presence of DNase I at a very high concentration (20 U/mL) was used to simulate the enzymatic degradation release of physiological environment of normal organisms. As described in Figure S44 b, the amount of Dox released increases with the increment of incubation time and reaches 56% at 48 h, implying that the complex endogenous environment (e.g., intracellular environment) can initiate the release of Dox drugs from Dox-sgc8-eNC.

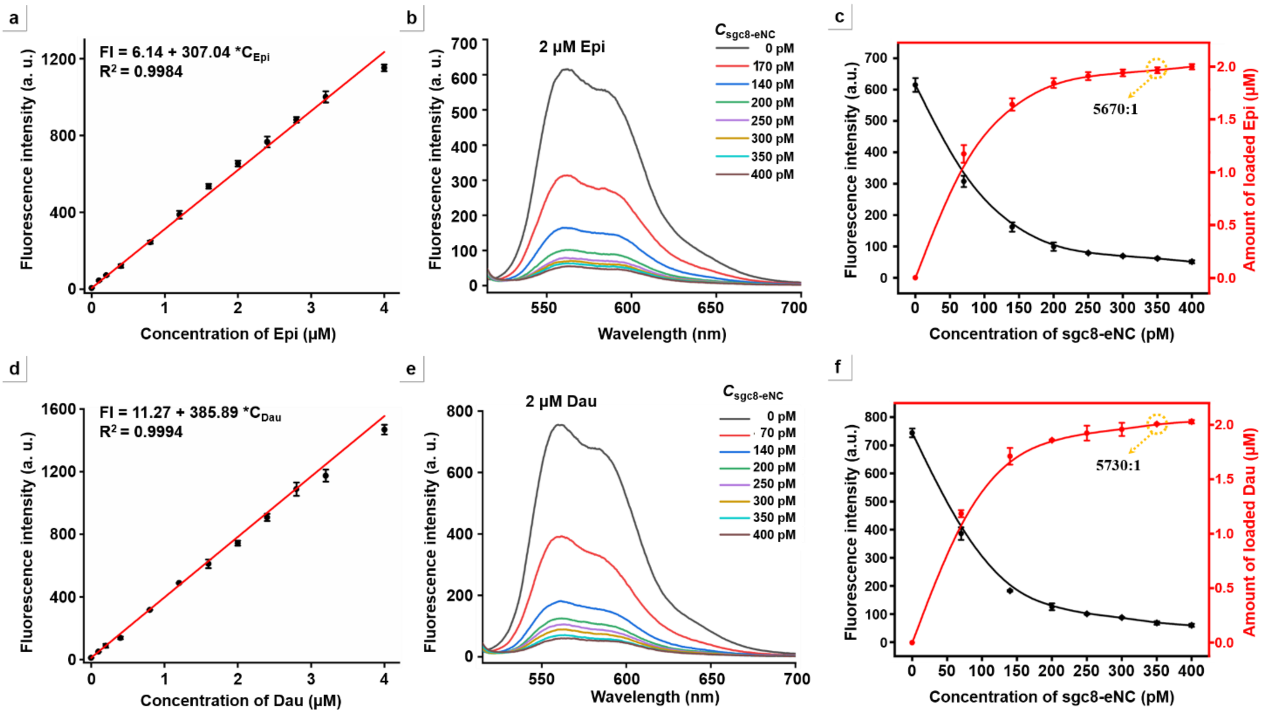

Figure S45. The universality of sgc8-eNC as an anticancer drug nanocarrier. (a) The standard linear calibration curve of fluorescence intensity of Epirubicin (Epi) at 560 nm versus its concentration. (b) Fluorescence spectrum of Epi (2 μM) in the presence of different concentrations of sgc8-eNC. The fluorescence of Epi is quenched when intercalating into the ds-DNA fragments in sgc8-eNC, forming Epi-sgc8-eNC. (c) Fluorescence peak intensity of Epi at 560 nm (black) and amount of Epi loaded (red) estimated from panel b. (d) The standard linear calibration curve of fluorescence intensity of Daunorubicin (Dau) at 560 nm versus its concentration. (e) The fluorescence spectrum of Dau (2 μM) co-existing with different concentrations of sgc8-eNC. Intercalation of Dau into ds-DNA fragments existing in sgc8-eNC causes the fluorescence quenching, forming Dau-sgc8-eNC. (f) Fluorescence peak intensity of Dau at 560 nm (black) and amount of Dau loaded (red) estimated from panel e.

Experimental procedure:

The experimental procedure for the preparation of Epi-sgc8-eNC and Dau-sgc8-eNC is the same as Dox-sgc8-eNC, but the Epi and Dau drugs were used to instead of Dox, respectively. Specifically, 2 μM Dau (or Epi) was incubated with different concentrations (0, 70, 140, 200, 250, 300, 350 and 400 pM) of sgc8-eNC and placed in a dry bath incubator at 37°C overnight with protection from light. The fluorescence spectrum of products was measured on Hitachi F-7000 fluorescence spectrometer (Hitachi, Ltd., Japan) and the data were analyzed using the manufacturer-supplied Origin software. The instrument parameters were given as follows: EX WL: 495.0 nm; EM Start WL: 500.0 nm; EM END WL: 700.0 nm; Scan speed: 240 nm/min; EX Slit: 5.0 nm; EM Slit: 5.0 nm; PMT Voltage: 800 V; Response: 2.0 s).

Discussion:

The universality of sgc8-eNC for therapeutic cargo encapsulation was assessed by exploring its capability to load Epi and Dau since the two molecules are important anticancer therapeutics and often used as drug models.[8, 47, 53-55] As shown in Figure S45a and S45d, the fluorescence intensity of Epi and Dau exhibits a good linear relationship with their own concentration. When Epi or Dau concentration is fixed at 2 μM, the fluorescence intensity gradually decreases with increasing sgc8-eNC concentration, indicating that the increasing amount of drugs is loaded into sgc8-eNC. No obvious change in fluorescence intensity is detected when further increasing the sgc8-eNC concentration from 350 pM to 400 pM (Figure S45b and S45e), demonstrating almost complete binding of Epi or Dau to sgc8-eNC at 350 pM. Based on the similar strategy to calculate the drug loading capability (described in Figure S41), the molar ratios of Epi-to-sgc8-eNC and Dau-to-sgc8-eNC are 5670:1 and 5730:1, respectively (Figure S45c and S45f).

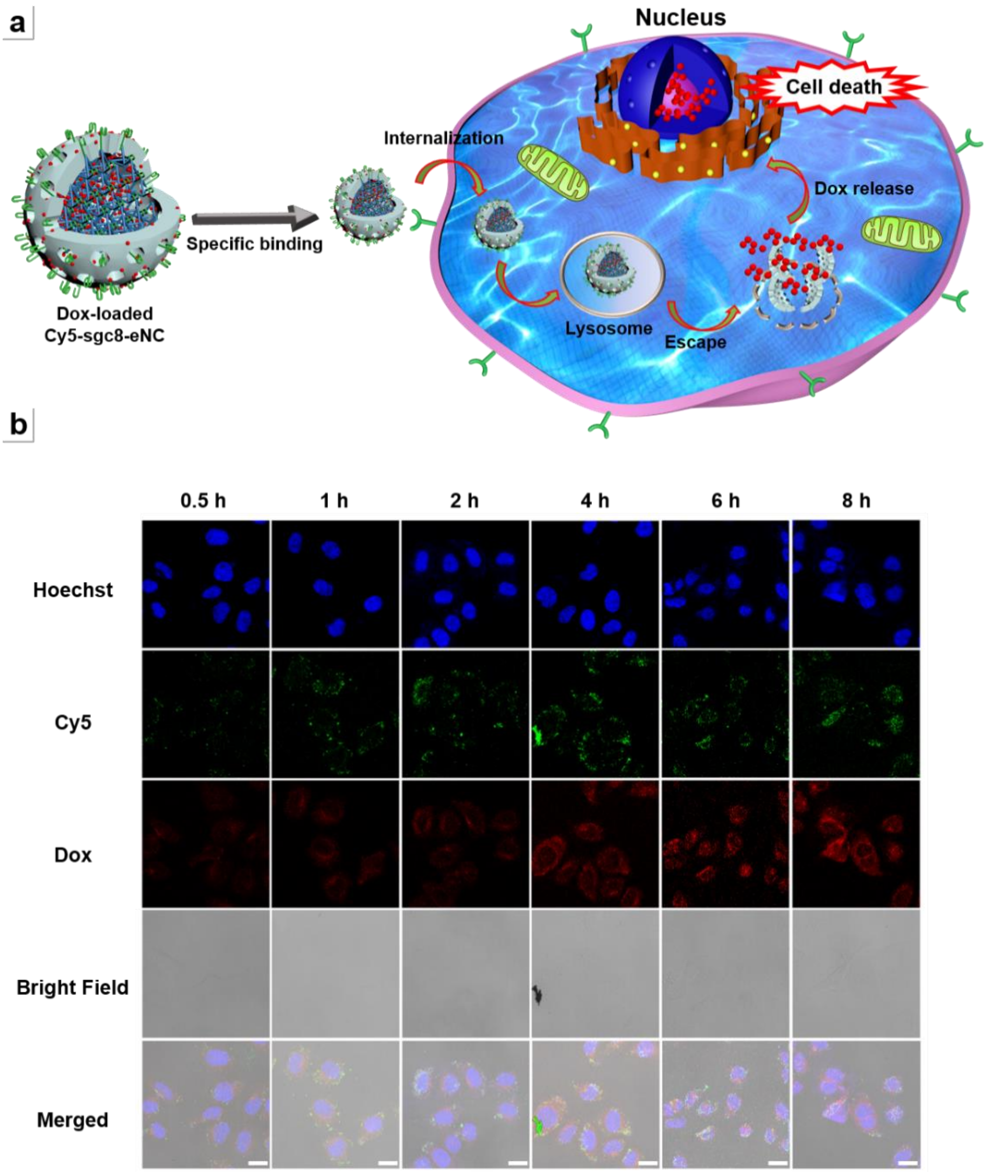

Figure S46. (a) Schematic diagram of targeted transport of anticancer drugs by Sgc8-eNC into the nuclei, during which several stages are involved: specific binding to cancer cell-surface receptor, cellular internalization, intracellular drug release, accumulation in nucleus and treatment-induced cell apoptosis. (b) CLSM imaging to explore the time-dependent drug internalization into HeLa cells. HeLa cells were treated with 350 pM Dox-loaded Cy5-sgc8-eNC. Scale bar = 20 µm.

Experimental procedure:

For CLSM imaging, 5×10^4 cells were cultured in a 24-well plastic plate with a round coverslip and grown to around 80% confluency. Then, the cells were incubated with Dox-loaded Cy5- sgc8-eNC (350 pM) for various time periods (0.5, 1, 2, 4, 6 and 8 h). After washing twice with PBS, the cells were fixed with 4% paraformaldehyde for 15 min at room temperature and stained with Hoechst 33342 for 5 min at room temperature. Subsequently, the Hoechst staining solution was removed, and the cells were washed twice with PBS. Finally, 2 µL of Antifade Mounting Medium was added dropwise onto the center of cover slip and placed upside down on a microscope slide. The fluorescence images of the resulting cells were acquired by a Leica SP8 laser scanning confocal microscope. Hoechst 33342 was excited at a wavelength of 405 nm, while Dox was excited at 488 nm and Cy5 was excited at 638 nm.

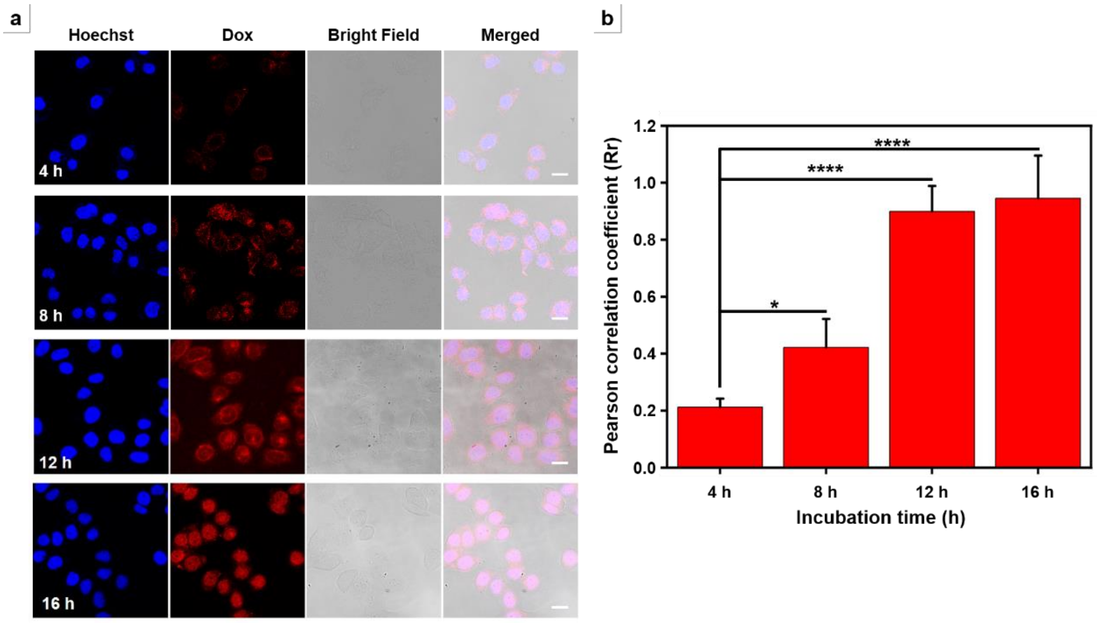

Figure S47. Fluorescence colocalization analysis to evaluate drug accumulation in the nuclei. (a) Fluorescence colocalization imaging of HeLa cells post-incubated with Dox-sgc8-eNC to observe the intracellular spatiotemporal distribution of Dox. Scale bar = 20 μm. (b) The quantitative evaluation of fluorescence colocalization using the Pearson's correlation coefficient. The HeLa cells were incubated with 350 pM Dox-sgc8-eNC, and CLSM imaging was performed at different time intervals. The statistical analysis of multiple comparisons were evaluated by one-way ANOVA. *P < 0.05, **P < 0.005, ***P < 0.0005, ****P < 0.0001.

Experimental procedure:

Cells were plated in a 14-mm cover glass in 24-well plastic-bottom plate and grown to around 80% confluency for subsequent experiments. Specifically, the cells were cultured in Dox-sgc8-eNC solution (2 μM Dox equivalents) for a given time period. After washing twice with PBS, the cells were fixed with 4% paraformaldehyde for 15 min and stained with Hoechst 33342 for 5 min at room temperature, followed by washing to remove Hoechst staining solution. Subsequently, 2 μL of Antifade Mounting Medium was added dropwise onto the center of cover slip and placed upside down on a microscope slide. The fluorescence images of the resulting cells were acquired by a Leica SP8 laser scanning confocal microscope. Hoechst 33342 was excited at a wavelength of 405 nm, while Dox was excited at 488 nm.

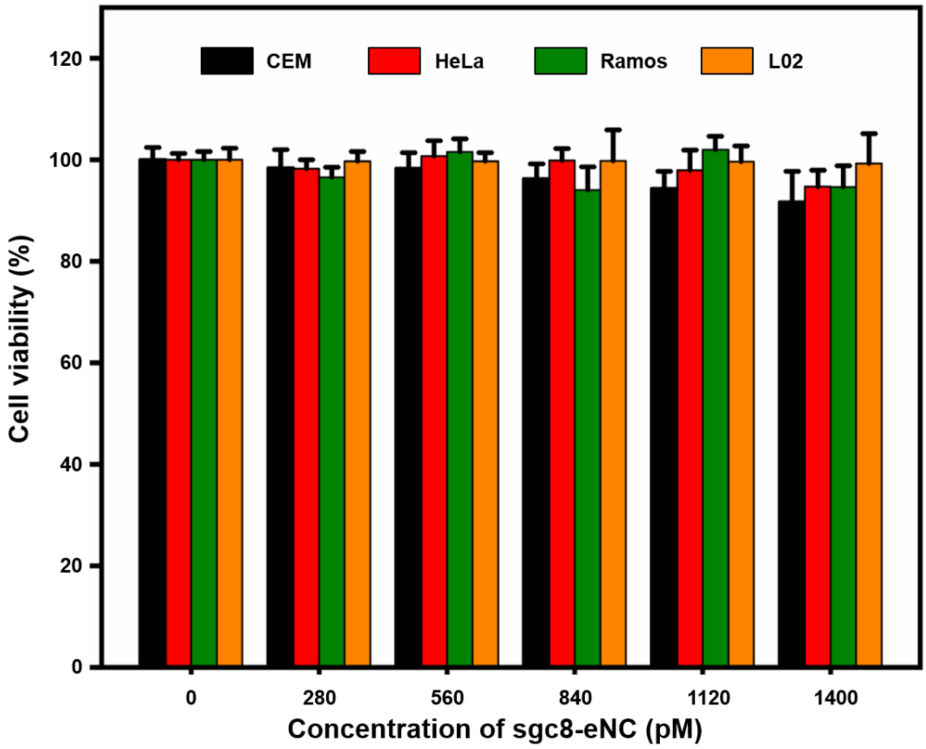

Figure S48. General toxicity of DNA NC nanostructures to different cells. CEM and HeLa cells were used as positive target cells, while Ramos and L02 cells served as negative control and normal cells, respectively. The black columns represent CEM cells, the red columns represent HeLa cells, the green columns represent Ramos cells, and the orange columns represent L02 cells. The error bars represent the standard deviation (n = 3).

Discussion:

In order to further verify the biocompatibility of sgc8-eNC, four types of cells were treated with NC nanostructures and the cell viability was evaluated by using the CCK-8 assay. As can be seen from Figure S48, when the concentration of sgc8-eNC was increased, there is no obvious change in the cell viability regardless of the nature of cells. Even if the concentration of sgc8-eNC reaches 1400 pM (the corresponding total amount of DNA components is approximately 3170 nM), the cell viability displays negligible decrease for all the cells (the lowest value is over 92%), indicating the excellent biocompatibility of sgc8-eNC.

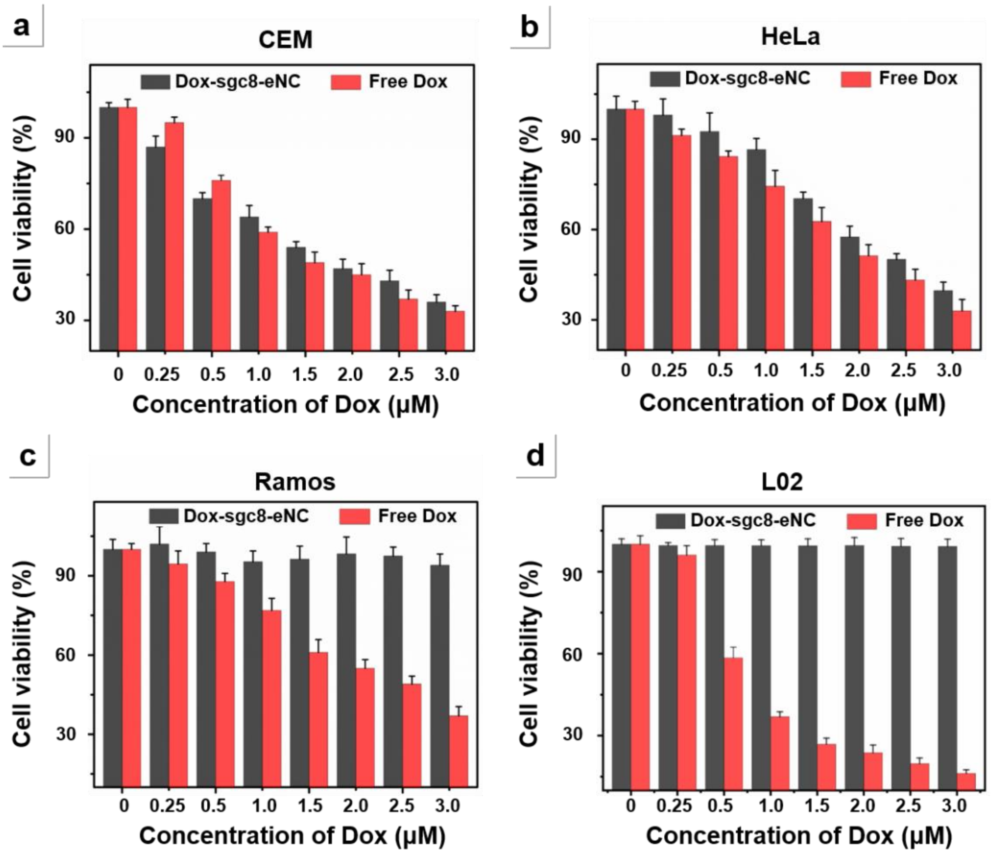

Figure S49. Cytotoxicity profiles of free Dox and Dox-sgc8-eNC measured by CCK-8 assay to assess the targeted therapeutic effect. Cell viability of CEM cells (a), HeLa cells (b), Ramos cells (c) and L02 cells (d) treated with free Dox or Dox-sgc8-eNC. Cell viability was measured under identical conditions. To highlight the therapeutic effect, the equivalent concentration of Dox rather than NC carrier concentration is represented in this section. Error bars denote standard deviations from three independent experiments. The experimental details are shown in the section of "Cytotoxicity analysis of DNA assembled nanostructures".

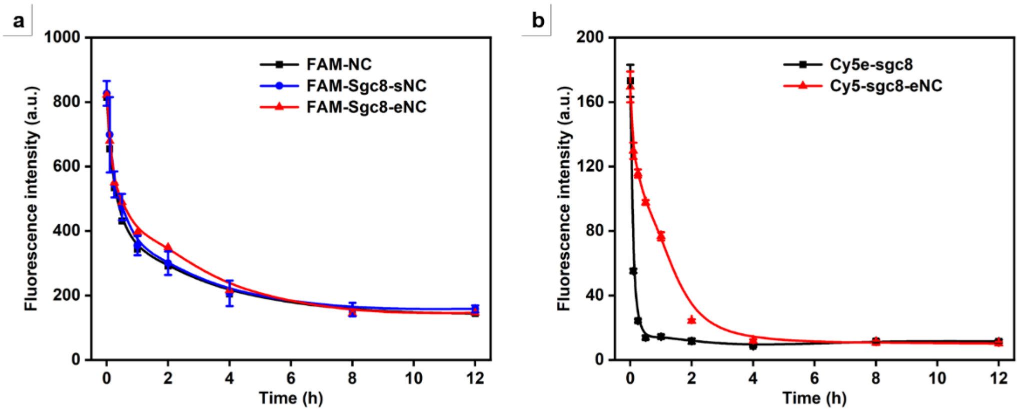

Figure S50. The in vivo half-life time of DNA NCs. (a) Fluorescence change of three different 3D DNA nanoclusters, including FAM-NC, FAM-sgc8-sNC and FAM-sgc8-eNC (each 500 nM, 200 μL), in the blood samples of healthy mice at different time points post-administration by tail-vein injection was measured to assess the blood half-life. For the three DNA NCs, to emit the fluorescence, Tb'-FAM component was used during the assembly. (b) Fluorescence change of Cy5-sgc8-eNC and Cy5e-sgc8 (each 500 nM, 200 μL) in the blood samples was measured under conditions identical to these used in panel a. During the assembly of Cy5-sgc8-eNC, Cy5e-sgc8 was used. The two experimental groups have the same concentration of Cy5e-sgc8. The error bar represents the standard deviation (SD). The measured data are expressed as the means ± SD (n = 5).

Experimental procedure:

For panel a: The healthy BALB/cJGpt mice (~20 g) were obtained from Fuzhou Wushi Animal Center and reared in a breeding cage with a sealed air filter at the temperature of 25-28°C and the relative humidity of 40-60%. The mice were randomly divided into three groups (at least 5 mice per group). An equal amount (500 nM, 200 μL) of FAM-sgc8-eNC, FAM-sgc8-sNC and FAM-NC (Tb'-FAM used instead in each) were separately administrated into the bodies of mice by tail-vein injection. At ten different time points post-injection (0, 0.1, 0.25, 0.5, 1, 2, 4, 8 and 12 h), orbital vein blood samples (200 μL) were acquired by standard capillary blood sampling procedure. Then, the plasma samples were obtained by centrifugation at 3500 rpm for 10 min at 4 ºC. The plasma (100 μL) and PBS (100 μL) was mixed, and the fluorescence intensity was measured on Hitachi F-7000 fluorescence spectrometer (Hitachi, Ltd., Japan).

For panel b: The experimental procedure is consistent with panel a and the same amount (500 nM, 200 μL) of Cy5-sgc8-eNC was involved, but Cy5e-sgc8 and label-free Tb' were used instead of label-free e-sgc8 and Tb'-FAM, respectively, during the assembly. Moreover, equal amount (500 nM, 200 μL) of Cy5e-sgc8 was used as control.

Discussion:

The blood half-life of sgc8-eNC in healthy BALB/c mice was evaluated in a comparative fashion. According to the literature method,[1, 56-58] an equal amount (500 nM, 200 μL) of FAM-sgc8-eNC, FAM-sgc8-sNC and FAM-NC were separately administrated into the bodies of different mice by tail-vein injection. The orbital vein blood was sampled for the fluorescence measurement at different time points post-injection. As shown in Figure S50a, FAM-sgc8-eNC dispalys an extended blood half-life compared with FAM-sgc8-sNC and FAM-NC. Specifically, the blood half-life ($t_{1/2}$) of FAM-sgc8-eNC is 0.92 h, which is 1.5 and 1.2 times longer than FAM-NC ($t_{1/2}$ = 0.63 h) and FAM-sgc8-sNC ($t_{1/2}$ = 0.75 h), respectively. To further verify the long blood half-life of sgc8-eNC, we also measured the blood half-life of equal amount of Cy5e-sgc8 and Cy5-sgc8-eNC. As shown in Figure S50b, the blood half-life of Cy5e-sgc8 is only 0.07 h, while the blood half-life of Cy5-sgc8-eNC is 0.82 h, indicating that, after installing in eNC, the blood half-life of sgc8 aptamer is extended by 11.7 times. The ratio of blood half-life of DNA tetrahedral material and single-strand material is only 1.9 [56] and 2.3 [57] times. Namely, compared to literature values, the blood half-life of sgc8-eNC has been greatly (5.1 ~ 6.2 times) improved.

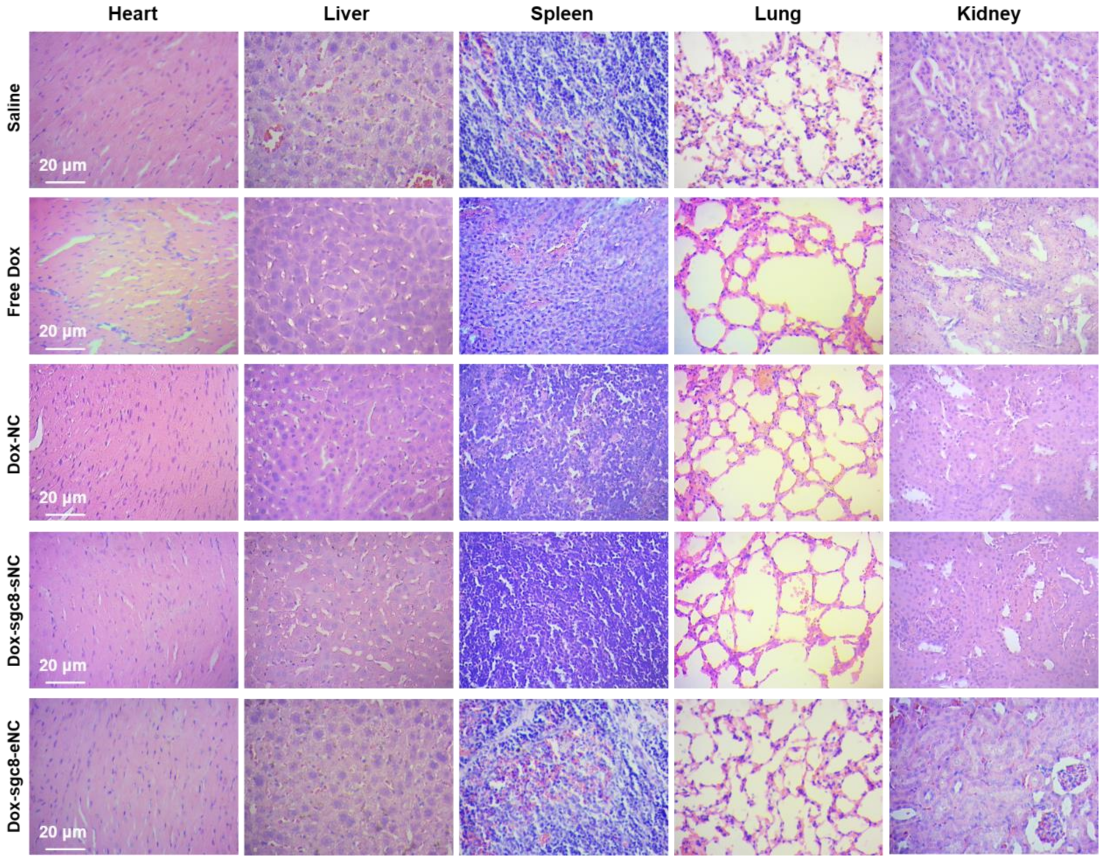

Figure S51. Histological analysis of hematoxylin and eosin (H&E) stained slices of major organs harvested from tumor-bearing mice administrated with different Dox-loaded DNA formulations. From the left to right are heart, liver, spleen, lung and kidney, while from the top to bottom are the groups of saline, free Dox, Dox-NC, Dox-sgc8-sNC and Dox-sgc8-eNC. The experimental procedure is described in the section of "Histopathological analysis by hematoxylin and eosin (H&E) staining". Scale bar = 20 μm.

Discussion:

The histological analysis shows that there is no obvious difference in the histological images of different organs, including the heart, liver, spleen, lung, and kidney, between Dox-sgc8-eNC group and saline group. Similar experimental results are observed for Dox-NC, Dox-sgc8-sNC, which is reasonable because their residual parts are easily excreted from the body by liver and kidney as described in Figure 4. In contrast, free Dox causes the damage to normal tissues, for example, heart, where the histological morphology changes and extracellular space increment is observed. No detectable damage toward the five organs demonstrates that Dox- sgc8-eNC has no obvious side effect or toxicity toward the normal tissues and holds the potential for targeted cancer therapy.

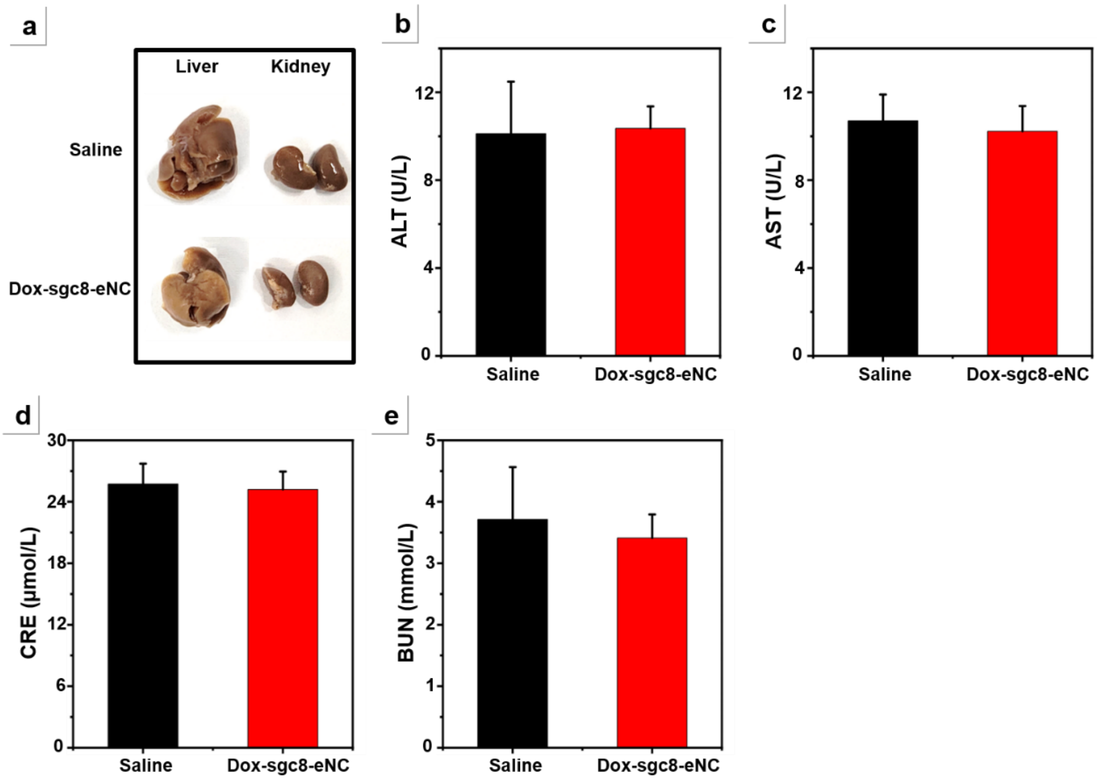

Figure S52. The liver and kidney toxicity of tumor-bearing mice caused during Dox-sgc8-eNC-based treatment. (a) The photos of liver and kidney harvested from tumor-bearing mice subjected to 18-day multiple-dose treatment with Dox-sgc8-eNC. The saline group was used as control. Quantitative analysis of serological markers of tumor-bearing mice treated with Dox-sgc8-eNC (or saline), including alanine transferase (ALT, b), aspartate aminotransferase (AST, c), creatinine (CRE, d) and blood urea nitrogen (BUN, e).

Experimental procedure:

After 18-day multiple-dose Dox-sgc8-eNC-based treatment, the tumor-bearing mice were anesthetized, and the blood samples were collected. The mice were sacrificed, and liver and kidney were harvested for photographing via phone (Honor, China). After addition of 1/10 volume of sodium citrate (3.2%) and centrifugation (3500 RPM, 10 min), the serum samples were obtained. The serum levels of alanine transferase (ALT), aspartate aminotransferase (AST), creatinine (CREA) and blood urea nitrogen (BUN) were measured by using alanine transferase test kit (ALT), aspartate aminotransferase test kit (AST), creatinine test kit (CREA) or blood urea nitrogen test kit (BUN) according to the manufacturer’s protocol. All test kits were purchased from Nanjing Jiancheng Bioengineering Institute, Nanjing, China.

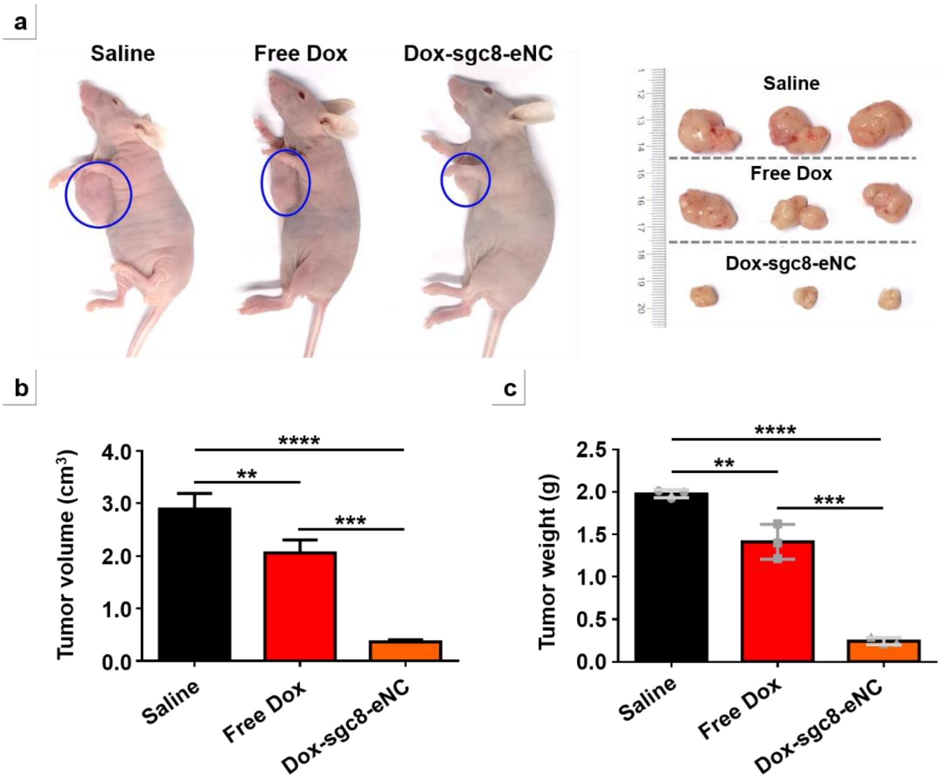

Figure S53. Inhibition of tumor growth of Dox-sgc8-eNC in HeLa tumor-bearing BALB/c nude mouse model (female). (a) Photos of tumor-bearing mice subjected to different treatments, including saline, free Dox and Dox-sgc8-eNC. The right part shows the photos of dissected tumors. (b) Tumor volume of mice after 18 days systemically post-administrated separately with saline, free Dox, and Dox-sgc8-eNC, and (c) corresponding tumor weights. The measured data are expressed as the means ± SD. The statistical analysis of multiple comparisons were evaluated by one-way ANOVA. $^{**}P < 0.005$, $^{***}P < 0.0005$, $^{****}P < 0.0001$.

Discussion:

As can be seen from Figure S53a, whole-animal *in vivo* images and *ex vivo* tumor images show that Dox-sgc8-eNC has a significant tumor suppressive effect compared to free Dox-based treatment groups. The corresponding tumor volume and tumor weight are quantitatively evaluated in Figure S53b and Figure S53c, respectively. One can notice that, compared with Saline group, the tumor growth is inhibited by about 88% for Dox-sgc8-eNC group, which is consistent with the measured results represented in Figure 5. This is possibly because, for subcutaneous xenograft model of HeLa cells, the tumor growth growth/suppression behavior is independent of mouse estrogen.

Section E: Supplementary References

[1] C. Xue, S. Hu, Z. H. Gao, L. Wang, M. X. Luo, X. Yu, B. F. Li, Z. Shen, Z.-S. Wu, *Nat. Commun.* **2021**, *12*, 2928. DOI: 10.1038/s41467-021-23250-5

[2] Liang, C.; Guo, B.; Wu, H.; Shao, N.; Li, D.; Liu, J.; Dang, L.; Wang, C.; Li, H.; Li, S.; Lau, W.; Cao, Y.; Yang, Z.; Lu, C.; He, X.; Au, DW.; Pan, X.; Zhang, B. -T.; Lu, C.; Zhang, H.; Yue, K.; Qian, A.; Shang, P.; Xu, J.; Xiao, L.; Bian, Z.; Tan, W.; Liang, Z.; He, F.; Zhang, L.; Lu, A.; Zhang, G. *Nat. Med.* **2015**, *21*, 288-294. DOI: 10.1038/nm.3791

[3] J. W. Conway, C. Madwar, T. G. Edwardson, C. K. McLaughlin, J. Fahkoury, R. B. Lennox, H. F. Sleiman, *J. Am. Chem. Soc.* **2014**, *136*, 12987-12997. DOI: 10.1021/ja506095n

[4] C. Zhang, Y. He, M. Su, S. H. Ko, T. Ye, Y. Leng, X. Sun, A. E. Ribbe, W. Jiangh, C. Mao, *Faraday Discuss.* **2009**, *143*, 221-233. DOI: 10.1039/b905313c

[5] Y. Dong, C. Yao, Y. Zhu, L. Yang, D. Luo, D. Yang, *Chem. Rev.* **2020**, *120*, 9420-9481. DOI: 10.1021/acs.chemrev.0c00294

[6] S. H. Um, J. B. Lee, N. Park, S. Y. Kwon, C. C. Umbach, D. Luo, *Nat. Mater.* **2006**, *5*, 797-801. DOI: 10.1038/nmat1741

[7] C. Xue, S. Zhang, C. Li, X. Yu, C. Ouyang, Y. Lu, Z.-S. Wu, *Anal. Chem.* **2019**, *91*, 15678-15685. DOI: 10.1021/acs.analchem.9b03784

[8] Y. Gao, Q. Li, J. Zhang, C. Wu, Z. Shen, C. Xue, H. T. Chang, Z.-S. Wu, *ACS Appl. Mater. Interfaces* **2020**, *12*, 3341-3353. DOI: 10.1021/acsami.9b16249

[9] H. Lee, A. K. Lytton-Jean, Y. Chen, K. T. Love, A. I. Park, E. D. Karagiannis, A. Sehgal, W. Querbes, C. S. Zurenko, M. Jayaraman, C. G. Peng, K. Charisse, A. Borodovsky, M. Manoharan, J. S. Donahoe, J. Truelove, M. Nahrendorf, R. Langer, D. G. Anderson, *Nat. Nanotechnol.* **2012**, *7*, 389-393. DOI: 10.1038/nnano.2012.73

[10] N. Y. Wong, C. Zhang, L. H. Tan, Y. Lu, *Small* **2011**, *7*, 1427-1430. DOI: 10.1002/smll.201100140

[11] Y. Qiao, Y. Lin, Y. Wang, Z. Yang, J. Liu, J. Zhou, Y. Yan, J. Huang, *Nano Lett.* **2009**, *9*, 4500-4504. DOI: 10.1021/nl9028335

[12] G. F. Schneider, S. W. Kowalczyk, V. E. Calado, G. Pandraud, H. W. Zandbergen, L. M. Vandersypen, C. Dekker, *Nano Lett.* **2010**, *10*, 3163-3167. DOI: 10.1021/nl102069z

[13] C. Wu, D. Han, T. Chen, L. Peng, G. Zhu, M. You, L. Qiu, K. Sefah, X. Zhang, W. Tan, *J. Am. Chem. Soc.* **2013**, *135*, 18644-18650. DOI: 10.1021/ja4094617

[14] C. Xue, S. Zhang, X. Yu, S. Hu, Y. Lu, Z.-S. Wu, *Angew. Chem. Int. Ed.* **2020**, *59*, 17540-17547. DOI: 10.1002/anie.202004805

[15] J. W. Conway, C. K. McLaughlin, K. J. Castor, H. Sleiman, *Chem. Commun.* **2013**, *49*, 1172-1174. DOI: 10.1039/c2cc37556g

[16] J. Li, H. Pei, B. Zhu, L. Liang, M. Wei, Y. He, N. Chen, D. Li, Q. Huang, C. Fan, *ACS Nano* **2011**, *5*, 8783-8789. DOI: 10.1021/nn202774x

[17] C. Li, C. Xue, J. Wang, M. Luo, Z. Shen, Z.-S. Wu, *Anal. Chem.* **2019**, *91*, 11529-11536. DOI: 10.1021/acs.analchem.9b00860

[18] C. Li, M. Luo, J. Wang, H. Niu, Z. Shen, Z.-S. Wu, *ACS Sens.* **2020**, *5*, 2378-2387. DOI: 10.1021/acssensors.0c00212

[19] A. P. Thomas, L. Palanikumar, M. T. Jeena, K. Kim, J. H. Ryu, *Chem. Sci.* **2017**, *8*, 8351-8356. DOI: 10.1039/c7sc03169f

[20] Q. Sun, Z. Kang, L. Xue, Y. Shang, Z. Su, H. Sun, Q. Ping, R. Mo, C. Zhang, *J. Am. Chem. Soc.* **2015**, *137*, 6000-6010. DOI: 10.1021/jacs.5b01435

[21] N. W. Kam, Z. Liu, H. Dai, *Angew. Chem. Int. Ed.* **2006**, *45*, 577-581. DOI: 10.1002/anie.200503389

[22] J. Chen, S. Chen, X. Zhao, L. Kuznetsova, S. Wong, I. Ojima, *J. Am. Chem. Soc.* **2008**, *130*, 16778-16785. DOI: 10.1021/ja805570f

[23] L. Liang, J. Li, Q. Li, Q. Huang, J. Shi, H. Yan, C. Fan, *Angew. Chem. Int. Ed.* **2014**, *53*, 7745-7750. DOI: 10.1002/anie.201403236

[24] M. Koivusalo, C. Welch, H. Hayashi, C. C. Scott, M. Kim, T. Alexander, N. Touret, K. M. Hahn, S. Grinstein, *J. Cell. Biol.* **2010**, *188*, 547-563. DOI: 10.1083/jcb.200908086

[25] Y. Yamada, S. M. Perez, M. Tabata, J. Abe, Y. Yasuzaki, H. Harashima, *J. Pharm. Sci.* **2015**, *104*, 2845-2854. DOI: 10.1002/jps.24310

[26] E. Dudleenamjil, C. Y. Lin, D. Dredge, B. K. Murray, R. A. Robison, F. B. Johnson, *J. Gen. Virol.* **2010**, *91*, 3032-3041. DOI: 10.1099/vir.0.024133-0

[27] S. Zhang, C. Chen, C. Xue, D. Chang, H. Xu, B. J. Salena, Y. Li, Z.-S. Wu, *Angew. Chem. Int. Ed.* **2020**, *59*, 14584–14592. DOI: 10.1002/anie.202005624

[28] Y. Dong, Y. R. Yang, Y. Zhang, D. Wang, X. Wei, S. Banerjee, Y. Liu, Z. Yang, H. Yan, D. Liu, *Angew. Chem. Int. Ed.* **2017**, *56*, 1586-1589. DOI: 10.1002/anie.201610133

[29] Hsu, N.; Nouri, B.; Chen, L.; Chen, H, *Macromolecules* **2020**, *53*, 9665−9675. DOI: 10.1021/acs.macromol.0c01445

[30] Huang, X.; Li, S.; Huang, Y.; Wu, S.; Zhou, X.; Li, S.; Gan, C.; Boey, F.; Mirkin, C.; Zhang, H. *Nat. Commun.* **2011**, *2*, 292. DOI: 10.1038/ncomms1291

[31] Wang, Y.; Deng, Q.; Fujita, N.; Han, L. *Chem. Mater.* **2020**, *32*, 5236−5245. DOI: 10.1021/acs.chemmater.0c01382

[32] H. Liu, X. Shen, Z.-G. Wang, A. Kuzyk, B. Ding, *Nanoscale* **2014**, *6*, 9331-9338. DOI: 10.1039/C3NR06913C

[33] Y. He, T. Ye, M. Su, C. Zhang, A. E. Ribbe, W. Jiang, C. Mao, *Nature* **2008**, *452*, 198-201. DOI: 10.1038/nature06597

[34] S. Saha, D. Bhatia, Y. Krishnan, *Small* **2010**, *6*, 1288-1292. DOI: 10.1002/smll.201000330

[35] K. Zhang, W. Huang, Y. Huang, H. Li, K. Wang, X. Zhu, M. Xie, *Anal. Chem.* **2019**, *91*, 7086-7096. DOI: 10.1021/acs.analchem.9b00011

[36] H. Zhao, M. Wang, X. Xiong, Y. Liu, X. Chen, *Talanta* **2020**, *210*, 120677. DOI: 10.1016/j.talanta.2019.120677

[37] Y. -R. Chen, S. Sun, H. Yin, W. Wang, R. Liu, H. Xu, Y. Yang, Z.-S. Wu, *J. Mater. Chem. B.* **2022**, *10*, 1969-1979. DOI: 10.1039/d1tb01978c

[38] C. A. Hong, A. A. Eltoukhy, H. Lee, R. Langer, D. G. Anderson, Y. S. Nam, *Angew. Chem. Int. Ed.* **2015**, *54*, 6740-6744. DOI: 10.1002/anie.201412493

[39] L. Wang, Q. Sun, X. Wang, T. Wen, C. Chen, *J. Am. Chem. Soc.* **2015**, *137*, 1947–1955. DOI: 10.1021/ja511560b

[40] H. Huang, Z. Guo, C. Zhang, C. Cui, T. Fu, Q. Liu, W. Tan, *ACS Appl. Mater. Interfaces* **2021**, *13*, 30397-30403. DOI: 10.1021/acsami.1c07632

[41] X. Jin, Q. Zeng, J. Zheng, D. Xing, T. Zhang, *ACS Appl. Mater. Interfaces* **2021**, *13*, 9316-9328. DOI: 10.1021/acsami.0c14730

[42] H. Xiong, J. Yan, S. Cai, Q. He, N. Wen, Y. Wang, Y. Hu, D. Peng, Y. Liu, Z. Liu, *Mol. Pharm.* **2020**, *17*, 2882-2890. DOI: 10.1021/acs.molpharmaceut.0c00335

[43] Q. Li, D. Zhao, X. Shao, S. Lin, X. Xie, M. Liu, W. Ma, S. Shi, Y. Lin, *ACS Appl. Mater. Interfaces* **2017**, *9*, 36695-36701. DOI: 10.1021/acsami.7b13328

[44] J. Li, C. Zheng, S. Cansiz, C. Wu, J. Xu, C. Cui, Y. Liu, W. Hou, Y. Wang, L. Zhang, I. T. Teng, H. H. Yang, W. Tan, *J. Am. Chem. Soc.* **2015**, *137*, 1412-1415. DOI: 10.1021/ja512293f

[45] R. Mo, T. Jiang, R. DiSanto, W. Tai, Z. Gu, *Nat. Commun.* **2014**, *5*, 3364. DOI: 10.1038/ncomms4364

[46] N. Chen, S. Qin, X. Yang, Q. Wang, J. Huang, K. Wang, *ACS Appl. Mater. Interfaces* **2016**, *8*, 26552-26558. DOI: 10.1021/acsami.6b08695

[47] G. Zhu, J. Zheng, E. Song, M. Donovan, K. Zhang, C. Liu, W. Tan, *Proc. Natl. Acad. Sci. USA* **2013**, *110*, 7998-8003. DOI: 10.1073/pnas.1220817110

[48] L. Feng, Z. Dong, D. Tao, Y. Zhang, Z. Liu, *National Science Review* **2018**, *5*, 269-286. DOI: 10.1093/nsr/nwx062

[49] M. Chang, C. S. Yang, D. M. Huang, *ACS Nano* **2011**, *5*, 6156-6163. DOI: 10.1021/nn200693a

[50] Q. Jiang, C. Song, J. Nangreave, X. Liu, L. Lin, D. Qiu, Z. G. Wang, G. Zou, X. Liang, H. Yan, B. Ding, *J. Am. Chem. Soc.* **2012**, *134*, 13396-13403. DOI: 10.1021/ja304263n

[51] K.-R. Kim, D.-R. Kim, T. Lee, J. Y. Yhee, B.-S. Kim, I. C. Kwon, D.-R. Ahn, *Chem. Commun.* **2013**, *49*, 2010-2012. DOI: 10.1039/C3CC38693G

[52] J. Liu, L. Song, S. Liu, S. Zhao, Q. Jiang, B. Ding, *Angew. Chem. Int. Ed.* **2018**, *57*, 15486-15490. DOI: 10.1002/anie.201809452

[53] T. Tan, H. Wang, H. Cao, L. Zeng, Y. Wang, Z. Wang, J. Wang, J. Li, S. Wang, Z. Zhang, Y. Li, *Adv. Sci.* **2018**, *5*, 1801012. DOI: 10.1002/advs.201801012

[54] M. S. Han, A. K. Lytton-Jean, C. A. Mirkin, *J. Am. Chem. Soc.* **2006**, *128*, 4954-4955. DOI: 10.1021/ja0606475

[55] I. Matai, A. Sachdev, P. Gopinath, *ACS Appl. Mater. Interfaces* **2015**, *7*, 11423-11435. DOI: 10.1021/acsami.5b02095

[56] D. Jiang, Y. Sun, J. Li, Q. Li, M. Lv, B. Zhu, T. Tian, D. Cheng, J. Xia, L. Zhang, L. Wang, Q. Huang, J. Shi, C. Fan, *ACS Appl. Mater. Interfaces* **2016**, *8*, 4378-4384. DOI: 10.1021/acsami.5b10792

[57] W. Ma, Y. Zhan, Y. Zhang, X. Shao, X. Xie, C. Mao, W. Cui, Q. Li, J. Shi, J. Li, C. Fan, Y. Lin, *Nano Letter* **2019**, *19*, 4505-4517. DOI: 10.1021/acs.nanolett.9b01320

[58] Z. Zhang, M. M. Ali, M. A. Eckert, D. K. Kang, Y. Y. Chen, L. S. Sender, D. A. Fruman, W. Zhao, *Biomaterials* **2013**, *34*, 9728-9735. DOI: 10.1016/j.biomaterials.2013.08.079
